# Identification of *CD161* expression as a novel prognostic biomarker in breast cancer correlated with immune infiltration

**DOI:** 10.3389/fgene.2022.996345

**Published:** 2022-09-30

**Authors:** Miaomiao Weng, Hui Xie, Mingjie Zheng, Xinwen Hou, Shui Wang, Yue Huang

**Affiliations:** ^1^ Department of Breast Surgery, The First Affiliated Hospital, Nanjing Medical University, Nanjing, China; ^2^ Department of Clinical Laboratory, The Affiliated Wuxi Maternity and Child Health Care Hospital of Nanjing Medical University, Wuxi, China

**Keywords:** CD161, breast cancer, prognostic biomarker, immune infiltration, function enrichment analysis

## Abstract

**Background:** *CD161* has been identified as a prognostic biomarker in many neoplasms, but its role in breast cancer (BC) has not been fully explained. We aimed to investigate the molecular mechanism and prognostic value of *CD161* in BC.

**Methods:** *CD161* expression profile was extracted from TIMER, Oncomine, UALCAN databases, and verified by the Gene Expression Omnibus (GEO) database and quantitative real-time polymerase chain reaction (qRT-PCR). The prognostic value of *CD161* was assessed *via* GEPIA, Kaplan–Meier plotter and PrognoScan databases. The Cox regression and nomogram analyses were conducted to further validate the association between *CD161* expression and survival. Gene set enrichment analysis (GSEA), Gene Ontology (GO) analysis, and KEGG pathway enrichment analysis were performed to probe the tumor-associated annotations of *CD161*. CIBERSORT and ssGSEA were employed to investigate the correlation between *CD161* expression and immune cell infiltration in BC, and the result was verified by TIMER and TISIDB.

**Results:** Multiple BC cohorts showed that *CD161* expression was decreased in BC, and a high *CD161* expression was associated with a preferable prognosis. Therefore, we identified the combined model including *CD161*, age and PR status to predict the survival (C index = 0.78) of BC patients. Functional enrichment analysis indicated that *CD161* and its co-expressed genes were closely related to several cancerous and immune signaling pathways, suggesting its involvement in immune response during cancer development. Moreover, immune infiltration analysis revealed that *CD161* expression was correlated with immune infiltration.

**Conclusion:** Collectively, our findings revealed that *CD161* may serve as a potential biomarker for favorable prognosis and a promising immune therapeutic target in BC.

## Introduction

Breast cancer (BC) is the most common cancer in females worldwide. Despite great advances in the diagnosis and treatment, BC relapses in a considerable number of patients. BC still accounts for 15.5% of all female cancer deaths (Pondé et al., 2019; Godeau et al., 2021; Sung et al., 2021). Currently, the treatment decisions and survival outcomes for BC patients mainly depend on the clinicopathological stage and type (Werner et al., 2022). However, patients with the same tumor stage, molecular subtype, and treatment regimens may have completely different clinical outcomes (Barry et al., 2010; Zardavas et al., 2015; Liu et al., 2022). This indicates that the existing staging system is not sufficient for accurate prognosis prediction, and the typing system cannot totally depict the tumor heterogeneity. More personalized treatment strategies and prognostic biomarkers based on tumors’ intrinsic characteristics are urgently needed. Therefore, digging deeply into biological characteristics of breast tumor may help better predict clinical outcomes and develop novel therapies for BC. Tumors consist of not only neoplastic cells, but also a dynamic surrounding stroma (Olson and Joyce, 2013; Bejarano et al., 2021; Xie et al., 2021). Emerging evidence suggests that BC is characterized by a highly inflammatory tumor microenvironment (TME), which is supported by the tumor infiltrating immune cells (TILs), cytokines, and growth factors *etc*. (Lim et al., 2018). Crosstalk between cancer cells and TILs continually influences the occurrence, development, and metastasis of breast tumors (Flister and Bergom, 2018; Lim et al., 2018; Li et al., 2020). TILs have been proved to be related to therapeutic response and can serve as novel therapeutic targets (Stanton and Disis, 2016; Byrne et al., 2020). Previous reports have supported that a high density of TILs is an important prognostic factor to improve the survival outcomes of patients (Savas et al., 2018; Maibach et al., 2020; Paijens et al., 2021). However, one common observation from functional studies is that many subsets of TILs failed to expand or function normally in the BC microenvironment, instead contributing to the tumor progression (Guo and Deng, 2018; Yang et al., 2020). Accordingly, identifying the factors that affect the dynamic changes of TILs at the gene level is crucial for targeted BC treatment and improved prognosis outcomes. CD161, encoded by killer cell lectin-like receptor B1 (*KLRB1*), is a C-type lectin-related type II transmembrane protein expressed on NK cells, NKT cells and subsets of CD8^+^ and CD4^+^ T cells (Braud et al., 2022). It was identified as favorable prognostic gene in most human cancers (Braud et al., 2018; Duurland et al., 2022). *CD161* can act as a costimulatory receptor to increase the response to T cell receptor (TCR) stimulation (Fergusson et al., 2014). Therefore, in oropharyngeal squamous cell cancer (OPSCC), CD4^*+*^CD161^+^ T cells display a stronger type 1 response to suboptimal antigen stimulation and produce more cytokines upon antigen stimulation in, resulting in a better prognosis (Welters et al., 2018; Duurland et al., 2022). In addition, CD161 binds to CLEC2D/LLT1 to inhibit NK-mediated cytotoxicity in target cells (López-Soto et al., 2017). A recent study has discovered that the knockdown of *KLRB1* or antibody-mediated blockade of *CD161* enhances the ability of T cells to kill tumor cells (Mathewson et al., 2021), suggesting that the CD161-LLT1 pathway may serve as a potential target of immunotherapy for glioma. However, due to the heterogeneity of BC, the tumorigenic effects and clinical significance of abnormal expression of *CD161* in BC remain largely unknown. In this study, we utilized the BC RNA-seq data from The Cancer Genome Atlas (TCGA) and Gene Expression Omnibus (GEO) to compare the expression of *CD161* in normal breast tissues and BC samples. Then, we verified the findings by quantitative real-time polymerase chain reaction (qRT-PCR), using paired tissue samples from our center. Next, we assessed the relationship between *CD161* expression and clinicopathological parameters of BC. Furthermore, we investigated the prognostic value of *CD161* for BC, and predicted BC survival using a nomogram constructed with the independent prognostic factors derived from multivariate Cox regression analysis. Besides, we conducted functional enrichment analyses to probe the tumor-associated annotations of *CD161*. Finally, we analyzed the relationship between *CD161* expression and immune infiltration. Our research indicated that *CD161* could serve as a potential prognostic biomarker and immune therapeutic target for BC.

## Materials and methods

### Patients and sample collection

A total of 1097 BC patients from the TCGA database were enrolled. Included were patients with (Pondé et al., 2019) a primary site at the breast; (Godeau et al., 2021) data in the Program of TCGA; (Sung et al., 2021) data in the Project of TCGA-BRCA; (Werner et al., 2022) primary tumor tissue sampled. The clinicopathological parameters and RNA-seq data of these patients were downloaded and summarized in Supplementary Table S1 for further analysis. Furthermore, the paired tissue samples of 20 patients diagnosed as invasive BC without distant metastasis were randomly obtained from the breast center of the First Affiliated Hospital with Nanjing Medical University. The clinicopathological data for these patients are presented in Supplementary Table S2. After surgical resection, the tissues were preserved in RNA*later* ^™^ solution (ThermoFisher, United States), immediately frozen in liquid nitrogen and storage at −80°C until further use for qRT-PCR. The use of human tissues in this study was approved by the Research Ethics Committee and all patients enrolled signed the informed consent.

### qRT-PCR

Total RNA was extracted from the paired breast tumors and adjacent tissue samples by Olasma Kit (QIAGEN, Germany). cDNA was prepared through The HiScript^®^ III first Strand cDNA Synthesis Kit (+gDNA wiper) (Vazyme, Nanjing, China). Next, qRT-PCR was performed using the ChamQ SYBR qPCR Master Mix (Vazyme, Nanjing, China). The primers used were as follows: human *CD161* -Forward: 5′-AAA​CAA​CAG​AGA​GAC​CGG​GT-3′; human *CD161* -Reverse: 5′-TCC​AAG​GGT​TGA​CAG​TGT​GAT-3′; human *GAPDH*-Forward: 5′-GGA​GTC​CAC​TGG​CGT​CTT​CA-3′; human *GAPDH*-Reverse, 5′-GTC​ATG​AGT​CCT​TCC​ACG​ATA​CC-3′. Primers were synthesized by the GenScript Company.

### *CD161* expression

The expression of *CD161* in BC was investigated by TIMER (https://cistrome.shinyapps.io/timer/), Oncomine (http://www.oncomine.org), UALCAN (http://ualcan.path.uab.edu), and validated by GSE10797 from GEO database and qRT-PCR of paired tissue samples. The binary logistic model was employed to explore the association between *CD161* expression and clinicopathologic features, including stage, age, T, N, M, ER, PR, menopause and anatomic neoplasm subdivision downloaded from TCGA database.

### Survival analysis

The GEPIA (http://gepia.cancer-pku.cn/), Kaplan–Meier plotter (http://kmplot.com/analysis) and PrognoScan (http://dna00.bio.kyutech.ac.jp/PrognoScan/index.html) were employed to evaluate the prognostic value of *CD161* in BC, based on the data about mRNA expression and survival. Overall survival (OS) referred to the time from histological diagnosis to death or the last follow-up. Disease-free survival (DFS) was the time from histological diagnosis to disease progression, death, or last follow-up. Disease-specific survival (DSS) was defined as the time from histological diagnosis to death from BC. Besides, we conducted univariate and multivariate Cox analyses of BC data from TCGA. A number of variables were assessed to identify the independent prognostic factors, including stage, age, tumor size, lymph node status, distant metastasis, *CD161* expression, ER status and PR status.

### Construction and validation of the nomogram

A nomogram was conducted based on the independent prognostic factors identified by the multivariate Cox analysis. Calibration plots were estimated to assess the predictive power of the nomogram, and the C-index was calculated to evaluate the discriminative ability of the nomogram.

### Functional enrichment analysis

Gene Set Enrichment Analysis (GSEA) is usually used to determine the statistical significance of a priorly defined set of genes and evaluate the difference between two biological subsets (Subramanian et al., 2007). We used GSEA software to classify the pathways enriched in different BC phenotypes based on the expression level of *CD161*. Genomes with false discovery rate (FDR) < 0.05 were considered remarkedly enriched. cBioPortal for Cancer Genomics (http://cbioportal.org) was used to identify the genes co-expressing with *CD161*. Then, we performed Gene Ontology (GO) and KEGG pathway analysis to obtain the functional annotations of these co-expressed genes.

### Evaluation of tumor-infiltrating immune cells

All 1097 BC patients enrolled from TCGA database were divided into high and low *CD161* expression groups based on the cutoff value of 50%. CIBERSORT was utilized to compare the proportions of 22 tumor infiltration immune cells in both groups. Then, the immune infiltration levels of 24 cell types in BC were downloaded from published literature. ssGSEA analysis was performed to assess the association between *CD161* expression and immune infiltration. TISIDB (http://cis.hku.hk/TISIDB/), TIMER and GEPIA were used to verify this association.

### Study design

Our study was designed according to the reporting guideline checklist Tripod (Supplementary Table S3). The RNA-seq data and clinicopathological data of 1097 BC patients from TCGA database were retrospectively reviewed. Using 50% of the *CD161* expression value as a cutoff point, all patients were divided into high *CD161* and low *CD161* expression groups for survival analysis, GSEA analysis and immune infiltration analysis. The follow-up threshold of OS, DFS and DSS were displayed in the survival curve.

### Statistical analysis

All statistical analyses were conducted using R-4.1.2. In stratification analysis, the case was deleted from the data set when the stratified variable was missing. The paired sample *t*-test was performed to evaluate the difference in *CD161* expression between paired tissues. The binary logistic model was performed by R package ISLR. The Cox regression and nomogram analyses of survival were conducted by R package survival, ggforest and RMS. The GO and KEGG analyses were accomplished by R package clusterProfiler. The CIBERSORT R script and R package GSVA were used to evaluate the immune infiltration. All the tests were two-sided, and *p* < 0.05 was defined as statistically significant.

## Results

### Expression profiles of *CD161*

**FIGURE 1:**
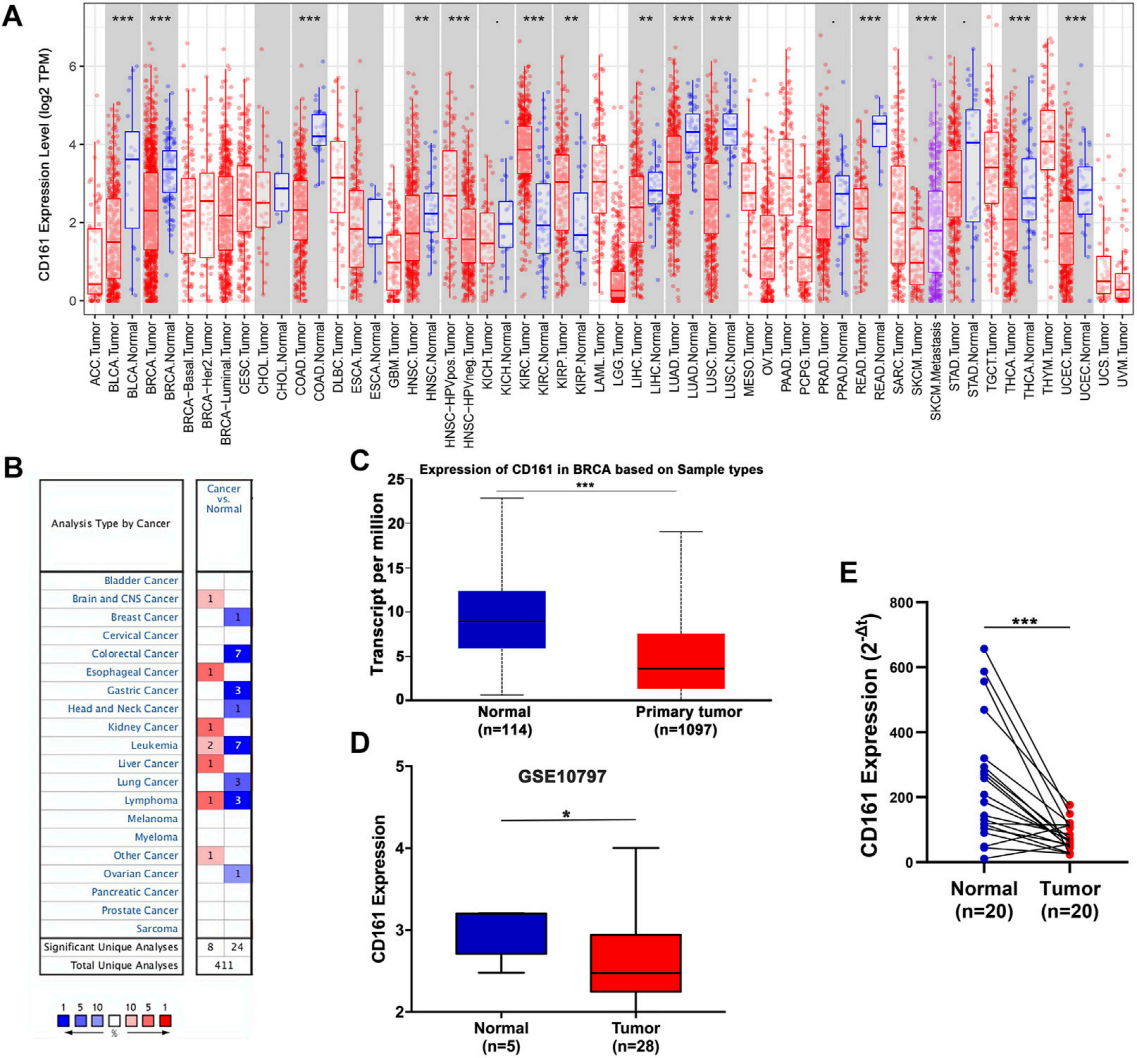
The mRNA expression levels of *CD161* in various human cancers. **(A)** Differential expression of *CD161* between paired tumor and normal tissues in various human cancers from the TIMER database. **(B)** Meta-analysis of *CD161* expression between paired tumor and normal tissues across various human cancers from the Oncomine database. **(C)** Comparison of *CD161* expression between breast tumor and normal samples in UALCAN database. **(D)** Comparison of *CD161* expression between breast tumor and normal tissues in GEO GSE10797 datasets. **(E)** qRT-PCR results of *CD161* expression between paired breast tumor and normal tissue samples from our cohort, *n* = 20. GEO, Gene Expression Omnibus. *p < 0.05, **p < 0.01, ***p < 0.001.

TIMER-based analysis showed that the mRNA expression of *CD161* was lower in the tissue samples of most malignant tumors, like bladder cancer (BLCA), breast invasive carcinoma (BRCA), colon adenocarcinoma (COAD) (Figure 1A). Oncomine, UALCAN and GEO databases (GSE10797) further confirmed the decreased transcriptome level of *CD161* in BC tissue samples (Figures 1B–D). This difference was further validated by qRT-PCR for 20 paired tumorous and adjacent tissue samples (Figure 1E). The clinicopathological information of the 20 patients enrolled are summarized in Supplementary Table S2.

### Correlation of *CD161* expression with clinicopathological characteristics

All 1097 BC patients were divided into high and low *CD161* expression groups, based on the cutoff value of 50%. The case was deleted from the data set when the stratified variable was missing. The binary logistic model showed that lower *CD161* expression was associated with higher T stage (T2&T3&T4 vs T1, OR = 0.613, *p* < 0.001, N = 1080), distant metastasis (M1 vs M0, OR = 0.327, *p* = 0.032, N = 922), and higher pathologic stage (StageⅡ & StageⅢ & StageⅣ vs StageⅠ, OR = 0.642, *p* = 0.007, N = 1060). Meanwhile, there was no statistical correlation between *CD161* expression and other clinicopathological parameters, like lymph node stage, PR status, ER status, and anatomic neoplasm subdivision (Table 1).

**TABLE 1:** The correlation between *CD161* expression and clinicopathological characteristics in BC.

Characteristics	Total (N)	Odds ratio (OR)	*p* Value
T stage (T2&T3&T4 vs T1)	1,080	0.613 (0.464–0.807)	**<0.001**
N stage (N1&N2&N3 vs N0)	1,064	1.233 (0.969–1.569)	0.088
M stage (M1 vs M0)	922	0.327 (0.106–0.853)	**0.032**
Pathologic stage (Stage II&Stage III&Stage IV vs Stage I)	1,060	0.642 (0.463–0.886)	**0.007**
PR status (Positive vs Negative)	1,030	0.900 (0.694–1.167)	0.427
ER status (Positive vs Negative)	1,033	0.779 (0.582–1.040)	0.091
Anatomic neoplasm subdivisions (Right vs Left)	1,083	1.089 (0.858–1.382)	0.484

The bold values are means *p* < 0.05 was defined as statistically significant which was highlighted in bold type.

### Prognostic value of *CD161* in BC

**FIGURE 2:**
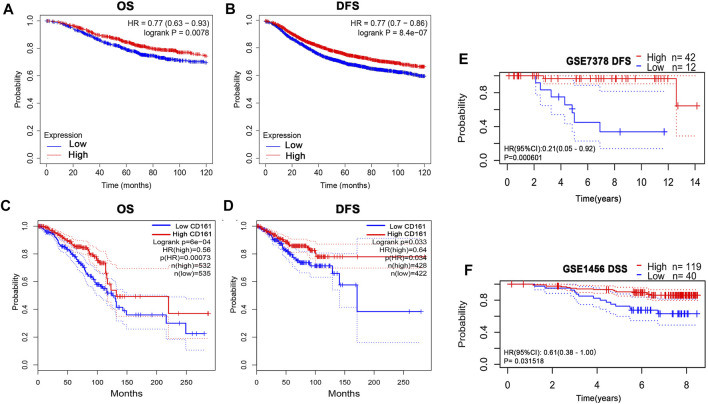
Kaplan-Meier assessment for survival outcomes according to *CD161* expression in BC patients. **(A)** The OS estimate of high *CD161* expression survivors was higher than low *CD161* expression survivors from Kaplan–Meier Plotter database (*p* < 0.01). **(B)** The DFS estimate of high *CD161* expression survivors was higher than low *CD161* expression survivors from Kaplan–Meier Plotter database (*p* < 0.001). **(C)** The OS estimate of high *CD161* expression survivors was higher than low *CD161* expression survivors from GEPIA database (*p* < 0.001). **(D)** The DFS estimate of high *CD161* expression survivors was higher than low *CD161* expression survivors from GEPIA database (*p* = 0.03). **(E)** High *CD161* expression survivors had improved DFS compared with low *CD161* expression survivors in GSE7378 from PrognoScan database (*p* < 0.001).**(F)** High *CD161* expression survivors had improved DSS compared with low *CD161* expression survivors in GSE1456 from PrognoScan database (*p* = 0.03). BC, breast cancer; OS, overall survival; DFS, disease free survival; DSS, disease-specific survival.

**FIGURE 3:**
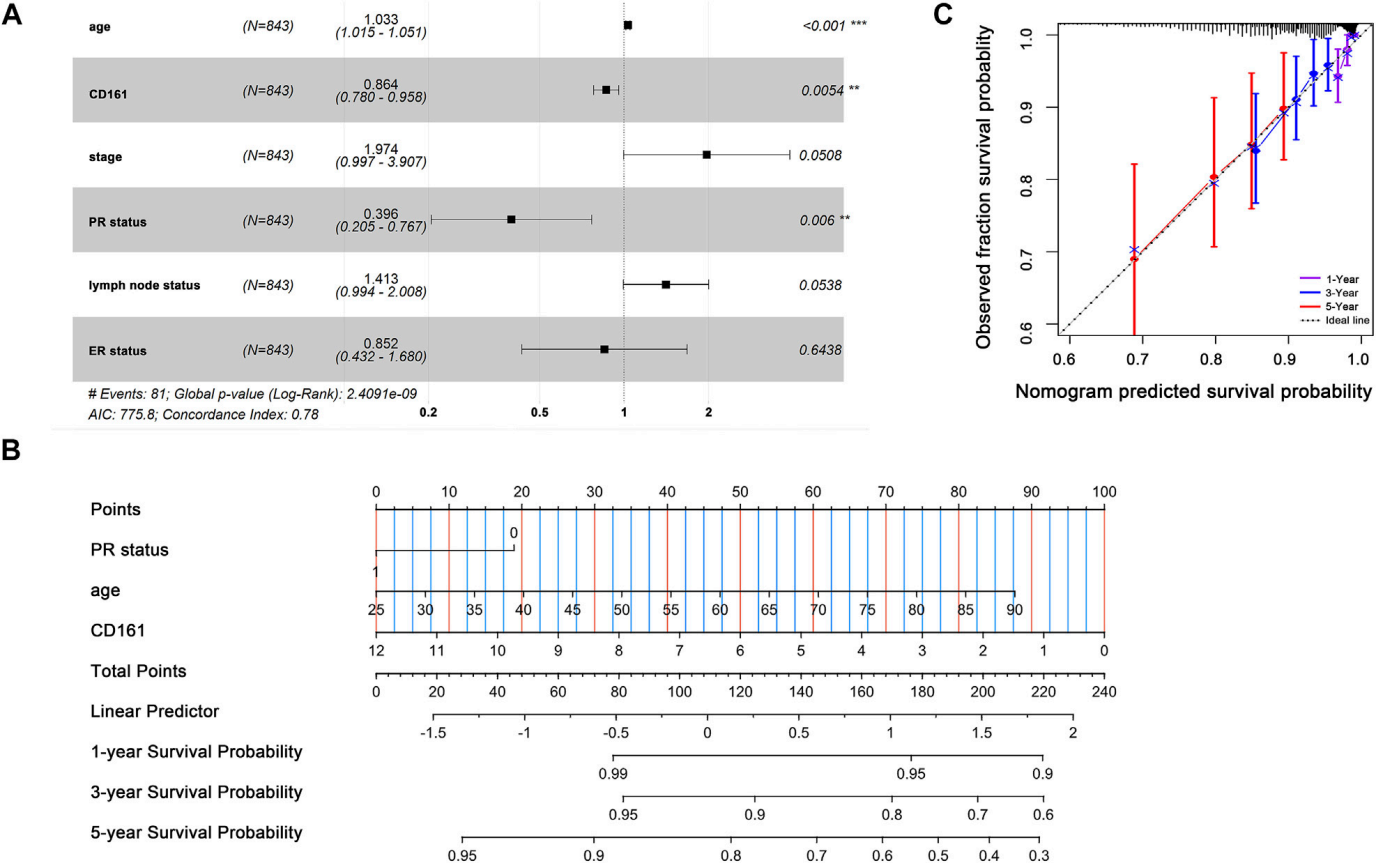
Prognostic value of *CD161* expression in BC patients. **(A)** Multivariate Cox analyses on variables for the prediction of overall survival of BC patients. The effect of PR status, age and *CD161* expression on survival was statistically significant. The results were demonstrated as a forest plot. **(B)** A constructed nomogram for prognostic prediction of BC patients. For PR status, 0 represented PR-negative status and 1 represented PR-positive status. The importance of each variable was ranked according to the standard deviation along nomogram scales. To use the nomogram, the specific points of individual patients were located on each variable axis. Lines and dots were drawn upward to determine the points received by each variable; the sum of these points was located on the Total Points axis to determine the probability of 1-year, 3-year and 5-year OS. **(C)** Calibration curves of the nomogram. The dotted line indicated the ideal reference line where predicted probabilities would match the observed survival rates. The dots were calculated by bootstrapping (resample: 1000) and represented the performance of the nomogram. The closer the solid lines were to the dotted line, the more accurately the model predicted survival. The purple solid line, blue solid line and red solid line represented the calibration curve of 1-year OS, 3-year OS and 5-year OS separately. OS, overall survival; BC, breast cancer; PR, progesterone receptor. *p < 0.05, **p < 0.01, ***p < 0.001.

Based on the RNA-seq data and clinicopathological data from TCGA, the prognostic value of *CD161* expression in BC was evaluated by Kaplan-Meier plotter database and GEPIA database. The results showed that higher *CD161* expression was significantly related to prolonged survival (OS, HR = 0.56, *p* < 0.001; DFS, HR = 0.77, *p* < 0.001; OS, HR = 0.77, *p* < 0.001; DFS, HR = 0.64, *p* = 0.033) (Figures 2A–D). We next verified the favorable prognostic value of *CD161* in BC by PrognoScan database with GEO data (GSE7378, DFS, HR = 0.21, *p* < 0.001; GSE1456, DSS, HR = 0.61, *p* = 0.032) (Figures 2E,F). Moreover, the univariate and multivariate Cox analysis suggested that *CD161* expression (HR = 0.864, *p* = 0.05), PR status (HR = 0.396, *p* = 0.006), and age (HR = 1.029, *p* < 0.001) were independent prognostic factors for OS in BC (Table 2 and Figure 3A). Therefore, we constructed a nomogram to predict the overall survival probability in BC based on *CD161* expression, PR status and age (Figure 3B). The calibration plots and C-index (0.78) implied an outstanding predictive and discriminative power of the nomogram (Figure 3C).

**TABLE 2:** Univariate Cox analysis and multivariate Cox analysis for OS.

Variable	Univariate Cox regression		Multivariate Cox regression	
	Hazard ratio (95% CI)	*p* Values	Hazard ratio (95% CI)	*p* Values
Stage	2.548 (1.638–3.965)	<0.001	1.974 (0.918–3.907)	0.051
Age	1.024 (1.007–1.041)	0.006	1.029 (1.015–1.051)	<0.001
T (tumor size)	1.439 (0.877–2.360)	0.15	—	—
N (lymph node status)	1.701 (1.345–2.151)	<0.001	1.413 (0.994–2.008)	0.054
M (distant metastasis)	2.162 (0.992–4.711)	0.052	—	—
CD161	0.888 (0.803–0.982)	0.021	0.864 (0.780–0.958)	0.005
ER status	0.600 (0.377–0.953)	0.031	0.852 (0.432–1.680)	0.644
PR status	0.537 (0.346–0.834)	0.006	0.396 (0.205–0.767)	0.006

### Function enrichment analysis

**FIGURE 4:**
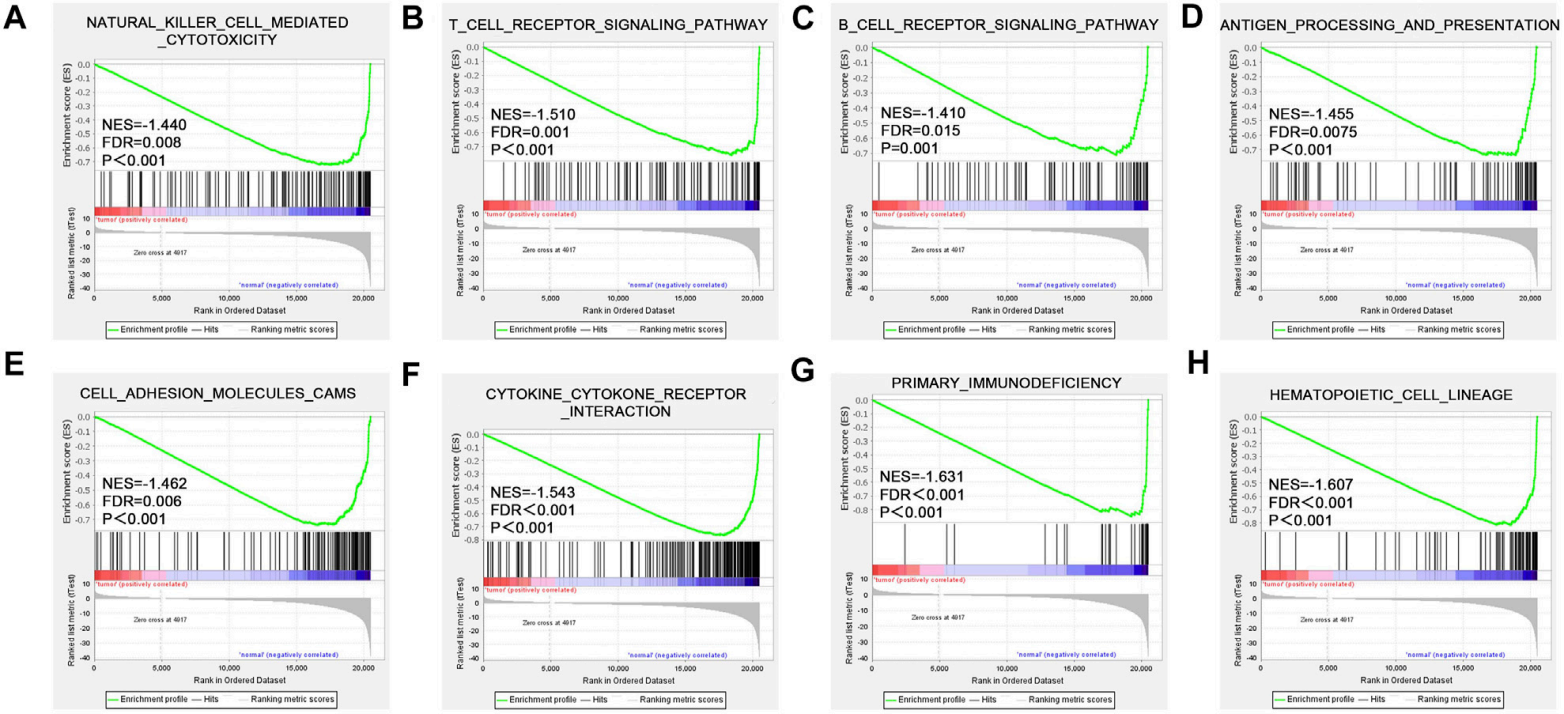
Enriched pathways of *CD161* expression in BC analyzed by GSEA. Up-regulated genes located on the left approaching the origin of the coordinates, and down-regulated genes lay on the right of *x*-axis. Only gene sets with *p* < 0.05 and FDR <0.05 were considered significant. Top eight significant pathways associated with low *CD161* expression were displayed in the plot. **(A)** NK cell-mediated cytotoxicity pathway, **(B)** T cell receptor signaling pathway, **(C)** B cell receptor signaling pathway, **(D)** antigen processing and presentation, **(E)** cell adhesion molecules cams, **(F)** cytokine-cytokine receptor interaction, **(G)** primary immunodeficiency, **(H)** hematopoietic cell lineage. BC, breast cancer; GSEA, gene set enrichment analysis; NES, normalized enrichment score; FDR, false discovery rate.

**FIGURE 5:**
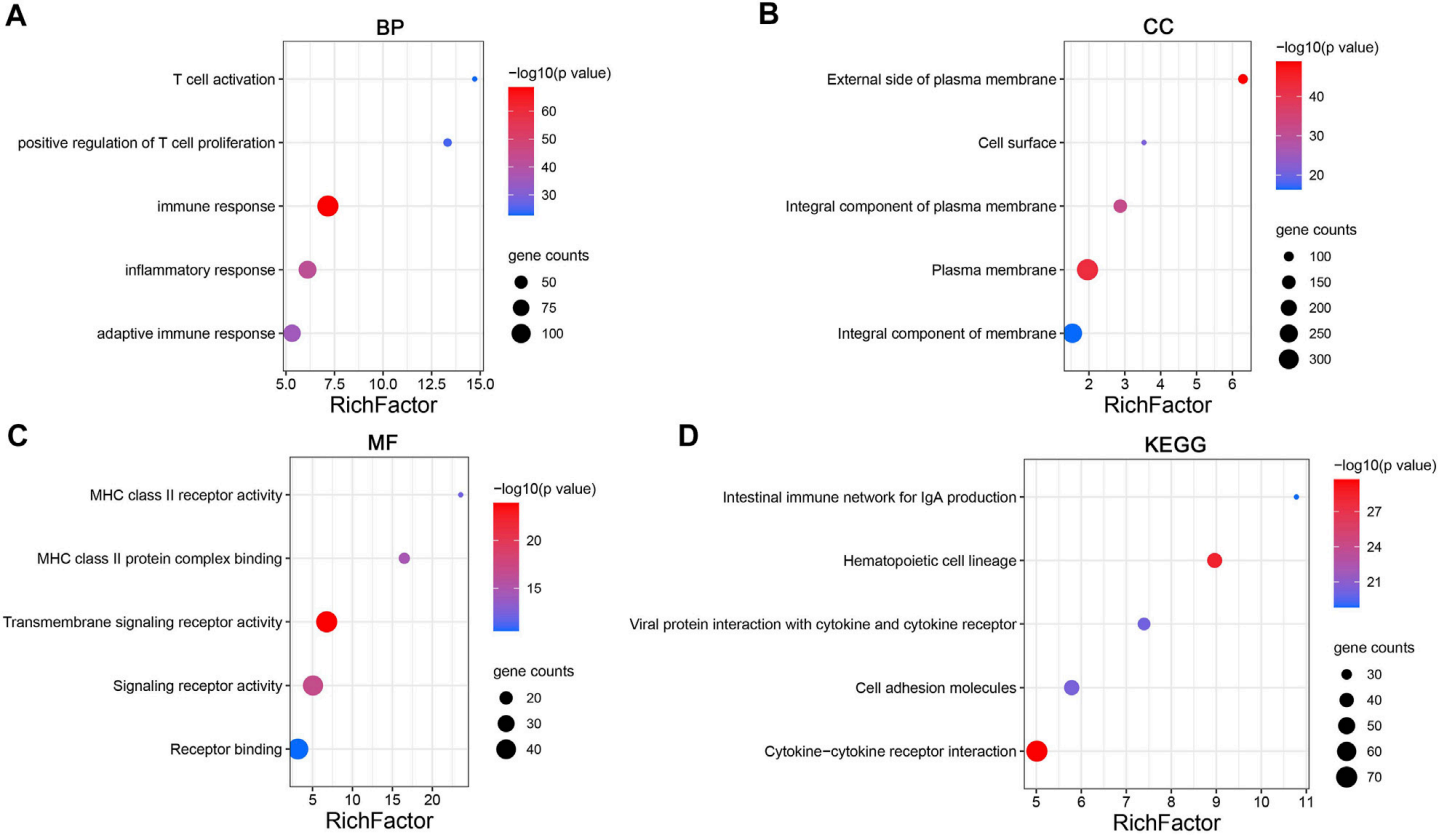
Enrichment analysis of *CD161* co-expression gene network in BC. **(A**–**C)** Bubble plots displayed the top 5 BP, MF and CC terms that were significantly associated with *CD161* co-expression genes. **(D)** Bubble plots displayed the top 5 KEGG enrichment pathways that were significantly associated with *CD161* co-expression genes. *X*-axis: the ratio of genes belonged to the corresponding terms/pathways versus the whole co-expression genes. *Y*-axis: the top 5 GO terms/significant enrichment pathways. The color gradient of the bubble referred to the enrichment *p*-value, and the size of the bubble referred to the number of co-expression genes in corresponding GO terms/pathways. BP, biological progress; CC, cellular component; MF, molecular function; GO, Gene Ontology.

To explore the molecular mechanism of *CD161* in BC, we performed GSEA analysis in high and low *CD161* expression groups, and GO and KEGG enrichment analyses in *CD161* co-expressed genes. In the GSEA analysis, NK cell-mediated cytotoxicity pathway, T cell receptor signaling pathway, B cell receptor signaling pathway, antigen processing and presentation, cell adhesion molecules cams, cytokine-cytokine receptor interaction, primary immunodeficiency, and hematopoietic cell lineage were significantly enriched in *CD161* low expression group, according to NES, NOM *p* values, and FDR values (Figure 4). The GO and KEGG enrichment analyses were performed based on 728 co-expressed genes screened out of the cBioPortal database with |Spearman’s correlation| >0.5 and *p* < 0.05 (Supplementary Table S4) (Schober et al., 2018). GO analysis showed that *CD161* co-expressed genes were enriched in immune responses, especially T cell-related adaptive immune response. They acted as structural constituents in the plasma membrane, and were involved in signaling receptor activity (Figures 5A–C). KEGG pathway analysis showed their enrichment in pathways of hematopoietic cell lineage, cell adhesion, and cytokine-cytokine receptor interaction (Figure 5D). These analyses suggested that *CD161* regulated immune response to suppress BC progression and improve its prognosis.

### Relationship between *CD161* expression and TILs

**FIGURE 6:**
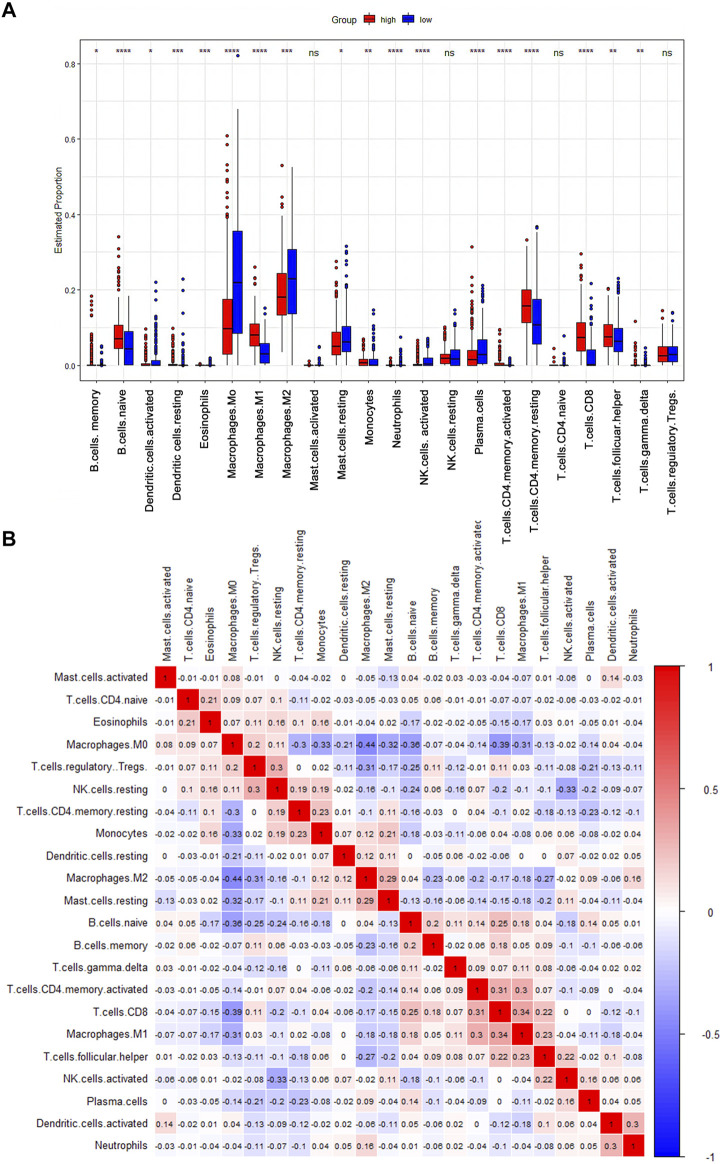
CIBERSORT analysis to evaluate the correlation between *CD161* expression and the immune infiltration in BC. **(A)** The Boxplot showed the ratio differentiation of 22 kinds of immune cells between BC samples with high or low *CD161* expression (red: high *CD161* expression cohort; blue: low *CD161* expression cohort). *p < 0.05, **p < 0.01, ***p < 0.001. **(B)** The correlation matrix showed the relevance between different TILs proportions in BC. The correlation coefficients were exhibited on the colored squares (red: positive Spearman’s rho; blue: negative Spearman’s rho). TILs, tumor infiltrating lymphocytes.

**FIGURE 7:**
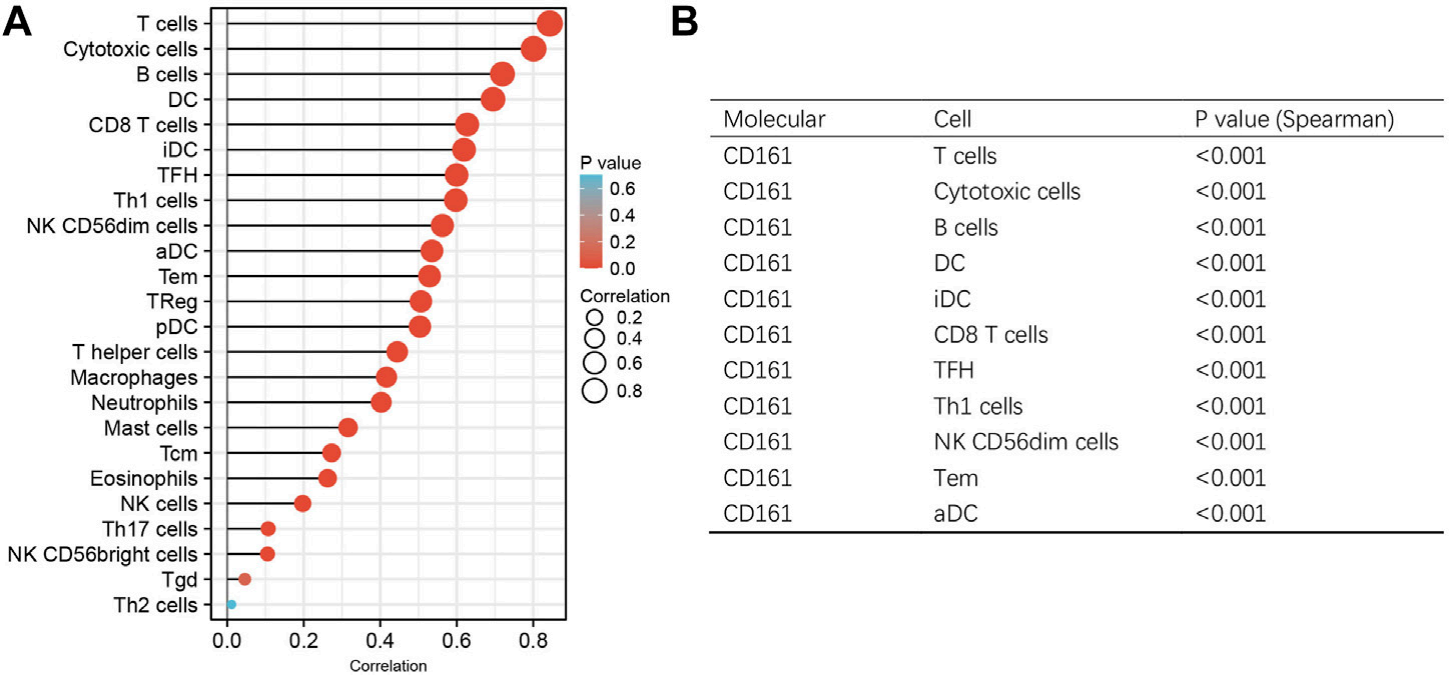
ssGSEA analysis to evaluate the correlation between *CD161* expression and the immune infiltration in BC. **(A)** The Lollipop chart showed the association between *CD161* expression and 24 kinds of immune cells. *X*-axis: Spearman’s rho. *Y*-axis: 24 types of immune cells. The color gradient of the lollipop referred to the correlation *p*-value, and the size of the lollipop referred to the correlation strength. **(B)** The Spearman’s correlation >0.5 and *p* < 0.05 groups were exhibited in the trilinear chart.

**FIGURE 8:**
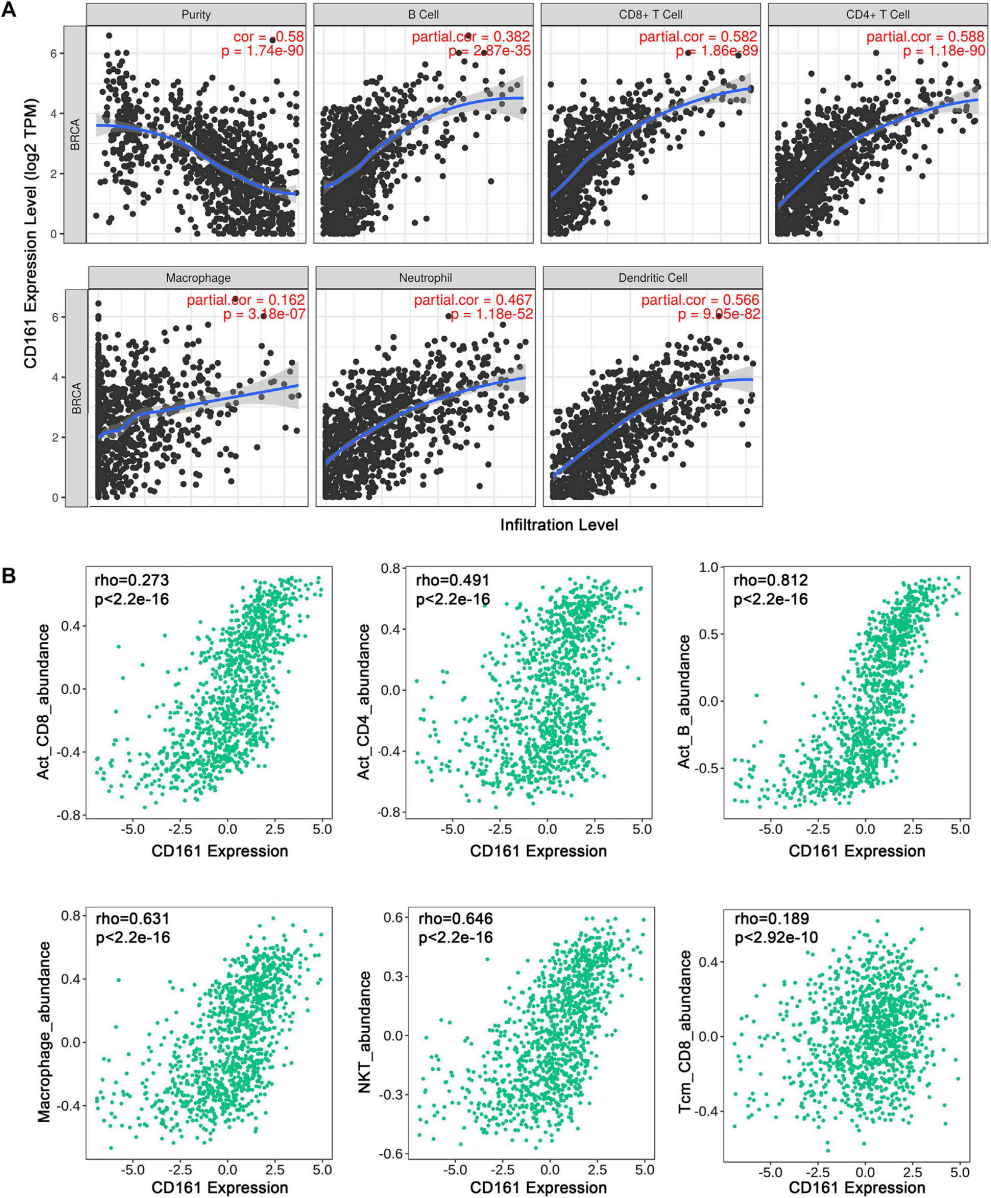
Correlation between *CD161* expression and tumor purity or immune cell abundance in BC through different databases. **(A)** Scatter plots exhibited the correlations between *CD161* expression and tumor purity, B cell, CD8^+^T cell, CD4^+^T cell, Macrophage, Neutrophil and Dendritic cell abundance from TIMER database. **(B)** Scatter plots exhibited the correlations between *CD161* expression and Activated CD8^+^T cell, Activated CD4^+^T cell, Activated B cell, Macrophage, NKT cell and CD8^+^ Tcm abundance from TISIDB database.

As an indispensable element of immune response, TILs are an independent predictor for cancer survival. Therefore, we further explored whether *CD161* expression is related to immune infiltration in BC. CIBERSORT was employed to infer the differences in the proportions of 22 immune cells between high *CD161* and low *CD161* expression groups (Figure 6). The proportions of naive B cells, M1 macrophages, CD8^+^ T cells, CD4^+^ memory resting T cells, and follicular helper T (Tfh) cells were upregulated in the high *CD161* expression group. On the contrary, the proportions of resting dendritic cells, M0 macrophages, neutrophils, activated natural killing (NK) cells, and CD4^+^ memory activated T cells were upregulated in the low *CD161* expression group (Figure 6A). The correlation heatmap exhibited various degrees of correlations within the proportions of different TILs in BC (Figure 6B). The ssGSEA, TIMER database and TISIDB database were next employed to explore the correlation between *CD161* expression level and immune cell infiltration level in BC immune microenvironment. The ssGSEA method showed that *CD161* expression level had a strong correlation with the abundance of T cells (*r* = 0.848, *p* < 0.001), cytotoxic cells (*r* = 0.796, *p* < 0.001), B cells (*r* = 0.719, *p* < 0.001) (Figure 7). TIMER database illustrated a positive association between *CD161* expression level and immune infiltration levels of B cells, CD8^+^ T cells, CD4^+^ T cells, neutrophils, and Dendritic cells (Figure 8A). TISIDB database exhibited that *CD161* expression level was positively correlated with the levels of activated CD8^+^ T cells, activated CD4^+^T cells, activated B cells, macrophages, and NKT cells (Figure 8B). We further employed TIMER and GEPIA databases to explore the relationship between *CD161* expression and the levels of gene markers of immune cells and T cell exhaustion (Table 3 and Supplementary Table S5). The results showed that *CD161* expression was positively correlated with *CD8A* and *CD8B* of CD8^+^ T cell, *CD4* of CD4^+^ T cell, *KIR2DL3* and *KIR3DL2* of NK cell, *CD19* and *CD79A* of B cell, *PD-1* and *CTLA-4* of T cell exhaustion.

**TABLE 3:** Correlation analysis between *CD161* and gene markers of immune cells by TIMER.

Immune cells	Gene markers	None		Purity	
		Correlation	*p* Values	Correlation	*p* Values
T cell	CD3D	0.865	0	0.795	1.40E−217
	CD3E	0.881	0	0.818	3.79E−240
	CD2	0.843	1.35E−297	0.770	6.75E−196
CD4^+^ T cell	CD4	0.683	1.09E−151	0.559	6.66E−83
CD8^+^ T cell	CD8A	0.822	3.39E−270	0.743	1.76E−175
	CD8B	0.743	4.42E−194	0.641	3.95E−116
Th1	IFNG	0.611	1.03E−113	0.511	3.02E−67
	TBX21	0.812	3.44E−259	0.722	4.35E−161
	TNF	0.233	4.68E−15	0.163	2.22E−07
	STAT4	0.779	5.00E−225	0.675	5.16E−113
	STAT1	0.332	1.13E−29	0.278	4.22E−19
Th2	STAT6	0.138	4.44E−06	0.087	5.99E−03
	STAT5A	0.369	9.34E−37	0.229	2.74E−13
	IL13	0.236	2.38E−15	0.169	7.74E−08
Tfh	CXCR5	0.734	1.09E−186	0.621	4.39E−107
	CXCL13	0.569	2.59E−95	0.498	1.84E−63
	BCL6	0.094	1.72E−03	0.050	1.17E−01
	IL21	0.421	2.04E−48	0.350	6.15E−30
Th17	IL17A	0.263	7.22E−19	0.182	6.90E−09
	RORC	−0.068	2.44E−02	−0.067	3.44E−02
	IL23A	0.359	9.62E−35	0.257	1.63E−16
	STAT3	0.033	2.79E−01	−0.021	5.10E−01
Treg	FOXP3	0.592	8.41E−105	0.486	4.80E−60
	IKZF2	0.308	1.23E−25	0.224	8.06E−113
	IL10	0.475	4.20E−63	0.345	3.06E−29
	CCR8	0.387	1.51E−40	0.207	4.35E−11
	STAT5B	0.183	1.04E−09	0.133	2.60E−05
APC/DC	HLA-DPA1	0.688	3.99E−155	0.564	1.03E−84
	HLA-DPB1	0.723	1.57E−178	0.591	1.29E−94
	HLA-DQA1	0.591	1.05E−104	0.487	5.81E−58
B cell	BLK	0.731	1.87E−184	0.617	2.99E−105
	CD19	0.703	2.11E−164	0.584	7.66E−92
	MS4A1	0.790	5.74E−236	0.699	1.84E−146
	CD79A	0.732	5.45E−185	0.608	1.87E−101
Monocyte	CD86	0.532	2.02E−81	0.403	3.39E−40
	CD115/CSF1R	0.509	1.42E−73	0.342	1.13E−28
TAM	CCL2	0.492	5.69E−68	0.344	6.17E−29
M1	INOS/NOS2	0.030	3.21E−01	0.003	9.14E−01
	IRF5	0.277	7.04E−21	0.182	6.85E−09
M2	CD163	0.418	7.37E−48	0.295	2.14E−21
Neutrophils	CD66B/CEACAM8	0.029	3.33E−01	0.056	7.69E−02
	CD11B/ITGAM	0.388	9.10E−41	0.249	1.46E−15
	CCR7	0.816	8.98E−264	0.734	6.31E−169
Natural killer cell	KIR2DL1	0.387	1.20E−40	0.308	2.50E−23
	KIR2DL3	0.412	3.00E−46	0.331	6.72E−27
	KIR3DL1	0.457	9.18E−58	0.356	4.08E−31
	KIR3DL2	0.536	5.35E−83	0.466	9.51E−50
T cell exhaustion	PD-1(PDCD1)	0.731	2.18E−184	0.624	3.30E−108
	CTLA-4	0.648	6.66E−132	0.541	1.74E−76
	LAG3	0.457	8.79E−58	0.362	3.18E−32
	TIM-3(HAVCR2)	0.461	5.96E−59	0.329	1.46E−26
	GZMB	0.672	2.56E−145	0.564	1.97E−84

## Discussion

Due to the heterogeneity of breast tumor, the current clinicopathological staging and typing systems that provide clinical decision support and assist survival outcome prediction still have some limitations (Barry et al., 2010; Zardavas et al., 2015; Liu et al., 2022). New therapeutic strategies and prognostic biomarkers derived from intrinsic characteristics of breast tumor are urgently needed. Plenty of reports have described that breast tumor tissue consists of complex immune contexture (Korkaya et al., 2011; Koelwyn et al., 2017). Through a continuously dynamic interactions, the elements of TME especially tumor-infiltrating immune cells on one hand enhance antitumor immunity by destroying immunogenic tumor variants, and on the other hand promote tumor progression by shaping tumor immunogenicity (Baxevanis et al., 2021). Therefore, identifying the markers regulating tumor immune microenvironment is curcial for facilitating antitumor immunity and improving prognositic outcomes in BC patients. Natural killer cell receptors are found expressed on the surface of NK cells and T cells, participating in the regulation of activating/inhibitory signals and immune response (Zhou et al., 2021; Braud et al., 2022). CD161, encoded by *KLRB1*, is a C-type lectin-related type II transmembrane protein, which belongs to the natural killer cell receptors (Konduri et al., 2020; Braud et al., 2022). Among NK cells, CD161 acts as an inhibitory receptor to inhibit cytotoxicity and cytokine secretion (Richter et al., 2010; López-Soto et al., 2017). Among T cells, CD161 acts as a costimulatory receptor to increase the response to TCR stimulation (Halkias et al., 2019). Previous research has illustrated that high *CD161* expression was associated with favorable clinical outcomes across 39 malignancies, including BC, non-small cell lung cancer, prostate adenocarcinoma, cholangiocarcinoma, and mesothelioma *etc*. (Braud et al., 2018; Zhou et al., 2021; Duurland et al., 2022). Although *CD161* has been recognized as a protective factor for BC patients, the clinical significance and detailed mechanisms of abnormal expression of *CD161* in BC have not been systematically discussed before. In our study, we found that compared with adjacent normal tissues, the expression level of *CD161* was significantly decreased in BC tissues. The lower *CD161* expression was associated with unfavorable clinicopathological features, including higher T stage, higher pathological stage, and distant metastasis. In addition, upregulated *CD161* expression was closely correlated with prolonged OS, DFS and DSS, which is consistent with previous study results (Gentles et al., 2015; Zhou et al., 2021). By Cox regression analysis, we further discovered that *CD161* expression was an independent prognostic factor for OS in BC. Next, we constructed a nomogram with *CD161* expression, PR status and age to predict the OS of BC. The calibration plots and C-index (0.78) implied its outstanding predictive and discriminative power. These results demonstrate that *CD161* can serve as a favorable prognostic biomarker for BC. In diffuse glioma and hepatocellular carcinoma, *CD161* exerts carcinogenic effects by multiple cancer-related signaling pathways, including CD161-CLEC2D pathway, TCR signaling pathway and cytokine-cytokine receptor interaction (Author Anonymous, 2021; Mathewson et al., 2021; Sun et al., 2021). Nevertheless, the function, signaling pathway, and mechanism of *CD161* in BC have not been fully elucidated and deserve further exploration. In our study, the GSEA analysis showed that NK cell-mediated cytotoxicity pathway, TCR signaling pathway, BCR signaling pathway were most significantly enriched in *CD161* low expression group. The KEGG analysis showed that *CD161* co-expressed genes were enriched in pathways of hematopoietic cell lineage, cell adhesion, and cytokine-cytokine receptor interaction. These findings suggest that *CD161* may affect the survival outcomes of BC patients by regulating cancer-related immune response. This theoretical hypothesis requires further experimental validation. TILs are indispensable to an intact immune response to cancer, and their prognostic value has been verified in many solid tumors (Pagès et al., 2005; Maibach et al., 2020; Lopez de Rodas et al., 2022). Previous study has identified that the expression and regulation of *CD161* define CD4^+^ T cells, thus improving the prognosis of OPSCC (Duurland et al., 2022). Accordingly, we next evaluated the correlation between *CD161* expression and immune infiltration. The results indicated a remarkably positive association between *CD161* expression and immune infiltration of B cells, CD8^+^ T cells, CD4^+^ T cells, and NK cells. Moreover, *CD161* expression was also positively correlated with the levels of immune markers of NK cells, T cell, B cells, and T cell exhaustion, further validating the relevance between *CD161* expression and immune infiltration. Based on the results of survival analysis, functional enrichment analysis and immune infiltration analysis, we speculated that *CD161* may regulate immune infiltration to inhibit tumor progression and improve prognosis. We should realize several limitations of this study. First, it is a retrospective analysis based on existing public databases, and some important clinical information is not available. Selection bias and missing data may contribute to inaccuracy of results. Besides, the *in vitro* and *in vivo* experiments were not carried out to confirm the results. Last, the enrichment analysis was not enough to figure out the specific mechanisms involving *CD161*-related immune signaling pathways. The mechanism of *CD161* in regulating the immune cell infiltration in BC should be further explored.

## Conclusion

*CD161* is an independent prognostic factor in BC, and a high expression of *CD161* is significantly correlated with favorable clinicopathological parameters, better clinical outcomes and increased immune infiltration. *CD161* may serve as a potential prognostic and therapeutic biomarker for BC.

## Data Availability

The original contributions presented in the study are included in the article/Supplementary Material, further inquiries can be directed to the corresponding author.

## Abbreviations

BC, breast cancer; BLCA, bladder cancer; BRCA, breast invasive carcinoma; COAD, colon adenocarcinoma; DFS, disease-free survival; DSS, disease-specific survival; GEO, Gene Expression Omnibus; GO, Gene Ontology; GSEA, gene set enrichment analysis; HR, hazard ratio; KLRB1, killer cell lectin-like receptor B1; NK cells, natural killing cells; OPSCC, oropharyngeal squamous cell cancer; OR, odds ratio; OS, overall survival; qRT-PCR, quantitative real-time polymerase chain reaction; TCR, T cell receptor; Tfh, follicular helper T; TILs, tumor-infiltrating lymphocytes; TME, tumor microenvironment.
