# Supplementary material for: Identification of CD161 expression as a novel prognostic biomarker in breast cancer correlated with immune infiltration
Source: Front Genet. 2022 Sep 30;13:996345. doi: 10.3389/fgene.2022.996345 (PMC9561259; doi:10.3389/fgene.2022.996345)
Supplement: Supplementary file 1 [file Table1.doc]

| **Table S1. Clinicopathological information of 1097 patients screened from TCGA database** | | | | | | | | | | | | | |
| --- | --- | --- | --- | --- | --- | --- | --- | --- | --- | --- | --- | --- | --- |
| patient ID | status | time | age | stage | m | n | t | CD161 | menopause | ER | | PR | HER2 |
| 1 | live | 3767 | 55 | NA | NA | NA | NA | 22 | pre | positive | positive | | NA |
| 2 | live | 3801 | 50 | 2 | 0 | 1 | 2 | 63 | post | positive | positive | | NA |
| 3 | live | 1228 | 62 | 2 | 0 | 1 | 2 | 79 | post | positive | positive | | NA |
| 4 | live | 1217 | 52 | 1 | 0 | 0 | 1 | 79 | NA | positive | positive | | NA |
| 5 | live | 158 | 50 | 3 | 0 | 2 | 2 | 55 | post | positive | positive | | NA |
| 6 | live | 1477 | 42 | 2 | 0 | 0 | 2 | 139 | post | positive | positive | | negative |
| 7 | live | 1471 | 63 | 4 | 1 | 0 | 2 | 97 | post | positive | positive | | NA |
| 8 | live | 12 | 52 | 2 | NA | NA | 2 | 6 | NA | positive | negative | | negative |
| 9 | live | 259 | 70 | 1 | 0 | 0 | 1 | 13 | post | positive | negative | | NA |
| 10 | live | 437 | 59 | 2 | 0 | 0 | 2 | 109 | NA | positive | positive | | NA |
| 11 | live | 1321 | 56 | 1 | 0 | 0 | 1 | 197 | pre | positive | positive | | negative |
| 12 | live | 1463 | 54 | 2 | 0 | 0 | 2 | 371 | pre | positive | positive | | NA |
| 13 | live | 434 | 61 | 2 | 0 | 1 | 2 | 98 | post | positive | positive | | NA |
| 14 | live | 1437 | 39 | 2 | 0 | 0 | 2 | 65 | pre | negative | positive | | negative |
| 15 | live | 635 | 52 | 2 | 0 | 1 | 2 | 100 | NA | positive | positive | | NA |
| 16 | live | 416 | 39 | 3 | 0 | 1 | 3 | 201 | NA | positive | positive | | negative |
| 17 | dead | 967 | 54 | 2 | 0 | 0 | 2 | 0 | NA | negative | negative | | negative |
| 18 | live | 242 | 77 | 2 | 0 | 0 | 2 | 29 | NA | positive | negative | | positive |
| 19 | live | 1196 | 50 | 2 | NA | 1 | 1 | 209 | post | positive | positive | | NA |
| 20 | live | 852 | 67 | 2 | 0 | 1 | 2 | 26 | post | negative | negative | | negative |
| 21 | live | 584 | 40 | 2 | 0 | 0 | 2 | 155 | NA | negative | negative | | negative |
| 22 | live | 554 | 45 | 2 | NA | 1 | 2 | 47 | pre | positive | positive | | NA |
| 23 | live | 3153 | 66 | 1 | 0 | 0 | 1 | 45 | post | positive | positive | | negative |
| 24 | dead | 548 | 36 | 3 | 0 | 3 | 2 | 119 | pre | negative | negative | | negative |
| 25 | live | 2179 | 48 | 1 | 0 | 0 | 1 | 895 | post | negative | negative | | negative |
| 26 | live | 2365 | 36 | 1 | 0 | 0 | 1 | 15 | pre | positive | positive | | negative |
| 27 | live | 1950 | 62 | 2 | 0 | 0 | 2 | 82 | post | negative | negative | | negative |
| 28 | live | 671 | 47 | 2 | 0 | 0 | 2 | 5 | post | negative | negative | | negative |
| 29 | dead | 1920 | 39 | 2 | 0 | 0 | 2 | 87 | pre | positive | positive | | negative |
| 30 | live | 1918 | 50 | 2 | 0 | 1 | 2 | 80 | pre | negative | negative | | positive |
| 31 | live | 1349 | 34 | 2 | 0 | 0 | 2 | 217 | pre | positive | positive | | positive |
| 32 | live | 764 | 53 | 2 | 0 | 1 | 2 | 147 | post | positive | positive | | negative |
| 33 | live | 3820 | 60 | 3 | 0 | 2 | 3 | 194 | post | positive | positive | | negative |
| 34 | live | 1827 | 37 | 3 | 0 | 2 | 3 | 432 | pre | positive | positive | | negative |
| 35 | dead | 754 | 40 | 2 | 0 | 0 | 2 | 47 | pre | negative | negative | | negative |
| 36 | live | 3409 | 85 | 2 | 0 | 0 | 3 | 155 | post | positive | positive | | negative |
| 37 | live | 2495 | 60 | 1 | 0 | 0 | 1 | 54 | post | positive | positive | | negative |
| 38 | live | 2393 | 62 | 1 | 0 | 0 | 1 | 47 | post | positive | positive | | negative |
| 39 | live | 2955 | 54 | 2 | 0 | 0 | 3 | 912 | post | positive | positive | | negative |
| 40 | live | 2298 | 73 | 4 | 1 | 3 | 4 | 10 | post | positive | positive | | negative |
| 41 | live | 1918 | 71 | 2 | 0 | 0 | 2 | 37 | post | positive | negative | | negative |
| 42 | dead | 158 | 73 | 2 | 0 | 0 | 2 | 40 | post | positive | positive | | negative |
| 43 | live | 1870 | 41 | 2 | 0 | 1 | 2 | 381 | post | positive | positive | | negative |
| 44 | live | 1750 | 67 | 2 | 0 | 1 | 2 | 26 | post | positive | positive | | negative |
| 45 | live | 1303 | 52 | 2 | 0 | 0 | 2 | 68 | pre | positive | negative | | positive |
| 46 | live | 1288 | 63 | 2 | 0 | 1 | 2 | 2 | post | positive | positive | | negative |
| 47 | live | 1339 | 46 | 2 | 0 | 0 | 2 | 185 | post | positive | positive | | negative |
| 48 | live | 643 | 60 | 2 | 0 | 0 | 2 | 64 | post | negative | negative | | negative |
| 49 | live | 786 | 76 | 2 | 0 | 0 | 2 | 5 | post | negative | negative | | positive |
| 50 | live | 761 | 45 | 2 | 0 | 0 | 2 | 97 | pre | negative | negative | | negative |
| 51 | live | 736 | 42 | 1 | 0 | 0 | 1 | 85 | post | positive | positive | | negative |
| 52 | live | 496 | 37 | 2 | 0 | 1 | 2 | 35 | pre | positive | positive | | negative |
| 53 | live | 2758 | 73 | 1 | 0 | 0 | 1 | 20 | post | positive | positive | | negative |
| 54 | live | 4088 | 70 | 2 | 0 | 0 | 2 | 322 | post | positive | positive | | negative |
| 55 | live | 2181 | 54 | 1 | 0 | 0 | 1 | 209 | post | positive | positive | | negative |
| 56 | live | 3021 | 56 | 1 | 0 | 0 | 1 | 1088 | post | positive | negative | | negative |
| 57 | live | 2053 | 64 | 2 | 0 | 0 | 2 | 397 | post | negative | negative | | positive |
| 58 | live | 1903 | 63 | 1 | 0 | 1 | 1 | 119 | post | positive | positive | | negative |
| 59 | live | 1001 | 52 | 2 | 0 | 0 | 2 | 145 | post | positive | positive | | negative |
| 60 | live | 807 | 58 | 3 | 0 | 2 | 2 | 54 | post | positive | positive | | negative |
| 61 | live | 733 | 79 | 1 | 0 | 0 | 1 | 18 | post | positive | positive | | negative |
| 62 | live | 545 | 80 | 1 | 0 | 0 | 1 | 105 | post | positive | positive | | negative |
| 63 | live | 770 | 53 | 3 | 0 | 3 | 1 | 170 | post | positive | positive | | negative |
| 64 | live | 550 | 46 | 2 | 0 | 0 | 3 | 228 | post | positive | positive | | negative |
| 65 | live | 485 | 62 | 2 | 0 | 1 | 2 | 205 | post | positive | negative | | positive |
| 66 | live | 2619 | 62 | 2 | 0 | 1 | 1 | 489 | post | negative | negative | | negative |
| 67 | live | 1352 | 66 | 2 | 0 | 0 | 2 | 136 | post | positive | positive | | negative |
| 68 | dead | 825 | 63 | 4 | 1 | 2 | 2 | 36 | post | positive | positive | | negative |
| 69 | dead | 1365 | 82 | 4 | 1 | 2 | 2 | 227 | post | positive | negative | | negative |
| 70 | live | 1288 | 48 | 1 | 0 | 0 | 1 | 237 | pre | negative | negative | | negative |
| 71 | live | 1154 | 62 | 3 | 0 | 1 | 3 | 306 | post | positive | positive | | positive |
| 72 | live | 337 | 59 | 2 | 0 | 1 | 2 | 149 | post | negative | negative | | negative |
| 73 | live | 323 | 55 | 3 | 0 | 3 | 3 | 405 | post | negative | negative | | positive |
| 74 | dead | 255 | 66 | 4 | 1 | 3 | 3 | 21 | post | negative | negative | | negative |
| 75 | live | 456 | 37 | 1 | 0 | 1 | 1 | 82 | pre | positive | positive | | negative |
| 76 | live | 395 | 62 | 2 | 0 | 0 | 2 | 228 | post | positive | positive | | negative |
| 77 | live | 321 | 39 | 2 | 0 | 0 | 2 | 41 | pre | positive | positive | | negative |
| 78 | live | 344 | 50 | 2 | 0 | 0 | 3 | 282 | pre | positive | positive | | negative |
| 79 | live | 397 | 51 | 2 | 0 | 0 | 2 | 300 | pre | positive | positive | | negative |
| 80 | live | 644 | 59 | 2 | 0 | 1 | 2 | 46 | post | positive | positive | | negative |
| 81 | live | 550 | 63 | 2 | 0 | 0 | 3 | 104 | post | positive | positive | | negative |
| 82 | live | 253 | 48 | 2 | 0 | 1 | 2 | 41 | post | negative | negative | | negative |
| 83 | live | 289 | 67 | 1 | 0 | 0 | 1 | 71 | post | positive | negative | | negative |
| 84 | live | 404 | 63 | 3 | 0 | 3 | 2 | 32 | post | positive | positive | | positive |
| 85 | live | 379 | 53 | 3 | 0 | 2 | 2 | 83 | post | positive | positive | | negative |
| 86 | live | 358 | 62 | 1 | 0 | 0 | 1 | 344 | post | positive | positive | | negative |
| 87 | live | 326 | 39 | 3 | 0 | 2 | 3 | 5 | pre | positive | negative | | negative |
| 88 | live | 334 | 61 | 2 | 0 | 0 | 2 | 463 | post | positive | negative | | negative |
| 89 | live | 268 | 48 | 3 | 0 | 2 | 3 | 264 | pre | positive | positive | | negative |
| 90 | live | 662 | 67 | 2 | 0 | 0 | 2 | 22 | post | negative | negative | | negative |
| 91 | dead | 723 | 56 | 3 | 0 | 2 | 4 | 39 | post | positive | negative | | negative |
| 92 | live | 461 | 74 | 2 | 0 | 0 | 3 | 53 | post | positive | positive | | negative |
| 93 | live | 221 | 62 | 3 | 0 | 2 | 3 | 77 | post | positive | negative | | NA |
| 94 | live | 935 | 61 | 3 | 0 | 2 | 3 | 63 | post | positive | positive | | negative |
| 95 | live | 410 | 63 | 2 | 0 | 0 | 2 | 97 | post | positive | positive | | NA |
| 96 | live | 380 | 49 | 2 | 0 | 1 | 2 | 234 | pre | positive | positive | | negative |
| 97 | live | 371 | 85 | 2 | 0 | 1 | 2 | 126 | post | negative | negative | | positive |
| 98 | live | 372 | 71 | 3 | 0 | 1 | 3 | 38 | post | positive | positive | | NA |
| 99 | live | 132 | 50 | 3 | 0 | 2 | 2 | 325 | pre | negative | negative | | negative |
| 100 | live | 1286 | 70 | 1 | 0 | 0 | 1 | 189 | post | positive | positive | | negative |
| 101 | live | 2968 | 44 | 2 | 0 | 0 | 2 | 504 | pre | positive | positive | | negative |
| 102 | live | 258 | 39 | 2 | 0 | 1 | 2 | 44 | pre | positive | positive | | negative |
| 103 | live | 285 | 50 | 2 | 0 | 1 | 2 | 191 | pre | positive | positive | | negative |
| 104 | live | 243 | 90 | 3 | 0 | 3 | 2 | 162 | post | positive | negative | | negative |
| 105 | live | 2511 | 34 | 3 | 0 | 2 | 2 | 138 | pre | positive | positive | | NA |
| 106 | live | 114 | 66 | 2 | 0 | 0 | 2 | 2389 | post | negative | positive | | NA |
| 107 | live | 363 | 55 | 2 | 0 | 1 | 2 | 171 | pre | positive | positive | | negative |
| 108 | live | 804 | 47 | 3 | 0 | 1 | 3 | 100 | pre | positive | positive | | negative |
| 109 | dead | 1032 | 62 | 3 | 0 | 2 | 1 | 81 | post | negative | negative | | negative |
| 110 | live | 2238 | 45 | 2 | 0 | 1 | 2 | 166 | pre | negative | negative | | negative |
| 111 | dead | 912 | 35 | 2 | 0 | 1 | 2 | 9 | post | negative | negative | | negative |
| 112 | live | 428 | 46 | 2 | 0 | 0 | 2 | 37 | pre | positive | negative | | positive |
| 113 | live | 1209 | 42 | 2 | 0 | 1 | 2 | 87 | pre | positive | negative | | negative |
| 114 | live | 1168 | 49 | 2 | 0 | 0 | 2 | 66 | post | negative | negative | | negative |
| 115 | live | 786 | 49 | 2 | 0 | 1 | 2 | 165 | post | negative | negative | | negative |
| 116 | live | 973 | 46 | 1 | 0 | 0 | 1 | 638 | pre | negative | negative | | positive |
| 117 | live | 859 | 57 | 2 | 0 | 1 | 2 | 148 | post | positive | negative | | negative |
| 118 | live | 222 | 49 | 3 | 0 | 3 | 2 | 30 | pre | positive | positive | | negative |
| 119 | live | 274 | 67 | 2 | 0 | 0 | 2 | 43 | post | positive | positive | | negative |
| 120 | live | 268 | 46 | 3 | 0 | 3 | 3 | 151 | pre | positive | positive | | negative |
| 121 | live | 271 | 77 | 2 | 0 | 0 | 2 | 4 | post | positive | positive | | negative |
| 122 | live | 365 | 66 | 2 | 0 | 0 | 2 | 63 | post | positive | negative | | NA |
| 123 | live | 205 | 62 | 3 | 0 | 2 | 3 | 42 | post | positive | positive | | negative |
| 124 | live | 104 | 59 | 2 | 0 | 1 | 2 | 47 | post | positive | positive | | NA |
| 125 | live | 171 | 66 | 1 | 0 | 0 | 1 | 5 | NA | positive | positive | | negative |
| 126 | live | 254 | 57 | 2 | 0 | 0 | 2 | 47 | NA | negative | negative | | negative |
| 127 | live | 167 | 78 | 2 | 0 | 0 | 2 | 256 | post | positive | negative | | negative |
| 128 | live | 162 | 79 | 2 | 0 | 0 | 2 | 9 | post | NA | NA | | NA |
| 129 | live | 189 | 57 | 2 | 0 | 0 | 2 | 20 | post | positive | positive | | negative |
| 130 | live | 160 | 37 | 2 | 0 | 0 | 2 | 49 | pre | positive | negative | | negative |
| 131 | live | 373 | 62 | 2 | 0 | 0 | 2 | 9 | post | negative | negative | | negative |
| 132 | live | 141 | 56 | 2 | 0 | 0 | 2 | 57 | post | positive | positive | | negative |
| 133 | live | 273 | 63 | 1 | 0 | 0 | 1 | NA | post | positive | negative | | negative |
| 134 | live | 267 | 46 | 2 | 0 | 0 | 2 | 0 | post | negative | positive | | negative |
| 135 | live | 287 | 62 | 2 | NA | 1 | 2 | 4 | post | positive | negative | | negative |
| 136 | live | 196 | 44 | 3 | 0 | 1 | 3 | 6 | pre | positive | positive | | negative |
| 137 | live | 208 | 79 | 2 | NA | 0 | 2 | 6 | post | positive | positive | | negative |
| 138 | live | 551 | 61 | 2 | NA | 1 | 2 | 138 | post | positive | positive | | negative |
| 139 | live | 421 | 71 | 3 | NA | 1 | 3 | 78 | post | positive | positive | | negative |
| 140 | live | 257 | 55 | 1 | 0 | 0 | 1 | 134 | post | negative | negative | | negative |
| 141 | live | 210 | 50 | 2 | 0 | 0 | 2 | 148 | pre | negative | negative | | NA |
| 142 | live | 65 | 72 | 2 | 0 | 1 | 1 | 57 | post | positive | negative | | positive |
| 143 | live | 122 | 65 | 2 | 0 | 0 | 2 | 16 | post | negative | negative | | negative |
| 144 | live | 68 | 49 | 2 | 0 | 0 | 2 | 52 | pre | positive | positive | | negative |
| 145 | live | 182 | 53 | 3 | NA | 2 | 2 | 91 | post | positive | positive | | positive |
| 146 | live | 151 | 71 | 1 | 0 | 0 | 1 | 52 | post | positive | positive | | NA |
| 147 | live | 148 | 62 | 2 | 0 | 0 | 2 | 6 | post | positive | negative | | negative |
| 148 | live | 76 | 62 | 2 | 0 | 0 | 2 | 58 | post | positive | positive | | negative |
| 149 | live | 75 | 63 | 1 | NA | 0 | 1 | 342 | post | positive | positive | | NA |
| 150 | live | 54 | 79 | 2 | 0 | 0 | 2 | 11 | post | positive | positive | | NA |
| 151 | live | 293 | 70 | 3 | NA | 3 | 3 | 125 | post | positive | positive | | positive |
| 152 | live | 229 | 50 | 3 | NA | 3 | 3 | 397 | pre | positive | positive | | NA |
| 153 | live | 241 | 40 | 2 | 0 | 1 | 2 | 94 | pre | positive | negative | | negative |
| 154 | live | 200 | 56 | 3 | 0 | 1 | 3 | 13 | post | positive | positive | | NA |
| 155 | live | 215 | 62 | 2 | NA | 0 | 3 | 79 | post | positive | negative | | positive |
| 156 | live | 168 | 52 | 2 | 0 | 0 | 2 | 30 | post | negative | negative | | negative |
| 157 | live | 371 | 54 | 2 | 0 | 0 | 2 | 7 | post | negative | negative | | negative |
| 158 | live | 369 | 54 | 2 | 0 | 0 | 2 | 10 | post | positive | negative | | positive |
| 159 | live | 177 | 84 | 2 | 0 | 0 | 2 | 0 | post | positive | positive | | NA |
| 160 | live | 210 | 62 | 2 | 0 | 0 | 2 | 6 | post | negative | negative | | negative |
| 161 | live | 134 | 47 | 2 | 0 | 1 | 1 | 156 | post | positive | positive | | negative |
| 162 | live | 132 | 48 | 3 | 0 | 3 | 2 | 35 | pre | positive | positive | | NA |
| 163 | live | 181 | 51 | 2 | 0 | 0 | 2 | 30 | post | negative | negative | | NA |
| 164 | live | 176 | 48 | 2 | 0 | 0 | 2 | 42 | post | negative | negative | | NA |
| 165 | live | 169 | 68 | 2 | 0 | 0 | 2 | 8 | post | positive | positive | | negative |
| 166 | live | 125 | 48 | 2 | 0 | 1 | 2 | 48 | post | negative | negative | | NA |
| 167 | live | 0 | 66 | 3 | 0 | 0 | 4 | 6 | post | positive | negative | | negative |
| 168 | live | 396 | 60 | 1 | 0 | 0 | 1 | 97 | post | positive | positive | | negative |
| 169 | live | 396 | 63 | 3 | 0 | 2 | 1 | 32 | post | positive | positive | | negative |
| 170 | live | 31 | 63 | 3 | 0 | 2 | 3 | 29 | post | positive | positive | | negative |
| 171 | live | 547 | 69 | 2 | 0 | 1 | 2 | 35 | post | positive | negative | | positive |
| 172 | live | 1249 | 75 | 3 | 0 | 1 | 3 | 108 | post | positive | positive | | positive |
| 173 | dead | 883 | 80 | 2 | 0 | 1 | 2 | 40 | post | positive | positive | | positive |
| 174 | dead | 943 | 77 | 2 | 0 | 0 | 3 | 22 | post | positive | negative | | positive |
| 175 | live | 791 | 66 | 2 | 0 | 0 | 2 | 4 | post | positive | positive | | negative |
| 176 | live | 31 | 84 | 2 | 0 | 0 | 3 | 15 | post | positive | positive | | negative |
| 177 | live | 518 | 42 | 2 | 0 | 1 | 2 | 127 | pre | positive | positive | | positive |
| 178 | live | 1642 | 66 | 2 | 0 | 0 | 2 | 72 | post | positive | positive | | positive |
| 179 | live | 274 | 69 | 3 | 0 | 3 | 4 | 16 | post | positive | positive | | negative |
| 180 | live | 1004 | 69 | 2 | 0 | 0 | 2 | 2 | post | positive | positive | | positive |
| 181 | live | 580 | 57 | 2 | 0 | 0 | 2 | 131 | post | negative | negative | | negative |
| 182 | live | 608 | 81 | NA | NA | 3 | 4 | 193 | post | positive | positive | | negative |
| 183 | live | 577 | 65 | 2 | 0 | 1 | 2 | 135 | post | positive | positive | | negative |
| 184 | live | 577 | 65 | 2 | 0 | 1 | 1 | 144 | post | positive | positive | | negative |
| 185 | live | 426 | 69 | 3 | 0 | 2 | 2 | 44 | post | positive | negative | | positive |
| 186 | live | 365 | 35 | 2 | 0 | 1 | 2 | 96 | pre | positive | positive | | negative |
| 187 | live | 518 | 58 | 3 | 0 | 1 | 3 | 19 | post | positive | positive | | negative |
| 188 | live | 304 | 51 | 2 | 0 | 0 | 2 | 41 | post | negative | negative | | negative |
| 189 | live | 334 | 68 | 2 | 0 | 1 | 2 | 67 | post | positive | positive | | positive |
| 190 | live | 273 | 80 | 3 | 0 | 3 | 2 | 1 | NA | negative | negative | | NA |
| 191 | live | 243 | 73 | 2 | 0 | 0 | 2 | 6 | post | positive | negative | | negative |
| 192 | live | 303 | 66 | 3 | 0 | 2 | 2 | 151 | post | negative | positive | | negative |
| 193 | live | 304 | 76 | 4 | 1 | NA | 2 | 30 | post | positive | positive | | negative |
| 194 | live | 853 | 85 | 2 | 0 | 0 | 2 | 42 | post | positive | positive | | negative |
| 195 | live | 0 | 80 | 2 | 0 | 0 | 2 | 309 | post | positive | positive | | negative |
| 196 | live | 549 | 58 | 2 | 0 | 1 | 2 | 2 | post | positive | positive | | negative |
| 197 | live | 0 | 67 | 2 | 0 | 0 | 3 | 17 | post | positive | positive | | negative |
| 198 | live | 458 | 81 | 2 | 0 | 1 | 2 | 0 | post | positive | negative | | negative |
| 199 | live | 1124 | 44 | 2 | 0 | 1 | 2 | 1 | NA | positive | positive | | negative |
| 200 | live | 396 | 59 | 2 | 0 | 1 | 1 | 1 | post | positive | positive | | negative |
| 201 | live | 30 | 89 | 1 | 0 | 0 | 1 | 73 | post | positive | positive | | negative |
| 202 | live | 702 | 52 | 2 | 0 | 0 | 2 | 7 | pre | positive | negative | | positive |
| 203 | live | 608 | 65 | 2 | 0 | 0 | 2 | 31 | post | positive | positive | | positive |
| 204 | live | 549 | 59 | 3 | 0 | 3 | 2 | 46 | post | positive | positive | | negative |
| 205 | live | 607 | 41 | 2 | 0 | 0 | 2 | 26 | pre | positive | positive | | positive |
| 206 | live | 0 | 66 | 2 | 0 | 0 | 2 | 241 | post | positive | positive | | positive |
| 207 | live | 365 | 53 | 2 | 0 | 0 | 2 | 359 | post | positive | positive | | negative |
| 208 | dead | 1127 | 52 | 4 | 1 | 3 | 4 | 23 | post | positive | negative | | negative |
| 209 | dead | 304 | 89 | 3 | 0 | 2 | 3 | 169 | post | positive | negative | | negative |
| 210 | live | 943 | 45 | 4 | 1 | 3 | 2 | 24 | pre | positive | positive | | negative |
| 211 | live | 943 | 70 | 3 | 0 | 2 | 2 | 153 | post | positive | positive | | positive |
| 212 | live | 30 | 52 | 2 | 0 | 1 | 2 | 102 | post | negative | negative | | negative |
| 213 | live | 580 | 71 | 2 | 0 | 1 | 1 | 7 | post | positive | positive | | positive |
| 214 | live | 2830 | 64 | 4 | 1 | 1 | 2 | 83 | post | positive | positive | | positive |
| 215 | live | 1034 | 43 | 3 | 0 | 3 | 4 | 93 | pre | negative | negative | | positive |
| 216 | live | 1217 | 76 | NA | 0 | 3 | 4 | 106 | post | positive | positive | | negative |
| 217 | live | 0 | 74 | 2 | 0 | 0 | 2 | 49 | post | positive | positive | | positive |
| 218 | live | 580 | 61 | 2 | 0 | 0 | 2 | 67 | post | positive | negative | | negative |
| 219 | live | 487 | 48 | 3 | 0 | 2 | 2 | 7 | pre | positive | positive | | negative |
| 220 | live | 546 | 61 | 2 | 0 | 0 | 2 | 16 | post | positive | positive | | negative |
| 221 | live | 0 | 75 | 2 | 0 | 0 | 2 | 57 | post | positive | negative | | negative |
| 222 | live | 1277 | 45 | 1 | 0 | 0 | 1 | 151 | pre | positive | positive | | negative |
| 223 | live | 0 | 73 | 2 | 0 | 0 | 2 | 171 | post | positive | positive | | negative |
| 224 | live | 365 | 65 | 2 | 0 | 1 | 2 | 67 | post | positive | positive | | positive |
| 225 | live | 304 | 76 | NA | NA | 3 | 4 | 82 | post | positive | positive | | positive |
| 226 | live | 304 | 40 | NA | 0 | NA | 2 | 101 | pre | positive | positive | | negative |
| 227 | live | 365 | 58 | 3 | 0 | 1 | 4 | 26 | post | positive | positive | | negative |
| 228 | live | 31 | 69 | NA | NA | 0 | 2 | 33 | post | positive | positive | | negative |
| 229 | live | 396 | 47 | 2 | 0 | 1 | 2 | 120 | post | positive | positive | | negative |
| 230 | live | 943 | 73 | 3 | 0 | 1 | 4 | 14 | post | positive | positive | | positive |
| 231 | live | 0 | 79 | 3 | 0 | 3 | 3 | 187 | post | positive | negative | | positive |
| 232 | live | 1006 | 84 | 2 | 0 | 0 | 2 | 33 | post | positive | positive | | positive |
| 233 | live | 912 | 68 | 2 | 0 | 1 | 1 | 124 | post | positive | positive | | negative |
| 234 | live | 457 | 75 | 3 | 0 | 3 | 2 | 152 | post | positive | positive | | negative |
| 235 | live | 31 | 57 | 3 | 0 | 3 | 2 | 39 | post | positive | positive | | positive |
| 236 | live | 761 | 83 | 3 | 0 | 2 | 4 | 270 | post | positive | positive | | negative |
| 237 | live | 273 | 82 | 2 | 0 | 1 | 2 | 68 | post | positive | positive | | negative |
| 238 | live | 579 | 68 | NA | NA | 0 | 1 | 3 | post | positive | positive | | negative |
| 239 | live | 457 | 51 | 2 | 0 | 0 | 2 | 6 | pre | positive | positive | | negative |
| 240 | live | 30 | 70 | 3 | 0 | 3 | 2 | 46 | post | positive | positive | | negative |
| 241 | dead | 426 | 62 | 3 | 0 | 3 | 2 | 149 | post | negative | negative | | negative |
| 242 | live | 0 | 83 | 2 | 0 | 0 | 3 | 114 | post | positive | negative | | negative |
| 243 | live | 365 | 84 | 2 | 0 | 0 | 2 | 77 | post | positive | positive | | negative |
| 244 | live | 579 | 66 | 2 | 0 | 0 | 2 | 61 | post | positive | positive | | negative |
| 245 | live | 396 | 73 | 2 | 0 | 0 | 2 | 42 | post | positive | positive | | negative |
| 246 | live | 640 | 64 | 3 | 0 | 3 | 2 | 362 | post | positive | positive | | negative |
| 247 | live | 30 | 57 | 2 | 0 | 1 | 2 | 1070 | post | negative | negative | | positive |
| 248 | live | 396 | 80 | 2 | 0 | 0 | 2 | 44 | post | positive | positive | | negative |
| 249 | live | 518 | 54 | 2 | 0 | 0 | 2 | 25 | post | positive | positive | | positive |
| 250 | live | 1157 | 83 | 1 | 0 | 0 | 1 | 12 | post | positive | positive | | negative |
| 251 | live | 29 | 62 | 2 | NA | 1 | 2 | 128 | post | positive | positive | | positive |
| 252 | live | 72 | 50 | 2 | NA | 1 | 2 | 49 | NA | positive | positive | | NA |
| 253 | live | 330 | 76 | 2 | NA | 1 | 1 | 33 | post | positive | positive | | positive |
| 254 | live | 81 | 90 | 2 | 0 | NA | 2 | 12 | post | positive | negative | | positive |
| 255 | live | 45 | 84 | 2 | 0 | 0 | 3 | 276 | post | positive | positive | | negative |
| 256 | live | 1172 | 78 | 3 | NA | 2 | 2 | 5 | post | negative | negative | | NA |
| 257 | live | 1162 | 41 | 2 | NA | 1 | 2 | 166 | NA | positive | positive | | negative |
| 258 | live | 44 | 65 | 2 | NA | 0 | 2 | 491 | NA | positive | positive | | positive |
| 259 | live | 791 | 62 | 3 | NA | 3 | 3 | 234 | post | positive | positive | | NA |
| 260 | live | 1686 | 40 | 2 | NA | 1 | 2 | 403 | NA | positive | positive | | negative |
| 261 | live | 1125 | 79 | 2 | NA | 1 | 2 | 162 | post | positive | negative | | negative |
| 262 | live | 1180 | 45 | 3 | 0 | 3 | 2 | 198 | NA | positive | positive | | negative |
| 263 | dead | 792 | 87 | 2 | 0 | 1 | 2 | 10 | post | NA | NA | | NA |
| 264 | live | 1542 | 65 | 2 | NA | 1 | 2 | 483 | post | positive | negative | | NA |
| 265 | live | 31 | 58 | 2 | NA | 0 | 3 | 3 | post | negative | negative | | NA |
| 266 | live | 28 | 76 | 3 | NA | 1 | 3 | 97 | post | positive | negative | | negative |
| 267 | live | 69 | 48 | 3 | 0 | 0 | 4 | 92 | post | negative | negative | | NA |
| 268 | live | 30 | 46 | 3 | NA | 2 | 3 | 389 | NA | positive | positive | | NA |
| 269 | live | 17 | 79 | 3 | 0 | 3 | 3 | 11 | post | positive | negative | | negative |
| 270 | live | 21 | 87 | 2 | NA | 1 | 2 | 142 | post | positive | positive | | NA |
| 271 | live | 35 | 68 | 2 | NA | 1 | 2 | 493 | post | positive | positive | | negative |
| 272 | live | 286 | 79 | 2 | NA | 1 | 2 | 54 | post | positive | positive | | negative |
| 273 | live | 34 | 54 | 1 | NA | 0 | 1 | 116 | post | positive | positive | | positive |
| 274 | live | 93 | 50 | 3 | 0 | 1 | 3 | 146 | post | positive | positive | | NA |
| 275 | live | 34 | 75 | 2 | NA | 0 | 3 | 27 | post | positive | positive | | NA |
| 276 | live | 55 | 65 | 2 | NA | 0 | 2 | 135 | NA | positive | positive | | positive |
| 277 | live | 51 | 90 | 3 | NA | 1 | 3 | 190 | post | positive | positive | | positive |
| 278 | live | 192 | 66 | 2 | NA | 1 | 2 | 28 | post | positive | negative | | NA |
| 279 | live | 47 | 74 | 3 | NA | 3 | 1 | 56 | post | positive | positive | | negative |
| 280 | live | 40 | 66 | 2 | NA | 0 | 2 | 36 | post | positive | positive | | NA |
| 281 | live | 152 | 63 | 2 | 0 | 0 | 3 | 74 | post | positive | positive | | NA |
| 282 | live | 63 | 76 | 2 | NA | 1 | 2 | 52 | NA | positive | negative | | NA |
| 283 | live | 0 | 88 | 1 | NA | 1 | 1 | NA | NA | positive | positive | | NA |
| 284 | live | 47 | 74 | 2 | NA | 0 | 2 | 155 | NA | positive | positive | | NA |
| 285 | live | 9 | 74 | 2 | NA | 0 | 3 | 33 | NA | positive | positive | | NA |
| 286 | live | 2 | 58 | 4 | 1 | 1 | 2 | 42 | NA | positive | positive | | NA |
| 287 | live | 34 | 72 | 2 | NA | 0 | 2 | 4 | post | positive | negative | | NA |
| 288 | live | 55 | 79 | 2 | NA | 1 | 2 | 54 | post | positive | positive | | negative |
| 289 | live | 36 | 47 | 2 | NA | 1 | 2 | 380 | NA | positive | positive | | NA |
| 290 | live | 21 | 73 | 2 | NA | 0 | 2 | 176 | post | negative | negative | | NA |
| 291 | live | 13 | 49 | 3 | NA | 3 | 2 | 68 | post | positive | positive | | NA |
| 292 | live | 51 | 43 | 2 | NA | 1 | 2 | 51 | NA | positive | positive | | NA |
| 293 | live | 74 | 51 | 2 | NA | 1 | 1 | 5 | NA | positive | negative | | NA |
| 294 | live | 1 | 56 | 2 | NA | 0 | 3 | 104 | post | negative | negative | | NA |
| 295 | live | 53 | 72 | 1 | NA | 0 | 1 | 70 | post | positive | positive | | negative |
| 296 | live | 34 | 72 | 2 | NA | 1 | 2 | 82 | post | negative | negative | | NA |
| 297 | live | 40 | 75 | 1 | NA | 0 | 1 | 3 | post | positive | positive | | NA |
| 298 | live | 70 | 71 | 2 | NA | 0 | 2 | 85 | post | positive | positive | | negative |
| 299 | live | 10 | 74 | 2 | 0 | 0 | 2 | 30 | post | positive | positive | | negative |
| 300 | live | 10 | 66 | 2 | 0 | 0 | 2 | 56 | post | positive | positive | | negative |
| 301 | live | 7 | 29 | 2 | 0 | 1 | 2 | 56 | pre | positive | negative | | NA |
| 302 | live | 10 | 68 | 2 | 0 | 0 | 2 | 115 | post | positive | positive | | NA |
| 303 | live | 19 | 62 | 2 | 0 | 0 | 2 | 77 | post | positive | positive | | negative |
| 304 | live | 90 | 36 | 3 | 0 | 2 | 2 | 112 | post | positive | positive | | negative |
| 305 | live | 54 | 51 | 2 | 0 | 1 | 2 | 126 | post | negative | negative | | positive |
| 306 | live | 52 | 58 | 2 | 0 | 1 | 2 | 10 | post | negative | negative | | negative |
| 307 | live | 244 | 79 | 2 | 0 | 0 | 3 | 146 | post | positive | positive | | positive |
| 308 | live | 224 | 76 | 2 | 0 | 0 | 2 | 52 | post | positive | negative | | NA |
| 309 | live | 198 | 41 | 3 | 0 | 0 | 4 | 195 | pre | negative | negative | | negative |
| 310 | live | 5 | 56 | 2 | 0 | 0 | 2 | 53 | post | positive | negative | | NA |
| 311 | live | 10 | 55 | 2 | 0 | 0 | 2 | 7 | pre | negative | negative | | negative |
| 312 | live | 10 | 70 | 3 | 0 | 2 | 2 | 7 | post | positive | negative | | negative |
| 313 | live | 10 | 62 | 2 | 0 | 0 | 2 | 92 | post | negative | negative | | NA |
| 314 | live | 196 | 71 | 2 | 0 | 0 | 2 | 57 | post | positive | positive | | positive |
| 315 | live | 172 | 32 | 1 | 0 | 0 | 1 | 81 | pre | positive | positive | | negative |
| 316 | live | 242 | 59 | 4 | 1 | 2 | 2 | 22 | post | positive | negative | | negative |
| 317 | live | 213 | 88 | 3 | 0 | 0 | 4 | 17 | post | positive | positive | | negative |
| 318 | live | 231 | 62 | 2 | 0 | 0 | 2 | 33 | post | negative | negative | | negative |
| 319 | live | 218 | 61 | 1 | 0 | 0 | 1 | 479 | post | positive | positive | | negative |
| 320 | live | 191 | 55 | 1 | 0 | 0 | 1 | 43 | post | positive | negative | | negative |
| 321 | live | 183 | 63 | 2 | 0 | 1 | 2 | 87 | post | positive | positive | | positive |
| 322 | live | 10 | 58 | 2 | 0 | 0 | 2 | 0 | post | negative | negative | | positive |
| 323 | live | 11 | 67 | 3 | 0 | 2 | 2 | 176 | post | positive | NA | | negative |
| 324 | live | 10 | 52 | 2 | 0 | 0 | 2 | 61 | post | negative | negative | | negative |
| 325 | live | 10 | 55 | 1 | 0 | 0 | 1 | 71 | post | positive | positive | | negative |
| 326 | live | 10 | 45 | 3 | 0 | 2 | 2 | 26 | pre | positive | negative | | NA |
| 327 | live | 16 | 56 | 2 | 0 | 0 | 2 | 44 | post | negative | negative | | negative |
| 328 | live | 10 | 61 | 2 | 0 | 0 | 2 | 46 | post | positive | positive | | negative |
| 329 | live | 10 | 68 | 3 | 0 | 2 | 2 | 75 | post | negative | positive | | NA |
| 330 | live | 375 | 59 | 3 | 0 | 2 | 2 | 3 | post | positive | negative | | negative |
| 331 | live | 9 | 69 | 3 | 0 | 2 | 2 | 1 | post | positive | positive | | NA |
| 332 | live | 10 | 55 | 3 | 0 | 2 | 2 | 1 | post | positive | negative | | NA |
| 333 | live | 10 | 63 | 3 | 0 | 2 | 2 | 279 | post | negative | positive | | NA |
| 334 | live | 10 | 54 | 3 | 0 | 2 | 2 | 17 | post | positive | negative | | NA |
| 335 | live | 10 | 54 | 2 | 0 | 0 | 2 | 57 | post | negative | negative | | NA |
| 336 | live | 162 | 67 | 3 | 0 | 2 | 2 | 93 | post | positive | positive | | negative |
| 337 | live | 170 | 36 | 3 | 0 | 2 | 2 | 183 | pre | positive | positive | | negative |
| 338 | live | 2442 | 34 | 3 | 0 | 2 | 3 | 123 | pre | positive | positive | | negative |
| 339 | live | 1455 | 29 | 1 | 0 | 0 | 1 | 268 | pre | positive | positive | | negative |
| 340 | live | 1645 | 59 | 2 | 0 | 1 | 2 | 5 | post | positive | positive | | negative |
| 341 | dead | 2483 | 69 | 2 | 0 | 0 | 2 | 72 | post | positive | positive | | negative |
| 342 | live | 2576 | 54 | 2 | 0 | 1 | 2 | 47 | post | positive | positive | | negative |
| 343 | live | 1707 | 57 | 2 | 0 | 1 | 2 | 156 | post | positive | positive | | negative |
| 344 | live | 1187 | 42 | 2 | 0 | 1 | 2 | 158 | pre | positive | positive | | negative |
| 345 | dead | 1793 | 31 | 1 | 0 | 0 | 1 | 244 | pre | negative | negative | | negative |
| 346 | live | 886 | 41 | 1 | 0 | 0 | 1 | 135 | post | positive | positive | | negative |
| 347 | live | 585 | 41 | 1 | 0 | 0 | 1 | 139 | pre | negative | negative | | negative |
| 348 | live | 302 | 67 | 2 | 0 | 1 | 2 | 35 | post | positive | positive | | negative |
| 349 | live | 294 | 41 | 1 | 0 | 0 | 1 | 364 | pre | negative | negative | | NA |
| 350 | live | 735 | 48 | 4 | 1 | 1 | 4 | 117 | pre | positive | negative | | negative |
| 351 | live | 775 | 61 | 2 | 0 | 0 | 2 | 98 | post | negative | negative | | negative |
| 352 | live | 261 | 71 | 2 | 0 | 1 | 2 | 3 | post | positive | positive | | negative |
| 353 | live | 277 | 61 | 2 | 0 | 0 | 2 | 166 | post | positive | positive | | negative |
| 354 | live | 1256 | 61 | 3 | 0 | 3 | 2 | 404 | post | positive | positive | | negative |
| 355 | live | 346 | 36 | 3 | 0 | 3 | 2 | 117 | pre | positive | positive | | negative |
| 356 | live | 1150 | 50 | 3 | 0 | 1 | 3 | 52 | pre | positive | positive | | negative |
| 357 | live | 1547 | 64 | 2 | 0 | 0 | 2 | 1386 | post | positive | positive | | negative |
| 358 | live | 1813 | 59 | 3 | 0 | 1 | 3 | 50 | post | positive | positive | | negative |
| 359 | live | 1966 | 53 | 3 | 0 | 2 | 2 | 91 | post | negative | negative | | positive |
| 360 | live | 354 | 68 | 2 | 0 | 1 | 1 | 192 | post | positive | positive | | negative |
| 361 | live | 449 | 49 | 3 | 0 | 2 | 2 | 317 | post | positive | positive | | negative |
| 362 | live | 1172 | 56 | 2 | 0 | 1 | 1 | 101 | post | positive | negative | | negative |
| 363 | live | 1512 | 54 | 2 | 0 | 1 | 2 | 250 | post | positive | positive | | negative |
| 364 | live | 1319 | 59 | 3 | 0 | 2 | 2 | 14 | post | negative | negative | | negative |
| 365 | live | 1826 | 40 | 2 | 0 | 1 | 2 | 32 | pre | positive | positive | | positive |
| 366 | live | 3120 | 38 | 2 | 0 | 0 | 2 | 26 | pre | negative | negative | | negative |
| 367 | live | 3019 | 72 | 2 | 0 | 0 | 2 | 8 | post | positive | positive | | negative |
| 368 | live | 2850 | 39 | 2 | 0 | 0 | 2 | 150 | pre | positive | positive | | negative |
| 369 | live | 2877 | 61 | 2 | 0 | 0 | 2 | 532 | post | negative | negative | | negative |
| 370 | live | 2923 | 29 | 2 | 0 | 1 | 2 | 169 | pre | negative | negative | | negative |
| 371 | live | 2755 | 47 | 2 | 0 | 0 | 2 | 122 | pre | positive | positive | | negative |
| 372 | live | 2359 | 63 | 2 | 0 | 0 | 2 | 24 | post | positive | positive | | negative |
| 373 | live | 1994 | 42 | 2 | 0 | 1 | 2 | 6 | pre | positive | positive | | positive |
| 374 | live | 1948 | 43 | 2 | 0 | 1 | 1 | 130 | pre | negative | negative | | positive |
| 375 | live | 1743 | 51 | 2 | 0 | 0 | 3 | 59 | pre | positive | positive | | negative |
| 376 | live | 1471 | 36 | 2 | 0 | 0 | 2 | 53 | pre | negative | negative | | negative |
| 377 | live | 1266 | 75 | 2 | 0 | 0 | 2 | 100 | post | positive | positive | | negative |
| 378 | live | 863 | 69 | 2 | 0 | 0 | 2 | 12 | pre | positive | positive | | negative |
| 379 | live | 448 | 46 | 2 | 0 | 0 | 3 | 170 | pre | positive | positive | | negative |
| 380 | live | 2513 | 77 | 2 | 0 | 1 | 1 | 33 | post | positive | positive | | negative |
| 381 | live | 1519 | 84 | 3 | 0 | 1 | 4 | 132 | NA | positive | positive | | negative |
| 382 | live | 2141 | 51 | 2 | 0 | 0 | 2 | 64 | pre | negative | negative | | negative |
| 383 | live | 16 | 69 | 2 | 0 | 0 | 2 | 105 | post | positive | positive | | negative |
| 384 | live | 541 | 78 | 2 | 0 | 1 | 1 | 48 | post | positive | positive | | negative |
| 385 | live | 439 | 61 | 3 | NA | 2 | 2 | 61 | post | positive | positive | | positive |
| 386 | live | 160 | 45 | 2 | 0 | 0 | 2 | 493 | pre | negative | negative | | negative |
| 387 | live | 3359 | 48 | 2 | NA | 0 | 2 | 6 | post | positive | negative | | positive |
| 388 | dead | 172 | 70 | 3 | NA | 2 | 2 | 20 | post | positive | positive | | positive |
| 389 | live | 230 | 84 | 3 | NA | 2 | 2 | 11 | post | positive | positive | | positive |
| 390 | live | 162 | 49 | 3 | NA | 3 | 2 | 219 | pre | positive | positive | | negative |
| 391 | live | 78 | 51 | 2 | 0 | 0 | 2 | 4 | post | negative | negative | | negative |
| 392 | live | 665 | 51 | 2 | 0 | 0 | 2 | 33 | NA | positive | positive | | negative |
| 393 | live | 304 | 55 | 3 | 0 | 2 | 2 | 227 | post | positive | positive | | NA |
| 394 | live | 2521 | 43 | 2 | 0 | 0 | 2 | 2 | pre | positive | negative | | NA |
| 395 | live | 1500 | 27 | 3 | 0 | 1 | 3 | 375 | pre | positive | negative | | positive |
| 396 | dead | 160 | 68 | 2 | 0 | 1 | 2 | 38 | post | positive | positive | | negative |
| 397 | live | 1138 | 46 | 2 | 0 | 1 | 2 | 399 | pre | negative | negative | | negative |
| 398 | live | 1730 | 53 | 3 | 0 | 2 | 2 | 275 | pre | positive | negative | | negative |
| 399 | live | 360 | 35 | 2 | 0 | 0 | 2 | 87 | pre | negative | negative | | negative |
| 400 | live | 905 | 66 | 2 | 0 | 0 | 2 | 79 | post | positive | positive | | negative |
| 401 | live | 739 | 50 | 3 | 0 | 1 | 3 | 92 | pre | positive | positive | | negative |
| 402 | live | 15 | 64 | 2 | 0 | 1 | 1 | 265 | post | positive | positive | | positive |
| 403 | dead | 1699 | 54 | 2 | 0 | 0 | 2 | 35 | post | positive | negative | | NA |
| 404 | live | 2316 | 43 | 3 | 1 | 2 | 2 | 26 | pre | positive | positive | | negative |
| 405 | live | 8 | 73 | 2 | 0 | 1 | 2 | 90 | post | negative | negative | | NA |
| 406 | live | 2134 | 36 | 2 | 0 | 1 | 2 | NA | pre | negative | negative | | negative |
| 407 | dead | 2551 | 47 | 3 | 0 | 2 | 2 | 116 | pre | positive | positive | | negative |
| 408 | live | 2317 | 59 | 2 | 0 | 1 | 2 | 19 | post | positive | positive | | negative |
| 409 | live | 1684 | 54 | 2 | 0 | 0 | 2 | 72 | pre | negative | negative | | NA |
| 410 | live | 2298 | 51 | 2 | 0 | 1 | 2 | 17 | post | positive | negative | | NA |
| 411 | live | 1881 | 47 | 2 | 0 | 0 | 2 | 126 | post | negative | negative | | negative |
| 412 | live | 1605 | 83 | 1 | 0 | 0 | 1 | 194 | post | positive | negative | | NA |
| 413 | live | 1559 | 70 | 1 | 0 | 0 | 1 | 138 | post | positive | positive | | negative |
| 414 | live | 1485 | 60 | 3 | 0 | 1 | 3 | 252 | post | positive | positive | | negative |
| 415 | live | 1903 | 52 | 3 | 0 | 1 | 3 | 154 | post | positive | positive | | negative |
| 416 | live | 1330 | 46 | 2 | 0 | 0 | 2 | 126 | pre | positive | positive | | NA |
| 417 | live | 1325 | 47 | 2 | 0 | 1 | 1 | 698 | pre | positive | negative | | negative |
| 418 | live | 1216 | 80 | 1 | 0 | 0 | 1 | 35 | post | positive | positive | | negative |
| 419 | live | 1310 | 49 | 2 | 0 | 0 | 2 | 162 | post | negative | negative | | negative |
| 420 | dead | 524 | 50 | 3 | 0 | 2 | 1 | 423 | post | negative | negative | | NA |
| 421 | live | 1150 | 54 | 2 | 0 | 1 | 2 | 0 | post | positive | positive | | negative |
| 422 | dead | 1272 | 62 | 2 | 0 | 0 | 2 | 117 | post | positive | positive | | positive |
| 423 | live | 1409 | 39 | 3 | 0 | 2 | 2 | 88 | pre | positive | positive | | negative |
| 424 | live | 1295 | 68 | 2 | 0 | 1 | 2 | 13 | NA | positive | positive | | negative |
| 425 | live | 1072 | 65 | 2 | 0 | 0 | 2 | 536 | post | positive | positive | | negative |
| 426 | live | 1103 | 64 | 1 | 0 | 0 | 1 | 217 | post | positive | positive | | positive |
| 427 | live | 615 | 65 | 1 | 0 | 0 | 1 | 9 | post | negative | negative | | NA |
| 428 | live | 3333 | 65 | 2 | 0 | 0 | 2 | 90 | post | positive | positive | | negative |
| 429 | live | 1548 | 46 | 2 | 0 | 0 | 2 | 7 | pre | positive | positive | | negative |
| 430 | live | 2221 | 26 | 2 | 0 | 1 | 2 | 55 | pre | positive | positive | | negative |
| 431 | live | 1991 | 38 | 3 | 0 | 2 | 2 | 47 | pre | positive | positive | | negative |
| 432 | live | 2074 | 54 | 1 | 0 | 0 | 1 | 51 | post | positive | positive | | negative |
| 433 | live | 1998 | 43 | 3 | 0 | 2 | 3 | 218 | pre | positive | positive | | NA |
| 434 | live | 26 | 47 | 1 | 0 | 0 | 1 | 93 | post | positive | positive | | negative |
| 435 | live | 2009 | 49 | 2 | 0 | 0 | 3 | 114 | post | positive | negative | | NA |
| 436 | live | 1746 | 45 | 3 | 0 | 2 | 1 | 117 | pre | positive | positive | | negative |
| 437 | live | 1888 | 61 | 1 | 0 | 0 | 1 | 196 | post | positive | positive | | NA |
| 438 | live | 1620 | 46 | 3 | 0 | 3 | 3 | 105 | pre | positive | positive | | negative |
| 439 | live | 1623 | 47 | 2 | 0 | 1 | 1 | 526 | pre | negative | negative | | positive |
| 440 | live | 1648 | 52 | 2 | 0 | 1 | 2 | 34 | post | positive | positive | | NA |
| 441 | live | 1550 | 55 | 2 | 0 | 1 | 2 | 61 | post | positive | positive | | NA |
| 442 | live | 1465 | 52 | 2 | 0 | 0 | 2 | 66 | pre | positive | positive | | negative |
| 443 | live | 1574 | 57 | 2 | 0 | 0 | 2 | 11 | post | positive | positive | | NA |
| 444 | live | 1708 | 58 | 2 | 0 | 0 | 2 | 80 | post | positive | negative | | positive |
| 445 | live | 1374 | 51 | 3 | 0 | 2 | 2 | 147 | pre | positive | negative | | negative |
| 446 | live | 1236 | 50 | 1 | 0 | 0 | 1 | 968 | pre | positive | positive | | NA |
| 447 | live | 1212 | 50 | 3 | 0 | 2 | 2 | 293 | pre | positive | positive | | positive |
| 448 | live | 1059 | 62 | 1 | 0 | 0 | 1 | 115 | post | positive | positive | | positive |
| 449 | dead | 2854 | 45 | 2 | 0 | 0 | 2 | 13 | pre | negative | negative | | negative |
| 450 | live | 3913 | 69 | 1 | 0 | 0 | 1 | 71 | post | positive | negative | | NA |
| 451 | dead | 616 | 55 | 3 | 0 | 3 | 3 | 313 | post | negative | negative | | negative |
| 452 | live | 1359 | 40 | 3 | NA | 1 | 3 | 325 | pre | positive | positive | | positive |
| 453 | live | 1304 | 62 | 3 | 0 | 2 | 3 | 21 | post | positive | positive | | negative |
| 454 | live | 224 | 70 | 2 | 0 | 0 | 3 | 33 | post | positive | positive | | negative |
| 455 | live | 1218 | 49 | 2 | 0 | 1 | 1 | 99 | pre | positive | positive | | negative |
| 456 | live | 520 | 65 | 2 | 0 | 0 | 2 | 79 | post | positive | positive | | negative |
| 457 | live | 633 | 46 | 2 | 0 | 1 | 2 | 110 | pre | positive | positive | | negative |
| 458 | live | 516 | 59 | 2 | 0 | 0 | 3 | 89 | post | positive | positive | | negative |
| 459 | live | 620 | 49 | 1 | 0 | 0 | 1 | 37 | post | negative | negative | | NA |
| 460 | live | 1440 | 62 | 2 | 0 | 0 | 2 | 56 | post | positive | positive | | NA |
| 461 | live | 396 | 68 | 3 | 0 | 3 | 2 | 109 | post | positive | positive | | positive |
| 462 | live | 439 | 54 | 2 | 0 | 1 | 2 | 147 | post | positive | positive | | NA |
| 463 | dead | 322 | 68 | 3 | 0 | 1 | 3 | 172 | post | negative | negative | | negative |
| 464 | dead | 2361 | 73 | 2 | 0 | 0 | 2 | 5 | post | negative | negative | | NA |
| 465 | live | 3827 | 45 | 1 | 0 | 0 | 1 | 205 | NA | NA | NA | | NA |
| 466 | live | 4550 | 49 | 2 | 0 | 1 | 2 | 136 | NA | positive | positive | | NA |
| 467 | dead | 991 | 49 | 2 | 0 | 1 | 1 | 11 | NA | negative | negative | | NA |
| 468 | dead | 749 | 46 | NA | 0 | NA | 1 | 15 | NA | NA | NA | | NA |
| 469 | dead | 362 | 62 | 4 | 1 | NA | 3 | 59 | NA | NA | positive | | NA |
| 470 | live | 6719 | 51 | 2 | 0 | 0 | 2 | 15 | NA | positive | positive | | NA |
| 471 | dead | 3941 | 64 | 4 | 1 | 3 | 3 | 8 | NA | positive | positive | | NA |
| 472 | dead | 1542 | 90 | NA | NA | NA | 2 | 1 | post | positive | positive | | NA |
| 473 | dead | 1993 | 38 | 3 | 0 | 1 | 3 | 91 | pre | negative | negative | | NA |
| 474 | dead | 4456 | 50 | 2 | 0 | 1 | 2 | 83 | NA | positive | positive | | NA |
| 475 | dead | 3418 | 81 | 3 | 0 | 1 | 3 | 178 | post | positive | positive | | NA |
| 476 | live | 5383 | 42 | 2 | 0 | 0 | 3 | 202 | NA | positive | positive | | NA |
| 477 | dead | 571 | 63 | 3 | 0 | 1 | 4 | 68 | NA | negative | negative | | NA |
| 478 | live | 3836 | 75 | 2 | 0 | 0 | 3 | 23 | post | positive | positive | | NA |
| 479 | dead | 2573 | 45 | NA | NA | NA | 1 | 422 | NA | positive | negative | | NA |
| 480 | live | 3350 | 66 | 2 | 0 | 0 | 2 | 64 | NA | positive | NA | | NA |
| 481 | live | 3374 | 74 | 1 | 0 | 0 | 1 | 80 | post | positive | positive | | NA |
| 482 | live | 3872 | 40 | 3 | 0 | 1 | 3 | 109 | NA | negative | negative | | NA |
| 483 | live | 6435 | 61 | NA | 0 | 0 | NA | 22 | NA | negative | negative | | NA |
| 484 | live | 2082 | 26 | 2 | 0 | 0 | 3 | 7 | NA | negative | negative | | NA |
| 485 | live | 5749 | 51 | 2 | 0 | 0 | 2 | 2 | NA | positive | positive | | NA |
| 486 | live | 6796 | 44 | 3 | 0 | 1 | 4 | 18 | NA | positive | positive | | NA |
| 487 | dead | 2469 | 60 | 2 | 0 | 0 | 2 | 0 | NA | positive | positive | | NA |
| 488 | dead | 2373 | 57 | NA | NA | NA | NA | 9 | NA | positive | positive | | NA |
| 489 | live | 5261 | 60 | 1 | 0 | 0 | 1 | 54 | post | negative | negative | | NA |
| 490 | live | 4929 | 71 | 3 | 0 | 1 | 4 | 68 | post | positive | positive | | NA |
| 491 | dead | 3126 | 73 | NA | 0 | 1 | 2 | 67 | post | positive | positive | | NA |
| 492 | dead | 4267 | 68 | 2 | 0 | 0 | 3 | 9 | post | positive | positive | | NA |
| 493 | dead | 3063 | 38 | 2 | 0 | 0 | 2 | 129 | NA | negative | negative | | NA |
| 494 | live | 2721 | 39 | 3 | 0 | 1 | 3 | 799 | NA | negative | negative | | NA |
| 495 | live | 7067 | 40 | 1 | 0 | 0 | 1 | 66 | NA | negative | negative | | NA |
| 496 | live | 4788 | 42 | 3 | 0 | 2 | 3 | 186 | NA | positive | positive | | NA |
| 497 | dead | 2965 | 58 | NA | NA | NA | 1 | 329 | NA | positive | positive | | NA |
| 498 | live | 5396 | 61 | 2 | 0 | 1 | 2 | 2 | post | positive | positive | | NA |
| 499 | dead | 2417 | 67 | 2 | 0 | 1 | 2 | 5 | post | positive | positive | | NA |
| 500 | dead | 558 | 58 | NA | NA | 1 | 2 | 15 | NA | positive | positive | | NA |
| 501 | dead | 639 | 40 | 3 | 0 | 1 | 3 | 195 | pre | negative | negative | | NA |
| 502 | dead | 3461 | 40 | 3 | 0 | 1 | 3 | 47 | pre | positive | negative | | NA |
| 503 | live | 3941 | 50 | 2 | 0 | 1 | 2 | 4 | NA | positive | positive | | NA |
| 504 | dead | 3945 | 54 | 1 | 0 | 0 | 1 | 26 | NA | positive | positive | | NA |
| 505 | live | 5677 | 48 | 2 | 1 | 1 | 2 | 3 | NA | negative | negative | | NA |
| 506 | dead | 860 | 62 | 2 | 0 | 1 | 2 | 2 | post | positive | positive | | NA |
| 507 | dead | 2097 | 61 | 2 | 0 | 1 | 2 | 5 | post | positive | positive | | NA |
| 508 | dead | 1781 | 62 | NA | NA | NA | 1 | 34 | post | positive | positive | | NA |
| 509 | live | 1326 | 67 | 2 | 0 | 1 | 2 | 1 | post | positive | negative | | NA |
| 510 | live | 3088 | 68 | 2 | 0 | 1 | 2 | 0 | post | negative | negative | | NA |
| 511 | live | 1463 | 63 | 1 | 0 | 0 | 1 | 33 | post | positive | positive | | negative |
| 512 | live | 3330 | 57 | 3 | 0 | 1 | 4 | 114 | post | negative | negative | | NA |
| 513 | live | 4840 | 62 | 2 | 0 | 0 | 2 | 15 | NA | positive | positive | | NA |
| 514 | live | 1132 | 50 | 4 | 1 | 1 | 3 | 653 | NA | negative | negative | | negative |
| 515 | live | 215 | 43 | 3 | 0 | 2 | 2 | 59 | NA | negative | negative | | negative |
| 516 | live | 1989 | 47 | 2 | 0 | 1 | 1 | 97 | NA | positive | positive | | negative |
| 517 | live | 1651 | 47 | 1 | 0 | 0 | 1 | 8 | NA | negative | negative | | negative |
| 518 | live | 1519 | 55 | 3 | 0 | 3 | 2 | 73 | NA | positive | positive | | negative |
| 519 | dead | 573 | 44 | 3 | 0 | 2 | 1 | 107 | pre | negative | negative | | negative |
| 520 | live | 2521 | 76 | 1 | 0 | 0 | 1 | 52 | post | positive | positive | | negative |
| 521 | live | 1673 | 51 | 2 | 0 | 0 | 3 | 54 | NA | positive | positive | | negative |
| 522 | live | 746 | 45 | 2 | 0 | 0 | 2 | 93 | pre | positive | positive | | negative |
| 523 | live | 1180 | 52 | 1 | 0 | 0 | 1 | 25 | NA | negative | negative | | negative |
| 524 | live | 622 | 56 | 2 | 0 | 1 | 1 | 485 | post | positive | negative | | positive |
| 525 | live | 777 | 62 | 2 | 0 | 0 | 2 | 92 | post | positive | positive | | negative |
| 526 | live | 867 | 47 | 3 | 0 | 1 | 3 | 99 | pre | positive | positive | | NA |
| 527 | live | 1428 | 56 | 1 | 0 | 0 | 1 | 209 | post | positive | positive | | NA |
| 528 | live | 1148 | 66 | 2 | 0 | 1 | 2 | 105 | post | positive | positive | | negative |
| 529 | live | 1203 | 53 | 2 | 0 | 1 | 2 | 56 | post | negative | negative | | negative |
| 530 | live | 1191 | 65 | 3 | 0 | 2 | 2 | 71 | NA | positive | positive | | positive |
| 531 | live | 1471 | 40 | 3 | 0 | 2 | 2 | 47 | pre | positive | positive | | NA |
| 532 | live | 1732 | 47 | 1 | 0 | 0 | 1 | 480 | NA | positive | positive | | NA |
| 533 | live | 1733 | 42 | 2 | 0 | 1 | 2 | 163 | pre | positive | positive | | positive |
| 534 | live | 1569 | 64 | 1 | 0 | 0 | 1 | 14 | post | positive | positive | | negative |
| 535 | live | 1572 | 44 | 1 | 0 | 0 | 1 | 243 | pre | negative | negative | | negative |
| 536 | live | 1132 | 51 | 3 | 0 | 3 | 3 | 264 | pre | positive | positive | | negative |
| 537 | live | 974 | 60 | 3 | 0 | 3 | 2 | 147 | post | positive | positive | | negative |
| 538 | live | 554 | 47 | 2 | 0 | 1 | 1 | 152 | pre | positive | positive | | negative |
| 539 | live | 782 | 56 | 2 | 0 | 1 | 2 | 317 | post | positive | positive | | NA |
| 540 | live | 756 | 73 | 1 | 0 | 0 | 1 | 172 | post | negative | negative | | NA |
| 541 | live | 660 | 41 | 2 | 0 | 1 | 2 | 241 | pre | positive | positive | | negative |
| 542 | live | 1340 | 35 | 1 | 0 | 0 | 1 | 269 | pre | negative | negative | | NA |
| 543 | live | 1117 | 54 | 2 | 0 | 1 | 2 | 76 | post | positive | negative | | negative |
| 544 | live | 1085 | 54 | 1 | 0 | 0 | 1 | 83 | pre | positive | positive | | NA |
| 545 | live | 1481 | 76 | 1 | 0 | 0 | 1 | 17 | post | positive | positive | | NA |
| 546 | live | 827 | 39 | 1 | 0 | 0 | 1 | 140 | pre | positive | positive | | NA |
| 547 | live | 1633 | 59 | 1 | 0 | 0 | 1 | 170 | post | positive | positive | | NA |
| 548 | live | 1641 | 55 | 3 | 0 | 1 | 3 | 23 | post | positive | positive | | negative |
| 549 | live | 1386 | 56 | 2 | 0 | 1 | 1 | 62 | NA | positive | positive | | NA |
| 550 | live | 1519 | 78 | 2 | 0 | 1 | 2 | 49 | post | positive | positive | | negative |
| 551 | live | 355 | 71 | 1 | 0 | 0 | 1 | 58 | post | negative | negative | | negative |
| 552 | live | 1492 | 59 | 3 | 0 | 1 | 3 | 150 | post | positive | positive | | NA |
| 553 | live | 1270 | 62 | 2 | 0 | 1 | 1 | 189 | post | positive | positive | | positive |
| 554 | live | 1339 | 61 | 3 | 0 | 2 | 3 | 156 | post | positive | positive | | NA |
| 555 | live | 1464 | 47 | 1 | 0 | 0 | 1 | 199 | pre | positive | negative | | NA |
| 556 | live | 1305 | 48 | 2 | 0 | 1 | 2 | 104 | post | positive | negative | | positive |
| 557 | live | 1394 | 58 | 2 | 0 | 1 | 2 | 16 | NA | positive | positive | | positive |
| 558 | live | 948 | 62 | 2 | 0 | 0 | 2 | 124 | post | positive | positive | | NA |
| 559 | live | 713 | 30 | 2 | 0 | 0 | 2 | 111 | pre | positive | negative | | NA |
| 560 | live | 1156 | 63 | 2 | 0 | 1 | 2 | 60 | post | positive | positive | | negative |
| 561 | live | 673 | 63 | 2 | 0 | 1 | 2 | 74 | post | positive | positive | | NA |
| 562 | live | 423 | 49 | 2 | 0 | 0 | 2 | 69 | pre | positive | positive | | negative |
| 563 | live | 1467 | 64 | 2 | 0 | 0 | 2 | 303 | post | positive | negative | | NA |
| 564 | live | 525 | 78 | 1 | 0 | 0 | 1 | 53 | post | positive | positive | | NA |
| 565 | live | 476 | 60 | 2 | 0 | 0 | 3 | 110 | post | positive | positive | | negative |
| 566 | live | 98 | 42 | 2 | 0 | 1 | 2 | 79 | pre | positive | positive | | negative |
| 567 | live | 78 | 71 | 3 | 0 | 2 | 2 | 109 | post | positive | positive | | negative |
| 568 | live | 1170 | 41 | 2 | 0 | 1 | 1 | 80 | pre | positive | positive | | NA |
| 569 | live | 1374 | 54 | 3 | 0 | 2 | 2 | 136 | post | positive | positive | | NA |
| 570 | live | 1442 | 62 | 1 | 0 | 0 | 1 | 238 | post | positive | positive | | NA |
| 571 | live | 495 | 43 | 2 | 0 | 1 | 2 | 42 | pre | positive | positive | | positive |
| 572 | live | 134 | 38 | 3 | 0 | 3 | 3 | 63 | pre | negative | negative | | negative |
| 573 | live | 477 | 52 | 2 | 0 | 1 | 2 | 68 | post | positive | positive | | negative |
| 574 | live | 376 | 49 | 3 | 0 | 2 | 2 | 74 | pre | positive | positive | | negative |
| 575 | live | 293 | 69 | 1 | 0 | 0 | 1 | 117 | post | negative | negative | | negative |
| 576 | live | 1363 | 79 | 2 | 0 | 1 | 2 | 9 | post | positive | positive | | negative |
| 577 | live | 1405 | 53 | 2 | 0 | 1 | 2 | 183 | post | positive | positive | | NA |
| 578 | dead | 991 | 72 | 2 | 0 | 1 | 1 | 44 | post | positive | positive | | NA |
| 579 | live | 745 | 69 | 1 | 0 | 0 | 1 | 136 | post | positive | positive | | negative |
| 580 | live | 943 | 68 | 2 | 0 | 0 | 3 | 136 | post | negative | negative | | positive |
| 581 | live | 743 | 51 | 2 | 0 | 1 | 1 | 16 | post | positive | positive | | NA |
| 582 | live | 917 | 67 | 2 | 0 | 1 | 2 | 53 | post | positive | positive | | negative |
| 583 | live | 328 | 62 | 2 | 0 | 1 | 1 | 56 | post | positive | positive | | negative |
| 584 | live | 461 | 69 | 1 | 0 | 0 | 1 | 41 | post | positive | positive | | negative |
| 585 | live | 1149 | 46 | 1 | 0 | 0 | 1 | 304 | pre | positive | positive | | negative |
| 586 | live | 1080 | 45 | 1 | 0 | 0 | 1 | 74 | post | positive | positive | | negative |
| 587 | live | 747 | 82 | 1 | 0 | NA | 1 | 2 | post | positive | positive | | negative |
| 588 | live | 702 | 65 | 3 | 0 | 2 | 1 | 86 | post | positive | positive | | negative |
| 589 | live | 1247 | 69 | 2 | 0 | 0 | 2 | 47 | post | positive | positive | | negative |
| 590 | live | 856 | 31 | 1 | 0 | 0 | 1 | 105 | pre | positive | positive | | negative |
| 591 | live | 806 | 55 | 1 | 0 | 0 | 1 | 213 | post | positive | positive | | negative |
| 592 | live | 727 | 77 | 1 | 0 | 0 | 1 | 163 | post | positive | positive | | negative |
| 593 | live | 620 | 78 | 1 | 0 | 0 | 1 | 39 | post | positive | positive | | negative |
| 594 | live | 178 | 81 | 2 | 0 | 1 | 2 | 67 | post | positive | negative | | negative |
| 595 | live | 72 | 56 | 2 | 0 | 1 | 2 | 2 | post | positive | positive | | negative |
| 596 | live | 516 | 67 | 1 | 0 | 0 | 1 | 0 | post | positive | positive | | negative |
| 597 | live | 76 | 48 | 2 | 0 | 1 | 1 | 39 | pre | positive | positive | | negative |
| 598 | live | 414 | 65 | 3 | 0 | 2 | 3 | 160 | post | positive | negative | | negative |
| 599 | live | 1121 | 56 | 2 | 0 | 0 | 2 | 23 | post | positive | positive | | negative |
| 600 | live | 392 | 52 | 1 | 0 | 0 | 1 | 67 | post | positive | positive | | negative |
| 601 | live | 1561 | 62 | 1 | 0 | 0 | 1 | 0 | post | positive | negative | | negative |
| 602 | live | 829 | 54 | 2 | 0 | 1 | 2 | 130 | post | positive | positive | | NA |
| 603 | live | 868 | 60 | 1 | 0 | 0 | 1 | 22 | post | positive | negative | | NA |
| 604 | live | 170 | 59 | 2 | 0 | 0 | 2 | 398 | post | negative | negative | | negative |
| 605 | live | 180 | 58 | 2 | 0 | 1 | 1 | 7 | NA | positive | positive | | negative |
| 606 | live | 175 | 46 | 2 | 0 | 0 | 2 | 23 | NA | positive | positive | | NA |
| 607 | live | 543 | 77 | 2 | 0 | 1 | 1 | 535 | NA | positive | positive | | NA |
| 608 | live | 558 | 49 | 1 | 0 | 0 | 1 | 372 | NA | positive | positive | | NA |
| 609 | live | 372 | 82 | 1 | 0 | 0 | 1 | 44 | NA | negative | negative | | NA |
| 610 | live | 268 | 50 | 2 | 0 | 0 | 2 | 145 | post | positive | positive | | NA |
| 611 | live | 61 | 81 | 1 | 0 | 0 | 1 | 29 | post | negative | negative | | NA |
| 612 | live | 0 | 63 | 1 | 0 | 0 | 1 | 204 | post | positive | positive | | NA |
| 613 | live | 220 | 53 | 2 | 0 | 1 | 1 | 452 | post | positive | positive | | NA |
| 614 | dead | 612 | 56 | 4 | 1 | 2 | 4 | 11 | NA | positive | positive | | NA |
| 615 | dead | 2763 | 46 | 1 | 0 | 0 | 1 | 31 | NA | positive | positive | | NA |
| 616 | dead | 811 | 50 | 3 | 0 | 1 | 3 | 20 | NA | positive | positive | | NA |
| 617 | dead | 2207 | 39 | 3 | 0 | 1 | 3 | 81 | NA | positive | positive | | positive |
| 618 | dead | 1148 | 88 | 2 | 0 | 1 | 2 | 4 | NA | positive | positive | | NA |
| 619 | dead | 921 | 60 | 1 | 0 | 0 | 1 | 47 | NA | positive | negative | | positive |
| 620 | dead | 1692 | 56 | 2 | 0 | 1 | 2 | 82 | NA | negative | negative | | negative |
| 621 | dead | 1142 | 50 | 2 | 0 | 1 | 2 | 137 | NA | NA | negative | | positive |
| 622 | dead | 2009 | 79 | 1 | 0 | 0 | 1 | 34 | post | positive | positive | | NA |
| 623 | dead | 224 | 70 | 2 | 0 | 0 | 2 | 27 | post | negative | negative | | negative |
| 624 | dead | 1563 | 72 | 3 | 0 | 2 | 2 | 19 | post | positive | positive | | positive |
| 625 | dead | 1556 | 48 | 2 | 0 | 1 | 2 | 77 | NA | negative | negative | | NA |
| 626 | dead | 2127 | 78 | 2 | 0 | NA | 2 | 14 | post | negative | negative | | NA |
| 627 | dead | 2798 | 68 | 2 | 0 | 1 | 1 | 86 | post | positive | positive | | NA |
| 628 | dead | 3462 | 35 | 2 | 0 | 1 | 2 | 8 | pre | positive | positive | | NA |
| 629 | dead | 2520 | 55 | 1 | 0 | 0 | 1 | 114 | NA | positive | positive | | NA |
| 630 | dead | 1286 | 83 | 1 | 0 | 0 | 1 | 74 | post | positive | positive | | NA |
| 631 | dead | 365 | 45 | 3 | 0 | 1 | 3 | 2 | NA | positive | positive | | NA |
| 632 | dead | 1694 | 38 | 2 | 0 | 1 | 2 | 101 | pre | negative | negative | | NA |
| 633 | dead | 1508 | 67 | 2 | 0 | 1 | 2 | 188 | post | positive | positive | | negative |
| 634 | dead | 538 | 79 | 2 | 0 | 0 | 2 | 138 | post | positive | positive | | NA |
| 635 | dead | 785 | 80 | 2 | 0 | 1 | 1 | 186 | post | negative | NA | | NA |
| 636 | dead | 959 | 53 | 3 | 0 | 1 | 4 | 98 | NA | positive | positive | | NA |
| 637 | dead | 2712 | 62 | 2 | 0 | 1 | 1 | 62 | NA | positive | positive | | NA |
| 638 | dead | 2965 | 51 | NA | NA | 2 | 4 | 114 | NA | negative | negative | | NA |
| 639 | dead | 763 | 90 | 3 | 0 | 2 | 4 | 24 | post | positive | positive | | NA |
| 640 | dead | 3669 | 60 | 2 | 0 | 1 | 2 | 192 | post | positive | positive | | NA |
| 641 | dead | 3472 | 78 | 2 | 0 | 1 | 1 | 157 | post | negative | negative | | NA |
| 642 | dead | 1009 | 68 | 1 | 0 | 0 | 1 | 8 | post | positive | positive | | NA |
| 643 | dead | 2273 | 31 | 2 | 0 | 1 | 2 | 34 | NA | positive | positive | | NA |
| 644 | dead | 3736 | 88 | 1 | 0 | NA | 1 | 36 | post | positive | positive | | NA |
| 645 | dead | 1034 | 47 | 4 | 1 | 1 | 2 | 173 | NA | positive | negative | | NA |
| 646 | dead | 1927 | 66 | 3 | 0 | 1 | 3 | 1 | NA | negative | positive | | NA |
| 647 | dead | 1673 | 69 | 2 | 0 | 1 | 2 | 68 | NA | positive | positive | | NA |
| 648 | dead | 1388 | 44 | 3 | 0 | 2 | 2 | 6 | NA | positive | negative | | NA |
| 649 | dead | 2192 | 34 | 2 | 0 | 0 | 2 | 13 | NA | positive | positive | | NA |
| 650 | dead | 1642 | 73 | 3 | 0 | 1 | 4 | 29 | NA | positive | positive | | NA |
| 651 | dead | 1688 | 44 | 1 | 0 | 0 | 1 | 122 | NA | negative | negative | | NA |
| 652 | live | 210 | 64 | 1 | 0 | 0 | 1 | 43 | post | positive | positive | | NA |
| 653 | live | 22 | 60 | 2 | 0 | 0 | 2 | 129 | post | positive | positive | | NA |
| 654 | dead | 1174 | 78 | 2 | 0 | 1 | 2 | 94 | post | NA | NA | | NA |
| 655 | dead | 2534 | 80 | 2 | 0 | 1 | 2 | 12 | post | NA | NA | | NA |
| 656 | dead | 1759 | 48 | 2 | 0 | 1 | 2 | 108 | NA | NA | NA | | NA |
| 657 | dead | 3959 | 77 | 1 | 0 | 0 | 1 | 371 | post | positive | positive | | NA |
| 658 | live | 324 | 50 | 3 | 0 | 3 | 3 | 246 | NA | positive | positive | | NA |
| 659 | live | 196 | 46 | 2 | 0 | 1 | 2 | 185 | pre | positive | positive | | NA |
| 660 | live | 168 | 45 | 2 | 0 | 0 | 2 | 172 | pre | positive | positive | | negative |
| 661 | live | 30 | 75 | 2 | 0 | 0 | 2 | 36 | post | positive | positive | | NA |
| 662 | live | 3324 | 80 | 2 | 0 | 0 | 2 | 348 | post | negative | negative | | NA |
| 663 | live | 272 | 41 | 1 | 0 | 1 | 1 | 329 | pre | positive | positive | | NA |
| 664 | live | 98 | 51 | 2 | 0 | 1 | 2 | 21 | post | positive | negative | | negative |
| 665 | live | 225 | 63 | 1 | 0 | 0 | 1 | 158 | post | positive | positive | | NA |
| 666 | live | 293 | 46 | 2 | 0 | 0 | 2 | 24 | NA | positive | positive | | negative |
| 667 | live | 160 | 61 | 2 | 0 | 0 | 2 | 101 | post | negative | positive | | NA |
| 668 | dead | 295 | 87 | 1 | 0 | 0 | 1 | 3 | post | positive | positive | | NA |
| 669 | live | 144 | 58 | 1 | 0 | 0 | 1 | 207 | post | positive | positive | | NA |
| 670 | live | 199 | 54 | 2 | 0 | 1 | 2 | 290 | post | positive | positive | | NA |
| 671 | live | 185 | 53 | 3 | 0 | 1 | 3 | 216 | NA | positive | positive | | NA |
| 672 | live | 0 | 80 | 2 | 0 | 1 | 2 | 183 | post | NA | NA | | NA |
| 673 | live | 0 | 67 | 2 | 0 | 0 | 2 | 19 | post | negative | negative | | NA |
| 674 | live | 0 | 70 | 2 | 0 | 0 | 2 | 43 | post | positive | negative | | NA |
| 675 | live | 0 | 58 | 2 | 0 | 0 | 2 | 16 | post | positive | positive | | NA |
| 676 | live | 0 | 50 | 2 | 0 | 0 | 2 | 53 | pre | positive | positive | | NA |
| 677 | live | 0 | 55 | 2 | 0 | 1 | 2 | 15 | post | negative | negative | | positive |
| 678 | live | 2 | 78 | 3 | 0 | 2 | 1 | 47 | post | negative | negative | | positive |
| 679 | live | 0 | 43 | 2 | 0 | 0 | 2 | 15 | pre | positive | positive | | positive |
| 680 | live | 0 | 46 | 2 | 0 | 1 | 2 | 89 | pre | positive | positive | | negative |
| 681 | live | 0 | 55 | 2 | 0 | 0 | 2 | 336 | post | negative | negative | | NA |
| 682 | live | 7 | 49 | 3 | 0 | 1 | 4 | 28 | pre | positive | positive | | negative |
| 683 | live | 0 | 62 | 2 | 0 | 1 | 2 | 6 | post | positive | positive | | negative |
| 684 | live | 0 | 44 | 2 | 0 | 1 | 2 | 30 | pre | NA | NA | | NA |
| 685 | live | 12 | 45 | 2 | 0 | 1 | 2 | 0 | post | negative | negative | | positive |
| 686 | live | 4 | 52 | 3 | 0 | 2 | 3 | 72 | post | positive | positive | | NA |
| 687 | live | 14 | 82 | 3 | 0 | 2 | 2 | 79 | post | negative | negative | | negative |
| 688 | live | 13 | 56 | 2 | 0 | 1 | 2 | 173 | post | positive | positive | | NA |
| 689 | live | 0 | 65 | 3 | 0 | 1 | 3 | 8 | post | positive | positive | | NA |
| 690 | live | 14 | 52 | 2 | 0 | 0 | 2 | 106 | post | negative | negative | | NA |
| 691 | live | 13 | 64 | 2 | 0 | 1 | 2 | 56 | post | negative | negative | | positive |
| 692 | live | 34 | 34 | 2 | 0 | 1 | 2 | 0 | pre | negative | negative | | positive |
| 693 | live | 7 | 54 | 3 | 0 | 2 | 2 | 191 | post | positive | negative | | NA |
| 694 | live | 0 | 59 | 2 | 0 | 0 | 2 | 64 | post | positive | positive | | NA |
| 695 | live | 0 | 48 | 2 | 0 | 0 | 2 | 291 | pre | negative | positive | | positive |
| 696 | live | 0 | 50 | 2 | 0 | 0 | 2 | 116 | pre | positive | positive | | negative |
| 697 | live | 0 | 40 | 3 | 0 | 2 | 2 | 136 | pre | positive | positive | | negative |
| 698 | live | 5 | 53 | 2 | 0 | 0 | 2 | 75 | post | negative | negative | | NA |
| 699 | live | 7 | 53 | 2 | 0 | 1 | 2 | 7 | post | negative | negative | | positive |
| 700 | live | 2 | 38 | 3 | 0 | 1 | 3 | 44 | pre | positive | negative | | NA |
| 701 | live | 6 | 74 | 2 | 0 | 0 | 2 | 110 | post | positive | positive | | negative |
| 702 | live | 2 | 56 | 2 | 0 | 0 | 2 | 22 | post | positive | positive | | NA |
| 703 | live | 0 | 34 | 3 | 0 | 3 | 3 | 85 | pre | positive | positive | | NA |
| 704 | live | 9 | 47 | 3 | 0 | 2 | 3 | 42 | post | positive | positive | | NA |
| 705 | live | 7 | 58 | 2 | 0 | 1 | 2 | 176 | post | positive | positive | | NA |
| 706 | live | 11 | 58 | 2 | 0 | 1 | 1 | 256 | post | negative | negative | | negative |
| 707 | live | 13 | 90 | 2 | 0 | 0 | 2 | 67 | post | negative | negative | | negative |
| 708 | live | 4 | 59 | 2 | 0 | 0 | 2 | 30 | post | positive | positive | | negative |
| 709 | live | 6 | 29 | 2 | 0 | 1 | 2 | 50 | pre | positive | positive | | NA |
| 710 | live | 1 | 63 | 2 | 0 | 0 | 2 | 11 | post | positive | positive | | negative |
| 711 | live | 1 | 56 | 2 | 0 | 1 | 1 | 275 | post | NA | NA | | NA |
| 712 | live | 9 | 61 | 3 | 0 | 2 | 2 | 154 | post | negative | negative | | positive |
| 713 | live | 371 | 48 | 2 | 0 | 1 | 2 | 20 | post | positive | positive | | negative |
| 714 | live | 30 | 48 | 2 | 0 | 0 | 3 | 93 | post | negative | negative | | NA |
| 715 | live | 1 | 60 | 3 | 0 | 0 | 4 | 160 | post | negative | negative | | NA |
| 716 | live | 7 | 68 | 1 | 0 | 0 | 1 | 20 | post | positive | positive | | positive |
| 717 | live | 8 | 59 | 3 | 0 | 2 | 2 | 88 | post | negative | negative | | NA |
| 718 | live | 9 | 53 | 2 | 0 | 1 | 2 | 71 | post | positive | positive | | NA |
| 719 | live | 10 | 49 | 3 | 0 | 2 | 3 | 189 | post | NA | NA | | NA |
| 720 | live | 7 | 49 | 2 | 0 | 1 | 2 | NA | pre | NA | NA | | NA |
| 721 | live | 264 | 52 | 1 | 0 | 0 | 1 | 2 | post | positive | positive | | negative |
| 722 | live | 210 | 51 | NA | 0 | 2 | 2 | 111 | post | negative | negative | | negative |
| 723 | live | 27 | 62 | 2 | 0 | 1 | 2 | 57 | post | positive | positive | | positive |
| 724 | live | 113 | 40 | NA | 0 | 1 | 1 | 318 | NA | positive | positive | | negative |
| 725 | live | 61 | 74 | 2 | 0 | 0 | 3 | 89 | post | negative | negative | | negative |
| 726 | live | 172 | 51 | 2 | 0 | 0 | 2 | 192 | post | negative | negative | | negative |
| 727 | live | 49 | 80 | 2 | 0 | 1 | 1 | 73 | post | positive | positive | | negative |
| 728 | live | 344 | 57 | 2 | NA | 0 | 2 | 132 | post | positive | positive | | negative |
| 729 | live | 2 | 45 | NA | 0 | 0 | 2 | 61 | NA | negative | negative | | NA |
| 730 | live | 256 | 77 | 2 | 0 | 1 | 2 | 231 | post | positive | positive | | NA |
| 731 | live | 248 | 48 | 1 | 0 | 0 | 1 | 26 | post | positive | negative | | positive |
| 732 | live | 18 | 60 | NA | 0 | NA | 4 | 18 | post | negative | negative | | positive |
| 733 | live | 365 | 54 | 2 | 0 | 1 | 2 | 268 | post | positive | positive | | positive |
| 734 | live | 178 | 59 | 3 | 0 | 2 | 2 | 52 | post | positive | positive | | negative |
| 735 | live | 273 | 41 | 2 | 0 | 1 | 2 | 40 | pre | positive | positive | | negative |
| 736 | live | 57 | 62 | 2 | 0 | 1 | 1 | 34 | post | positive | positive | | negative |
| 737 | live | 96 | 79 | 3 | 0 | 2 | 1 | 99 | post | negative | negative | | negative |
| 738 | live | 187 | 62 | 2 | 0 | 0 | 2 | 147 | post | negative | negative | | negative |
| 739 | live | 284 | 56 | 1 | 0 | 0 | 1 | 115 | post | positive | positive | | negative |
| 740 | live | 21 | 54 | 2 | NA | 1 | 1 | 13 | post | positive | positive | | NA |
| 741 | live | 274 | 54 | 2 | 0 | 0 | 2 | 13 | post | positive | positive | | negative |
| 742 | live | 109 | 90 | 2 | 0 | 0 | 2 | 104 | post | negative | positive | | negative |
| 743 | live | 265 | 72 | 2 | 0 | 0 | 2 | 147 | post | negative | negative | | negative |
| 744 | live | 238 | 59 | 2 | 0 | 1 | 2 | 97 | post | positive | negative | | negative |
| 745 | live | 217 | 80 | 3 | NA | 3 | 3 | 41 | post | positive | positive | | negative |
| 746 | live | 167 | 73 | 1 | 0 | 0 | 1 | 55 | post | positive | positive | | negative |
| 747 | live | 105 | 77 | 1 | 0 | 0 | 1 | 17 | post | positive | positive | | negative |
| 748 | live | 123 | 70 | 2 | 0 | 0 | 2 | 12 | post | positive | positive | | negative |
| 749 | live | 106 | 51 | 1 | 0 | 0 | 1 | 257 | post | positive | positive | | negative |
| 750 | live | 171 | 81 | 3 | NA | 3 | 2 | 22 | post | positive | positive | | positive |
| 751 | live | 236 | 80 | 3 | NA | 2 | 3 | 40 | post | positive | positive | | negative |
| 752 | live | 85 | 40 | 2 | NA | 0 | 2 | 54 | pre | positive | positive | | NA |
| 753 | live | 272 | 62 | 3 | 0 | 2 | 1 | 170 | post | positive | NA | | NA |
| 754 | live | 380 | 66 | 2 | 0 | 1 | 2 | 44 | post | positive | positive | | negative |
| 755 | live | 274 | 64 | 1 | 0 | 0 | 1 | 28 | post | positive | positive | | NA |
| 756 | live | 201 | 62 | 2 | 0 | 1 | 2 | 15 | post | positive | positive | | negative |
| 757 | live | 81 | 85 | 3 | 0 | 1 | 4 | 2 | post | positive | positive | | negative |
| 758 | live | 155 | 36 | 3 | 0 | 2 | 1 | 4 | pre | positive | positive | | negative |
| 759 | live | 97 | 45 | 2 | 0 | 0 | 2 | 28 | pre | positive | positive | | negative |
| 760 | live | 75 | 86 | 3 | 0 | 1 | 4 | 0 | post | positive | negative | | NA |
| 761 | live | 352 | 76 | 3 | NA | 1 | 3 | 7 | post | positive | positive | | positive |
| 762 | live | 326 | 55 | 2 | NA | 1 | 2 | 50 | post | negative | negative | | NA |
| 763 | live | 319 | 34 | 2 | NA | 1 | 2 | 189 | pre | positive | positive | | negative |
| 764 | live | 218 | 57 | 1 | 0 | 0 | 1 | 205 | post | positive | positive | | negative |
| 765 | live | 242 | 56 | 2 | 0 | 1 | 2 | 75 | post | positive | positive | | negative |
| 766 | live | 177 | 69 | 2 | 0 | 0 | 2 | 148 | post | negative | negative | | negative |
| 767 | live | 168 | 56 | 2 | 0 | 1 | 2 | 24 | post | positive | positive | | negative |
| 768 | live | 50 | 48 | 3 | 0 | 3 | 2 | 82 | NA | positive | positive | | positive |
| 769 | live | 193 | 61 | 2 | 0 | 1 | 1 | 56 | post | negative | negative | | positive |
| 770 | live | 150 | 56 | 1 | 0 | 0 | 1 | 65 | post | positive | positive | | negative |
| 771 | live | 147 | 84 | 2 | 0 | 0 | 2 | 6 | post | positive | positive | | negative |
| 772 | live | 118 | 53 | 2 | 0 | 0 | 2 | 81 | post | negative | positive | | negative |
| 773 | live | 81 | 74 | 2 | NA | 0 | 2 | 17 | post | positive | positive | | positive |
| 774 | live | 121 | 81 | 3 | NA | 2 | 1 | 105 | post | positive | negative | | negative |
| 775 | live | 167 | 65 | 3 | NA | 2 | 1 | 207 | post | positive | positive | | negative |
| 776 | live | 116 | 80 | 3 | NA | 1 | 3 | 36 | post | positive | positive | | negative |
| 777 | live | 73 | 71 | 2 | NA | 0 | 2 | 482 | post | positive | positive | | negative |
| 778 | live | 125 | 61 | 3 | NA | 2 | 2 | 31 | post | positive | positive | | negative |
| 779 | live | 182 | 66 | 1 | 0 | 0 | 1 | 77 | post | positive | positive | | negative |
| 780 | live | 106 | 40 | 2 | 0 | 0 | 2 | 31 | pre | negative | negative | | negative |
| 781 | live | 210 | 75 | 2 | 0 | 0 | 2 | 159 | post | positive | positive | | negative |
| 782 | live | 19 | 72 | 2 | 0 | 0 | 2 | 17 | post | negative | negative | | NA |
| 783 | live | 186 | 58 | 3 | 0 | 2 | 1 | 381 | post | positive | positive | | negative |
| 784 | live | 119 | 47 | 2 | 0 | 1 | 2 | 170 | post | positive | positive | | negative |
| 785 | live | 126 | 49 | 3 | 0 | 2 | 1 | 169 | post | positive | positive | | negative |
| 786 | live | 145 | 59 | 1 | 0 | 0 | 1 | 206 | post | negative | negative | | NA |
| 787 | live | 146 | 36 | 3 | 0 | 2 | 2 | 116 | pre | positive | positive | | positive |
| 788 | live | 23 | 64 | 1 | 0 | 0 | 1 | 24 | post | positive | positive | | negative |
| 789 | live | 29 | 41 | 3 | 0 | 3 | 2 | 224 | post | positive | positive | | negative |
| 790 | live | 136 | 53 | 3 | 0 | 3 | 2 | 170 | post | positive | positive | | negative |
| 791 | live | 73 | 62 | 2 | 0 | 0 | 2 | 254 | post | positive | positive | | negative |
| 792 | live | 15 | 55 | 3 | 0 | 0 | 4 | 275 | post | positive | positive | | positive |
| 793 | live | 441 | 54 | 3 | 0 | 3 | 2 | 46 | post | positive | positive | | NA |
| 794 | live | 185 | 56 | 3 | 0 | 3 | 3 | 9 | post | positive | positive | | NA |
| 795 | live | 223 | 68 | 1 | 0 | 0 | 1 | 103 | post | positive | positive | | negative |
| 796 | live | 492 | 88 | 2 | 0 | 0 | 2 | 227 | post | positive | positive | | negative |
| 797 | live | 244 | 79 | 3 | NA | 1 | 3 | 29 | post | positive | negative | | negative |
| 798 | live | 368 | 53 | 2 | 0 | 0 | 2 | 28 | post | positive | positive | | negative |
| 799 | live | 1163 | 79 | 2 | 0 | 0 | 2 | 24 | post | positive | positive | | NA |
| 800 | live | 1555 | 34 | 1 | 0 | 1 | 1 | 4 | pre | positive | positive | | negative |
| 801 | live | 763 | 54 | 3 | 0 | 2 | 3 | 112 | post | positive | negative | | negative |
| 802 | live | 124 | 64 | 3 | 0 | 2 | 2 | 400 | post | positive | positive | | negative |
| 803 | live | 1172 | 64 | 2 | 0 | 0 | 2 | 53 | post | positive | negative | | negative |
| 804 | live | 352 | 41 | 2 | 0 | 0 | 3 | 17 | pre | positive | positive | | negative |
| 805 | live | 748 | 67 | 2 | 0 | 1 | 2 | 104 | post | positive | positive | | negative |
| 806 | live | 854 | 54 | 2 | 0 | 1 | 2 | 211 | post | positive | positive | | negative |
| 807 | live | 418 | 64 | 2 | 0 | 1 | 1 | 80 | post | positive | positive | | negative |
| 808 | live | 360 | 47 | 2 | 0 | 0 | 2 | 155 | pre | positive | positive | | negative |
| 809 | live | 1350 | 37 | 2 | 0 | 1 | 2 | 91 | pre | negative | negative | | NA |
| 810 | live | 1173 | 76 | 3 | 0 | 1 | 3 | 11 | post | positive | positive | | negative |
| 811 | live | 487 | 79 | 3 | 0 | 3 | 2 | 148 | post | negative | negative | | positive |
| 812 | live | 974 | 50 | 2 | 0 | 1 | 2 | 166 | pre | positive | positive | | negative |
| 813 | live | 845 | 62 | 2 | 0 | 0 | 2 | 128 | post | negative | negative | | negative |
| 814 | live | 834 | 65 | 1 | 0 | 0 | 1 | 59 | post | positive | positive | | negative |
| 815 | live | 890 | 52 | 2 | 0 | 0 | 2 | 67 | post | positive | positive | | negative |
| 816 | live | 838 | 74 | 1 | 0 | 0 | 1 | 50 | post | positive | positive | | negative |
| 817 | live | 748 | 53 | 2 | 0 | 1 | 2 | 52 | post | positive | positive | | positive |
| 818 | live | 834 | 78 | 2 | 0 | 0 | 2 | 1 | NA | positive | positive | | positive |
| 819 | live | 692 | 55 | 3 | 0 | 2 | 2 | 400 | post | negative | negative | | negative |
| 820 | live | 688 | 35 | 2 | 0 | 0 | 2 | 26 | pre | positive | positive | | positive |
| 821 | live | 518 | 64 | 1 | 0 | 0 | 1 | 143 | post | positive | positive | | negative |
| 822 | live | 591 | 48 | 2 | 0 | 0 | 2 | 77 | post | negative | negative | | NA |
| 823 | live | 588 | 56 | 1 | 0 | 0 | 1 | 12 | post | positive | negative | | positive |
| 824 | live | 586 | 51 | 2 | 0 | 1 | 2 | 54 | pre | positive | positive | | negative |
| 825 | live | 325 | 68 | 1 | 0 | 0 | 1 | 2 | post | positive | positive | | negative |
| 826 | live | 553 | 58 | 2 | 0 | 1 | 2 | 32 | post | positive | negative | | NA |
| 827 | live | 481 | 61 | 1 | 0 | 0 | 1 | 1 | post | positive | positive | | NA |
| 828 | live | 450 | 43 | 2 | 0 | 1 | 1 | 24 | pre | negative | negative | | negative |
| 829 | live | 515 | 50 | 2 | 0 | 0 | 2 | 280 | pre | negative | negative | | negative |
| 830 | live | 502 | 45 | 3 | 0 | 3 | 2 | 154 | pre | positive | positive | | negative |
| 831 | live | 497 | 61 | 1 | 0 | 0 | 1 | 103 | post | positive | positive | | negative |
| 832 | live | 316 | 47 | 2 | 0 | 0 | 2 | 71 | pre | positive | positive | | negative |
| 833 | live | 518 | 40 | 2 | 0 | 1 | 1 | 16 | pre | positive | positive | | positive |
| 834 | live | 469 | 64 | 1 | 0 | 0 | 1 | 47 | post | positive | positive | | negative |
| 835 | live | 316 | 76 | 2 | 0 | 0 | 2 | 11 | post | positive | positive | | negative |
| 836 | live | 42 | 38 | 2 | 0 | 1 | 1 | 43 | pre | positive | positive | | positive |
| 837 | live | 411 | 44 | 2 | 0 | 0 | 2 | 77 | pre | positive | positive | | negative |
| 838 | live | 464 | 51 | 1 | 0 | 0 | 1 | 10 | pre | positive | positive | | negative |
| 839 | live | 34 | 58 | 2 | 0 | 1 | 2 | 3 | post | positive | positive | | negative |
| 840 | live | 331 | 65 | 2 | 0 | 0 | 2 | 3 | post | positive | positive | | negative |
| 841 | live | 235 | 66 | 2 | 0 | 0 | 2 | 150 | post | positive | positive | | NA |
| 842 | live | 396 | 89 | 1 | 0 | NA | 1 | 48 | post | positive | positive | | negative |
| 843 | live | 315 | 61 | 1 | 0 | 0 | 1 | 50 | post | positive | positive | | negative |
| 844 | live | 330 | 64 | 2 | 0 | 1 | 1 | 6 | post | positive | positive | | negative |
| 845 | live | 274 | 34 | 2 | 0 | 1 | 2 | 39 | post | positive | negative | | negative |
| 846 | live | 267 | 65 | 2 | 0 | 0 | 2 | 38 | post | positive | positive | | negative |
| 847 | live | 1965 | 63 | 2 | 0 | 1 | 2 | 69 | post | negative | negative | | negative |
| 848 | live | 1271 | 50 | 3 | 0 | 2 | 2 | 234 | post | negative | negative | | positive |
| 849 | live | 1361 | 45 | 2 | 0 | 1 | 2 | 968 | post | positive | positive | | positive |
| 850 | live | 911 | 74 | 3 | 0 | 2 | 1 | 3 | post | positive | positive | | negative |
| 851 | live | 774 | 46 | 2 | 0 | 0 | 2 | 1287 | pre | positive | positive | | NA |
| 852 | live | 338 | 44 | 2 | 0 | 0 | 2 | 967 | pre | negative | negative | | negative |
| 853 | live | 297 | 63 | 1 | 0 | 0 | 1 | 11 | post | positive | positive | | NA |
| 854 | live | 317 | 53 | 2 | 0 | 0 | 2 | 49 | post | positive | positive | | negative |
| 855 | live | 983 | 61 | 2 | 0 | 1 | 2 | 39 | post | positive | positive | | negative |
| 856 | live | 956 | 74 | 1 | 0 | 0 | 1 | 116 | post | positive | positive | | negative |
| 857 | live | 755 | 45 | 2 | 0 | 1 | 2 | 25 | pre | positive | positive | | NA |
| 858 | live | 658 | 80 | 1 | 0 | 0 | 1 | 844 | post | positive | positive | | NA |
| 859 | live | 850 | 51 | 1 | 0 | 0 | 1 | 252 | post | negative | positive | | negative |
| 860 | live | 725 | 57 | 1 | 0 | 0 | 1 | 127 | post | positive | positive | | negative |
| 861 | live | 519 | 71 | 2 | 0 | 1 | 1 | 117 | post | positive | positive | | NA |
| 862 | live | 14 | 78 | 2 | 0 | 1 | 1 | 11 | post | positive | positive | | NA |
| 863 | live | 392 | 60 | 1 | 0 | 0 | 1 | 5 | post | positive | positive | | negative |
| 864 | live | 498 | 37 | 1 | 0 | 0 | 1 | 165 | pre | positive | positive | | negative |
| 865 | live | 127 | 60 | 1 | 0 | 0 | 1 | 56 | post | positive | positive | | NA |
| 866 | live | 1312 | 44 | 2 | 0 | 1 | 1 | 95 | pre | positive | positive | | negative |
| 867 | live | 633 | 40 | 3 | 0 | 2 | 2 | 113 | pre | negative | negative | | NA |
| 868 | live | 1015 | 52 | 2 | 0 | 1 | 2 | 68 | post | positive | positive | | negative |
| 869 | live | 388 | 40 | 2 | 0 | 1 | 1 | 72 | pre | positive | positive | | NA |
| 870 | live | 463 | 59 | 2 | 0 | 1 | 1 | 138 | post | positive | positive | | negative |
| 871 | live | 972 | 41 | 2 | 0 | 1 | 2 | 32 | pre | negative | negative | | positive |
| 872 | dead | 879 | 71 | 3 | 0 | 3 | 2 | 128 | post | negative | negative | | positive |
| 873 | live | 350 | 50 | 2 | 0 | 0 | 2 | 134 | post | negative | negative | | negative |
| 874 | live | 2876 | 59 | 1 | 0 | 0 | 1 | 92 | post | negative | negative | | NA |
| 875 | live | 2750 | 57 | 2 | 0 | 1 | 2 | 109 | post | negative | negative | | negative |
| 876 | dead | 266 | 84 | 3 | 0 | 3 | 4 | 0 | post | negative | negative | | negative |
| 877 | live | 1014 | 73 | 3 | NA | 2 | 3 | 4 | post | negative | negative | | negative |
| 878 | live | 239 | 46 | 1 | 0 | 0 | 1 | 29 | pre | negative | negative | | negative |
| 879 | live | 326 | 78 | 3 | 0 | 3 | 2 | 58 | post | positive | positive | | negative |
| 880 | live | 168 | 77 | 2 | 0 | 1 | 2 | 60 | post | positive | positive | | negative |
| 881 | live | 217 | 43 | 3 | 0 | 3 | 2 | 413 | pre | positive | positive | | positive |
| 882 | live | 219 | 69 | 2 | 0 | 1 | 2 | 4 | post | positive | positive | | negative |
| 883 | live | 549 | 47 | 1 | 0 | 1 | 1 | 18 | pre | positive | positive | | negative |
| 884 | live | 831 | 72 | 3 | 0 | 2 | 2 | 5 | post | positive | positive | | negative |
| 885 | live | 677 | 48 | 1 | 0 | 0 | 1 | 127 | post | negative | negative | | negative |
| 886 | live | 605 | 44 | 2 | 0 | 0 | 2 | 0 | pre | negative | negative | | negative |
| 887 | live | 578 | 69 | 1 | NA | 0 | 1 | 121 | post | positive | positive | | negative |
| 888 | live | 280 | 90 | 3 | NA | 3 | 2 | 1 | post | positive | negative | | negative |
| 889 | live | 0 | 70 | 3 | 0 | 2 | 1 | 27 | post | NA | NA | | NA |
| 890 | live | 5 | 41 | 3 | 0 | 2 | 2 | 213 | pre | positive | positive | | NA |
| 891 | live | 0 | 45 | 2 | 0 | 1 | 2 | 24 | post | positive | positive | | NA |
| 892 | live | 0 | 52 | 2 | 0 | 1 | 2 | 6 | post | positive | positive | | NA |
| 893 | live | 0 | 48 | 2 | 0 | 0 | 2 | 9 | post | negative | NA | | NA |
| 894 | live | 0 | 58 | 2 | 0 | 0 | 2 | 83 | post | negative | positive | | NA |
| 895 | live | 0 | 58 | 2 | 0 | 0 | 2 | 7 | post | positive | positive | | NA |
| 896 | live | 0 | 61 | 2 | 0 | 1 | 2 | 101 | post | negative | positive | | NA |
| 897 | live | 0 | 75 | 2 | 0 | 1 | 2 | 135 | post | negative | negative | | NA |
| 898 | live | 0 | 28 | 2 | 0 | 1 | 2 | 732 | pre | positive | positive | | NA |
| 899 | live | 0 | 60 | 2 | 0 | 0 | 2 | 36 | post | positive | positive | | NA |
| 900 | live | 0 | 62 | 2 | 0 | 0 | 2 | 82 | post | positive | positive | | NA |
| 901 | live | 0 | 71 | 2 | 0 | 1 | 2 | 33 | post | positive | positive | | NA |
| 902 | live | -7 | 51 | 2 | 0 | 0 | 2 | 5 | post | positive | positive | | NA |
| 903 | live | 0 | 61 | 2 | 0 | 0 | 2 | 563 | post | NA | NA | | NA |
| 904 | live | 0 | 58 | 2 | 0 | 1 | 1 | 312 | post | NA | NA | | NA |
| 905 | live | 19 | 51 | 3 | NA | 3 | 2 | 84 | post | positive | negative | | negative |
| 906 | live | 64 | 70 | 3 | 0 | 3 | 2 | 56 | post | NA | NA | | NA |
| 907 | live | 29 | 66 | 1 | 0 | 0 | 1 | 89 | post | NA | NA | | NA |
| 908 | live | 42 | 63 | 1 | 0 | 0 | 1 | 30 | post | NA | NA | | NA |
| 909 | live | 84 | 63 | 2 | 0 | 0 | 2 | 2 | post | NA | NA | | NA |
| 910 | live | 34 | 64 | 2 | 0 | 0 | 2 | 36 | post | NA | NA | | NA |
| 911 | live | 29 | 48 | 1 | 0 | 0 | 1 | 85 | pre | NA | NA | | NA |
| 912 | live | 21 | 40 | 2 | 0 | 0 | 2 | 28 | pre | NA | NA | | NA |
| 913 | live | 1224 | 56 | 3 | 0 | 3 | 4 | 5 | post | NA | NA | | NA |
| 914 | live | 20 | 67 | 2 | 0 | 0 | 2 | 10 | post | NA | NA | | NA |
| 915 | live | 47 | 74 | 3 | 0 | 2 | 2 | 60 | post | NA | NA | | NA |
| 916 | live | 38 | 68 | 3 | 0 | 2 | 2 | 93 | post | NA | NA | | NA |
| 917 | live | 35 | 62 | 3 | 0 | 2 | 1 | 61 | post | NA | NA | | NA |
| 918 | live | 14 | 63 | 2 | 0 | 0 | 2 | 2 | post | NA | NA | | NA |
| 919 | live | 48 | 43 | 3 | 0 | 2 | 1 | 56 | pre | NA | NA | | NA |
| 920 | live | 16 | 45 | 3 | 0 | 2 | 2 | 0 | pre | NA | NA | | NA |
| 921 | live | 24 | 42 | 2 | 0 | 1 | 2 | 354 | pre | positive | positive | | NA |
| 922 | live | 0 | 58 | 2 | 0 | 1 | 2 | 27 | post | NA | NA | | NA |
| 923 | live | 0 | 37 | 1 | 0 | 0 | 1 | 85 | pre | NA | NA | | NA |
| 924 | live | 0 | 74 | 2 | 0 | 0 | 2 | 262 | post | positive | negative | | NA |
| 925 | live | 0 | 71 | 1 | 0 | 0 | 1 | 79 | post | positive | negative | | NA |
| 926 | live | 0 | 38 | 2 | 0 | 0 | 2 | 439 | pre | positive | positive | | NA |
| 927 | live | 0 | 56 | 3 | 0 | 2 | 2 | 203 | post | positive | positive | | NA |
| 928 | live | 0 | 47 | 2 | 0 | 0 | 2 | 26 | post | negative | negative | | NA |
| 929 | live | 0 | 42 | 2 | 0 | 1 | 2 | 70 | pre | positive | positive | | NA |
| 930 | live | 43 | 52 | 2 | 0 | 0 | 2 | 2041 | post | NA | NA | | NA |
| 931 | live | 21 | 54 | 2 | 0 | 0 | 2 | 69 | post | NA | NA | | NA |
| 932 | live | 26 | 47 | 2 | 0 | 1 | 2 | 7 | pre | NA | NA | | NA |
| 933 | live | 13 | 59 | 1 | 0 | 0 | 1 | 31 | post | NA | NA | | NA |
| 934 | live | 22 | 51 | 2 | 0 | 0 | 2 | 678 | post | NA | NA | | NA |
| 935 | live | 29 | 45 | 2 | 0 | 0 | 2 | 62 | pre | NA | NA | | NA |
| 936 | live | 11 | 69 | 2 | 0 | 1 | 1 | 42 | post | NA | NA | | NA |
| 937 | live | 22 | 71 | 2 | 0 | 0 | 2 | 46 | post | positive | positive | | negative |
| 938 | live | 13 | 72 | 2 | 0 | 1 | 2 | 19 | post | NA | NA | | NA |
| 939 | live | 23 | 63 | 2 | 0 | 0 | 2 | 538 | post | NA | NA | | NA |
| 940 | live | 19 | 49 | 2 | 0 | 0 | 2 | 140 | pre | NA | NA | | NA |
| 941 | live | 31 | 78 | 3 | 0 | 3 | 3 | 56 | post | positive | positive | | NA |
| 942 | live | 19 | 33 | 2 | 0 | 0 | 2 | 212 | pre | NA | NA | | NA |
| 943 | live | 23 | 48 | 2 | 0 | 1 | 2 | 283 | post | positive | positive | | NA |
| 944 | live | 0 | 85 | 1 | 0 | 0 | 1 | 0 | post | positive | positive | | NA |
| 945 | live | 7 | 63 | 3 | 0 | 2 | 2 | 9 | post | positive | positive | | negative |
| 946 | live | 12 | 60 | 3 | 0 | 3 | 2 | 536 | post | positive | positive | | NA |
| 947 | live | 8 | 65 | 2 | 0 | 0 | 3 | 28 | post | negative | negative | | NA |
| 948 | live | 7 | 41 | 2 | 0 | 0 | 2 | 4 | pre | NA | NA | | NA |
| 949 | live | 0 | 63 | 2 | 0 | 0 | 2 | 6 | post | NA | NA | | NA |
| 950 | live | 92 | 45 | 3 | NA | 3 | 3 | 164 | pre | positive | positive | | negative |
| 951 | live | 252 | 80 | 2 | NA | 1 | 2 | 161 | post | positive | positive | | positive |
| 952 | live | 1082 | 48 | 2 | NA | 1 | 1 | 47 | post | positive | positive | | NA |
| 953 | live | 258 | 38 | 1 | NA | 0 | 1 | 615 | pre | positive | positive | | NA |
| 954 | live | 260 | 53 | 3 | NA | 2 | 2 | 224 | post | positive | positive | | NA |
| 955 | live | 282 | 38 | 2 | 0 | 1 | 2 | 77 | pre | positive | positive | | NA |
| 956 | live | 140 | 50 | NA | NA | 1 | 1 | 242 | pre | positive | positive | | NA |
| 957 | live | 252 | 61 | 1 | 0 | 0 | 1 | 68 | post | positive | positive | | positive |
| 958 | live | 227 | 59 | 2 | 0 | 0 | 3 | 137 | post | positive | positive | | NA |
| 959 | live | 595 | 70 | 1 | 0 | 0 | 1 | 86 | post | positive | positive | | NA |
| 960 | live | 523 | 56 | 2 | NA | 1 | 2 | 338 | post | negative | negative | | negative |
| 961 | live | 464 | 58 | 2 | NA | 0 | 2 | 26 | post | negative | negative | | negative |
| 962 | live | 562 | 43 | 2 | NA | 0 | 2 | 1 | pre | positive | positive | | NA |
| 963 | live | 593 | 63 | 2 | NA | 0 | 2 | 30 | post | positive | positive | | negative |
| 964 | live | 941 | 56 | 2 | 0 | 0 | 2 | 56 | post | positive | negative | | positive |
| 965 | live | 1121 | 55 | 2 | NA | 1 | 2 | 77 | post | positive | negative | | NA |
| 966 | live | 920 | 68 | 3 | NA | 3 | 2 | 192 | post | negative | negative | | negative |
| 967 | live | 987 | 48 | 2 | NA | 0 | 2 | 65 | pre | positive | positive | | NA |
| 968 | live | 501 | 43 | 2 | 0 | 0 | 2 | 184 | pre | negative | negative | | NA |
| 969 | live | 365 | 77 | 2 | 0 | 1 | 2 | 13 | post | positive | positive | | negative |
| 970 | live | 312 | 64 | 2 | 0 | 1 | 2 | 31 | post | positive | positive | | NA |
| 971 | live | 635 | 59 | 2 | 0 | 0 | 2 | 398 | post | negative | negative | | negative |
| 972 | dead | 239 | 58 | 3 | 0 | 3 | 2 | 51 | post | negative | negative | | NA |
| 973 | live | 267 | 59 | 2 | 0 | 1 | 2 | 52 | post | positive | positive | | negative |
| 974 | live | 608 | 70 | 3 | NA | 1 | 3 | 97 | post | negative | negative | | NA |
| 975 | live | 187 | 66 | 2 | NA | 0 | 3 | 7 | post | positive | positive | | negative |
| 976 | live | 175 | 61 | 2 | 0 | 1 | 1 | 10 | NA | positive | positive | | positive |
| 977 | live | 107 | 56 | 2 | 0 | 1 | 1 | 45 | post | positive | positive | | NA |
| 978 | live | 140 | 50 | 1 | 0 | 0 | 1 | 91 | post | positive | positive | | NA |
| 979 | live | 876 | 53 | 2 | NA | 1 | 2 | 18 | post | positive | positive | | negative |
| 980 | live | 140 | 52 | 2 | 0 | 1 | 1 | 20 | post | negative | negative | | NA |
| 981 | live | 1120 | 59 | 3 | NA | 3 | 1 | 67 | post | negative | negative | | positive |
| 982 | live | 1173 | 41 | 2 | NA | 1 | 2 | 370 | pre | positive | negative | | negative |
| 983 | live | 239 | 39 | 3 | NA | 3 | 3 | 271 | pre | positive | positive | | negative |
| 984 | live | 191 | 52 | 2 | NA | 0 | 2 | 56 | post | positive | positive | | negative |
| 985 | live | 647 | 60 | 2 | NA | 0 | 3 | 298 | NA | positive | negative | | NA |
| 986 | live | 229 | 61 | 3 | 0 | 1 | 3 | 277 | post | negative | negative | | NA |
| 987 | live | 309 | 75 | 2 | NA | 0 | 2 | 91 | post | positive | positive | | NA |
| 988 | live | 322 | 51 | 3 | NA | 1 | 3 | 45 | NA | positive | positive | | NA |
| 989 | live | 463 | 34 | 2 | 0 | 1 | 1 | 2 | NA | positive | positive | | positive |
| 990 | live | 510 | 59 | 2 | NA | 0 | 2 | 1 | NA | positive | negative | | NA |
| 991 | live | 760 | 62 | 2 | 0 | 0 | 2 | 282 | NA | negative | negative | | NA |
| 992 | live | 952 | 60 | 2 | 0 | 0 | 2 | 98 | NA | positive | positive | | NA |
| 993 | live | 1010 | 32 | 2 | 0 | 1 | 2 | 35 | NA | negative | negative | | positive |
| 994 | live | 225 | 63 | 3 | NA | 0 | 4 | 15 | NA | positive | positive | | negative |
| 995 | live | 711 | 58 | 2 | NA | 0 | 3 | 28 | post | negative | negative | | NA |
| 996 | dead | 1812 | 69 | 1 | 0 | 0 | 1 | 29 | post | positive | positive | | negative |
| 997 | live | 5909 | 46 | 2 | 0 | 1 | 2 | 131 | post | positive | positive | | positive |
| 998 | live | 1616 | 62 | 2 | 0 | 0 | 2 | 95 | post | negative | negative | | negative |
| 999 | live | 1628 | 57 | 2 | 0 | 1 | 1 | 16 | post | positive | positive | | negative |
| 1000 | live | 1309 | 53 | 1 | 0 | 0 | 1 | 287 | post | negative | negative | | negative |
| 1001 | live | 1299 | 53 | 2 | 0 | 1 | 1 | 256 | post | negative | negative | | negative |
| 1002 | live | 1286 | 58 | 1 | 0 | 0 | 1 | 267 | post | negative | negative | | negative |
| 1003 | live | 1883 | 52 | 1 | 0 | 0 | 1 | 366 | pre | negative | negative | | negative |
| 1004 | live | 1896 | 58 | 1 | 0 | 0 | 1 | 244 | post | positive | negative | | NA |
| 1005 | live | 2763 | 50 | 1 | 0 | 0 | 1 | 55 | post | positive | positive | | negative |
| 1006 | live | 2331 | 57 | 2 | 0 | 0 | 2 | 39 | post | positive | positive | | negative |
| 1007 | live | 2352 | 58 | 2 | 0 | 0 | 2 | 307 | post | positive | positive | | negative |
| 1008 | live | 1623 | 54 | 1 | 0 | 0 | 1 | 362 | post | positive | positive | | NA |
| 1009 | live | 2982 | 63 | 2 | 0 | 0 | 2 | 67 | post | negative | positive | | negative |
| 1010 | live | 743 | 72 | 2 | 0 | 1 | 2 | 73 | post | positive | positive | | NA |
| 1011 | live | 982 | 46 | 3 | 0 | 1 | 3 | 77 | pre | positive | positive | | negative |
| 1012 | live | 1717 | 49 | 2 | 0 | 0 | 2 | 100 | post | negative | negative | | negative |
| 1013 | live | 1641 | 44 | 3 | 0 | 2 | 1 | 178 | post | positive | positive | | negative |
| 1014 | live | 1753 | 67 | 3 | 0 | 3 | 3 | 107 | post | positive | positive | | negative |
| 1015 | live | 412 | 63 | 2 | 0 | 1 | 2 | 84 | post | positive | positive | | negative |
| 1016 | live | 551 | 65 | 2 | 0 | 1 | 2 | 308 | post | positive | positive | | negative |
| 1017 | live | 79 | 56 | 2 | 0 | 0 | 2 | 67 | post | negative | negative | | NA |
| 1018 | live | 1849 | 45 | 2 | 0 | 1 | 2 | 53 | pre | positive | positive | | negative |
| 1019 | live | 360 | 49 | 2 | 0 | 1 | 2 | 114 | post | positive | positive | | negative |
| 1020 | live | 352 | 46 | 2 | 0 | 0 | 2 | 35 | post | positive | positive | | positive |
| 1021 | live | 252 | 44 | 2 | NA | 1 | 2 | 434 | NA | positive | positive | | NA |
| 1022 | live | 203 | 79 | 3 | 0 | 3 | 3 | 102 | NA | positive | negative | | NA |
| 1023 | live | 124 | 52 | 3 | 0 | 3 | 2 | 312 | pre | positive | positive | | NA |
| 1024 | live | 140 | 54 | 2 | 0 | 1 | 2 | 114 | post | positive | positive | | NA |
| 1025 | live | 266 | 73 | 1 | 0 | 0 | 1 | 140 | post | negative | negative | | positive |
| 1026 | live | 20 | 61 | 1 | NA | 0 | 1 | 245 | post | positive | positive | | negative |
| 1027 | live | 91 | 62 | 1 | NA | 0 | 1 | 339 | post | negative | negative | | negative |
| 1028 | live | 109 | 56 | 2 | NA | 0 | 2 | 10 | post | positive | positive | | negative |
| 1029 | live | 210 | 84 | 2 | NA | 0 | 2 | 33 | post | positive | positive | | negative |
| 1030 | live | 169 | 64 | 2 | NA | 1 | 2 | 93 | post | positive | negative | | positive |
| 1031 | live | 143 | 88 | 3 | 0 | NA | 4 | 34 | post | positive | positive | | positive |
| 1032 | live | 111 | 46 | 2 | 0 | 0 | 2 | 254 | post | positive | positive | | positive |
| 1033 | live | 97 | 50 | 1 | NA | 0 | 1 | 437 | pre | negative | negative | | negative |
| 1034 | live | 99 | 49 | 2 | 0 | 1 | 2 | 69 | post | positive | negative | | negative |
| 1035 | live | 352 | 90 | 2 | NA | NA | 2 | 5 | post | positive | positive | | negative |
| 1036 | live | 80 | 77 | 3 | NA | 2 | 2 | 22 | post | positive | positive | | negative |
| 1037 | live | 118 | 50 | 2 | NA | 0 | 2 | 0 | post | negative | positive | | positive |
| 1038 | live | 126 | 67 | 1 | NA | 0 | 1 | 297 | post | negative | negative | | positive |
| 1039 | live | 137 | 55 | 4 | 1 | NA | 1 | 175 | post | positive | positive | | negative |
| 1040 | live | 137 | 61 | 1 | NA | 0 | 1 | 38 | post | negative | negative | | negative |
| 1041 | live | 170 | 49 | 2 | NA | 1 | 2 | 162 | pre | positive | positive | | negative |
| 1042 | live | 98 | 70 | 2 | 0 | 1 | 2 | 22 | post | positive | positive | | positive |
| 1043 | live | 189 | 61 | 2 | NA | 0 | 2 | 57 | post | positive | negative | | negative |
| 1044 | live | 224 | 69 | 3 | NA | 3 | 3 | 58 | post | positive | positive | | positive |
| 1045 | live | 484 | 73 | 3 | NA | 1 | 3 | 16 | post | positive | positive | | negative |
| 1046 | live | 85 | 44 | 2 | 0 | 1 | 2 | 112 | NA | positive | positive | | negative |
| 1047 | live | 64 | 59 | 2 | NA | 1 | 2 | 401 | NA | positive | positive | | NA |
| 1048 | live | 385 | 71 | 2 | NA | 0 | 2 | 62 | post | negative | negative | | negative |
| 1049 | live | 1416 | 70 | 2 | NA | 0 | 2 | 45 | post | negative | negative | | negative |
| 1050 | live | 229 | 40 | 2 | NA | 0 | 3 | 15 | pre | positive | positive | | negative |
| 1051 | live | 1357 | 61 | 2 | NA | 0 | 2 | 37 | post | positive | positive | | negative |
| 1052 | live | 841 | 63 | 2 | NA | 1 | 1 | 251 | post | positive | positive | | negative |
| 1053 | live | 733 | 43 | 3 | NA | 3 | 2 | 36 | pre | positive | positive | | negative |
| 1054 | live | 797 | 40 | 2 | NA | 1 | 1 | 12 | pre | negative | negative | | negative |
| 1055 | live | 513 | 51 | 1 | NA | 0 | 1 | 172 | pre | positive | positive | | negative |
| 1056 | live | 429 | 52 | 2 | NA | 1 | 1 | 503 | post | positive | negative | | positive |
| 1057 | live | 313 | 57 | 1 | NA | 0 | 1 | 22 | post | positive | negative | | positive |
| 1058 | live | 298 | 66 | 2 | NA | 1 | 2 | 116 | post | positive | negative | | positive |
| 1059 | live | 413 | 74 | 1 | NA | 1 | 1 | 129 | post | positive | positive | | negative |
| 1060 | live | 714 | 36 | 2 | NA | 1 | 1 | 445 | NA | negative | negative | | negative |
| 1061 | live | 1658 | 80 | 1 | NA | 0 | 1 | 48 | post | positive | positive | | negative |
| 1062 | live | 1021 | 72 | 2 | NA | 0 | 2 | 23 | post | positive | positive | | negative |
| 1063 | live | 945 | 71 | 1 | NA | 0 | 1 | 377 | post | positive | positive | | negative |
| 1064 | live | 413 | 59 | 3 | NA | 3 | 3 | 174 | post | positive | negative | | negative |
| 1065 | live | 220 | 39 | 2 | NA | 1 | 2 | 12 | pre | positive | positive | | negative |
| 1066 | live | 58 | 75 | 2 | NA | 0 | 2 | 765 | NA | negative | negative | | negative |
| 1067 | live | 480 | 43 | 1 | NA | 0 | 1 | 81 | NA | negative | negative | | negative |
| 1068 | live | 229 | 49 | 2 | NA | 0 | 2 | 67 | pre | positive | positive | | negative |
| 1069 | live | 848 | 48 | 1 | NA | 0 | 1 | 38 | NA | positive | positive | | negative |
| 1070 | live | 195 | 67 | 2 | 0 | 0 | 3 | 104 | post | negative | negative | | negative |
| 1071 | dead | 1430 | 72 | 3 | 0 | 2 | 2 | 75 | post | positive | positive | | NA |
| 1072 | live | 1953 | 64 | 2 | 0 | 0 | 2 | 122 | post | positive | negative | | positive |
| 1073 | live | 1855 | 41 | 2 | 0 | 0 | 2 | 316 | pre | positive | positive | | NA |
| 1074 | live | -7 | 54 | 3 | 0 | 0 | 4 | 83 | NA | NA | NA | | NA |
| 1075 | live | 5 | 35 | 4 | 1 | 1 | 4 | 5 | pre | NA | NA | | NA |
| 1076 | live | 8 | 30 | 2 | 0 | 0 | 3 | 99 | pre | NA | NA | | NA |
| 1077 | live | 120 | 29 | 3 | 0 | 2 | 4 | 142 | pre | NA | NA | | NA |
| 1078 | live | 212 | 64 | 2 | 0 | 0 | 2 | 62 | post | positive | positive | | negative |
| 1079 | live | 207 | 71 | 2 | 0 | 1 | 2 | 157 | post | positive | positive | | NA |
| 1080 | live | 263 | 29 | 3 | NA | 3 | 3 | 53 | NA | positive | positive | | negative |
| 1081 | live | 322 | 63 | 2 | 0 | 1 | 2 | 118 | post | positive | positive | | negative |
| 1082 | live | 241 | 65 | 2 | 0 | 0 | 2 | 105 | post | negative | negative | | negative |
| 1083 | live | 169 | 67 | 2 | 0 | 0 | 2 | 10 | post | positive | positive | | negative |
| 1084 | live | 259 | 82 | 3 | NA | 1 | 3 | 7 | post | positive | negative | | negative |
| 1085 | live | 218 | 47 | 1 | 0 | 0 | 1 | 49 | post | positive | positive | | positive |
| 1086 | live | 209 | 51 | 2 | 0 | 1 | 2 | 273 | post | negative | negative | | NA |
| 1087 | live | 119 | 64 | 2 | 0 | 1 | 2 | 227 | post | positive | positive | | negative |
| 1088 | live | 426 | 73 | 2 | NA | 0 | 2 | 8 | post | positive | positive | | NA |
| 1089 | dead | 116 | 63 | 4 | 1 | 3 | 4 | 0 | post | negative | negative | | positive |
| 1090 | live | 1590 | 75 | 3 | 0 | 2 | 1 | 21 | post | positive | positive | | negative |
| 1091 | live | 161 | 66 | 2 | NA | 0 | 2 | 10 | post | positive | positive | | negative |
| 1092 | live | 1550 | 55 | 2 | NA | 1 | 2 | 153 | post | positive | positive | | NA |
| 1093 | live | 791 | 77 | 1 | NA | 0 | 1 | 24 | post | positive | positive | | negative |
| 1094 | live | 292 | 46 | 3 | NA | 2 | 1 | 227 | post | positive | positive | | NA |
| 1095 | live | 278 | 68 | 2 | NA | 0 | 3 | 129 | post | positive | positive | | NA |
| 1096 | live | 3042 | 61 | 3 | NA | 1 | 3 | 283 | post | positive | positive | | NA |
| 1097 | live | 2800 | 46 | 1 | 0 | 0 | 1 | 29 | pre | positive | positive | | negative |

| **Table S2. Clinicopathological information of 20 patients enrolled from our breast center** | | | | | | | | | | |
| --- | --- | --- | --- | --- | --- | --- | --- | --- | --- | --- |
| patient ID | age | stage | grade | tumor extension (cm) | T | N | M | ER | PR | HER2 |
| 289421 | 33 | ⅡA | 2 | 2.3 | 2 | 0 | 0 | >95%1+ | >90%1+~2+ | + |
| 097434 | 45 | ⅡB | 3 | 2.1 | 2 | 1a | 0 | - | - | - |
| 289402 | 78 | ⅡA | 2 | 3.5 | 2 | 0 | 0 | 90%3+ | 90%2+ | - |
| 289506 | 65 | ⅡB | 3 | 4.5 | 2 | 1a | 0 | >95%3+ | >95%2+ | - |
| 289243 | 50 | ⅡA | 2 | 2.3 | 2 | 0 | 0 | 85%2+~3+ | 70%2+ | + |
| 289304 | 47 | ⅠA | 1 | 2.1 | 1c | 0 | 0 | >95%2+~3+ | >95%3+ | - |
| 289462 | 57 | ⅢA | 3 | 2.1 | 2 | 2a | 0 | >95%3+ | 80%1+ | - |
| 289520 | 78 | ⅡA | 3 | 2.5 | 2 | 0 | 0 | 70%1+ | - | + |
| 289481 | 52 | ⅡA | 3 | 3 | 2 | 0 | 0 | >95%3+ | 2%1+ | + |
| 289381 | 65 | ⅡB | 3 | 2.7 | 2 | 1a | 0 | 90%3+ | 25%2+~3+ | - |
| 289522 | 56 | ⅡA | 3 | 3.2 | 2 | 0 | 0 | - | - | - |
| 289568 | 53 | ⅡB | 2 | 2.2 | 2 | 1a | 0 | >95%3+ | 60%1+~2+ | - |
| 289654 | 39 | ⅢC | 3 | 4.5 | 2 | 3a | 0 | >95%3+ | 90%2+ | - |
| 289631 | 48 | ⅡA | 3 | 2.5 | 2 | 0 | 0 | 95% 2~3+ | 85% 2+ | - |
| 289610 | 37 | ⅡB | 3 | 2.5 | 2 | 1c | 0 | 30% 2+ | 10% 1+ | - |
| 289671 | 44 | ⅠA | 2 | 1.6 | 1c | 0 | 0 | >95% 2~3+ | 80% 1+ | - |
| 289676 | 57 | ⅡA | 3 | 2.1 | 2 | 0 | 0 | - | - | + |
| 289823 | 63 | ⅠA | 3 | 2 | 1c | 0 | 0 | >90% 2+~3+ | - | - |
| 289759 | 52 | ⅢA | 3 | 5.8 | 3 | 1a | 0 | 2% 1+~2+ | - | - |
| 289760 | 64 | ⅡA | 3 | 2.5 | 2 | 0 |  | - | - | - |

**Table S3: Tripod checklist**

| **Section/Topic** | **Item** |  | **Checklist Item** | **Page** |
| --- | --- | --- | --- | --- |
| **Title and abstract** | | | | |
| Title | 1 | D;V | Identify the study as developing and/or validating a multivariable prediction model, the target population, and the outcome to be predicted. | 1 |
| Abstract | 2 | D;V | Provide a summary of objectives, study design, setting, participants, sample size, predictors, outcome, statistical analysis, results, and conclusions. | 1 |
| **Introduction** | | | | |
| Background and objectives | 3a | D;V | Explain the medical context (including whether diagnostic or prognostic) and rationale for developing or validating the multivariable prediction model, including references to existing models. | 2 |
|  | 3b | D;V | Specify the objectives, including whether the study describes the development or validation of the model or both. | 2 |
| **Methods** | | | | |
| Source of data | 4a | D;V | Describe the study design or source of data (e.g., randomized trial, cohort, or registry data), separately for the development and validation data sets, if applicable. | 2-3 |
|  | 4b | D;V | Specify the key study dates, including start of accrual; end of accrual; and, if applicable, end of follow-up. | 4 |
| Participants | 5a | D;V | Specify key elements of the study setting (e.g., primary care, secondary care, general population) including number and location of centres. | 2-4 |
|  | 5b | D;V | Describe eligibility criteria for participants. | 2-3 |
|  | 5c | D;V | Give details of treatments received, if relevant. | NA |
| Outcome | 6a | D;V | Clearly define the outcome that is predicted by the prediction model, including how and when assessed. | 3-4 |
|  | 6b | D;V | Report any actions to blind assessment of the outcome to be predicted. | NA |
| Predictors | 7a | D;V | Clearly define all predictors used in developing or validating the multivariable prediction model, including how and when they were measured. | 3 |
|  | 7b | D;V | Report any actions to blind assessment of predictors for the outcome and other predictors. | NA |
| Sample size | 8 | D;V | Explain how the study size was arrived at. | 2-3 |
| Missing data | 9 | D;V | Describe how missing data were handled (e.g., complete-case analysis, single imputation, multiple imputation) with details of any imputation method. | 2-3 |
| Statistical analysis methods | 10a | D | Describe how predictors were handled in the analyses. | 3-4 |
|  | 10b | D | Specify type of model, all model-building procedures (including any predictor selection), and method for internal validation. | 3-4 |
|  | 10c | V | For validation, describe how the predictions were calculated. | 3-4 |
|  | 10d | D;V | Specify all measures used to assess model performance and, if relevant, to compare multiple models. | 3-4 |
|  | 10e | V | Describe any model updating (e.g., recalibration) arising from the validation, if done. | NA |
| Risk groups | 11 | D;V | Provide details on how risk groups were created, if done. | NA |
| Development vs. validation | 12 | V | For validation, identify any differences from the development data in setting, eligibility criteria, outcome, and predictors. | 2-4 |
| **Results** | | | | |
| Participants | 13a | D;V | Describe the flow of participants through the study, including the number of participants with and without the outcome and, if applicable, a summary of the follow-up time. A diagram may be helpful. | 5 |
|  | 13b | D;V | Describe the characteristics of the participants (basic demographics, clinical features, available predictors), including the number of participants with missing data for predictors and outcome. | 5 |
|  | 13c | V | For validation, show a comparison with the development data of the distribution of important variables (demographics, predictors and outcome). | 5 |
| Model development | 14a | D | Specify the number of participants and outcome events in each analysis. | 5 |
|  | 14b | D | If done, report the unadjusted association between each candidate predictor and outcome. | NA |
| Model specification | 15a | D | Present the full prediction model to allow predictions for individuals (i.e., all regression coefficients, and model intercept or baseline survival at a given time point). | 5 |
|  | 15b | D | Explain how to the use the prediction model. | 5 |
| Model performance | 16 | D;V | Report performance measures (with CIs) for the prediction model. | 5 |
| Model-updating | 17 | V | If done, report the results from any model updating (i.e., model specification, model performance). | NA |
| **Discussion** | | | | |
| Limitations | 18 | D;V | Discuss any limitations of the study (such as nonrepresentative sample, few events per predictor, missing data). | 7 |
| Interpretation | 19a | V | For validation, discuss the results with reference to performance in the development data, and any other validation data. | 7 |
|  | 19b | D;V | Give an overall interpretation of the results, considering objectives, limitations, results from similar studies, and other relevant evidence. | 7 |
| Implications | 20 | D;V | Discuss the potential clinical use of the model and implications for future research. | 6-8 |
| **Other information** | | | | |
| Supplementary information | 21 | D;V | Provide information about the availability of supplementary resources, such as study protocol, Web calculator, and data sets. | 8-9 |
| Funding | 22 | D;V | Give the source of funding and the role of the funders for the present study. | 9 |

| **Table S4. The co-expressed genes of *CD161*** | | | | |
| --- | --- | --- | --- | --- |
| Correlated Gene | Cytoband | Spearman's Correlation | p-Value | q-Value |
| CD3E | 11q23.3 | 0.881790531 | 0 | 0 |
| GZMK | 5q11.2 | 0.875505475 | 1.325570114E-315 | 1.3284200897925E-311 |
| CD40LG | Xq26.3 | 0.873906872 | 4.8744787355E-313 | 0.00E+00 |
| JAML | 11q23.3 | 0.871895047 | 7.36701203977477E-310 | 3.69E-306 |
| LY9 | 1q23.3 | 0.871754785 | 0.00E+00 | 4.90E-306 |
| CD3D | 11q23.3 | 0.86454644 | 1.09E-298 | 3.65E-295 |
| SCML4 | 6q21 | 0.856565178 | 2.83E-287 | 8.10E-284 |
| TRAT1 | 3q13.13 | 0.855922561 | 2.19E-286 | 5.48E-283 |
| C16ORF54 | 16p11.2 | 0.854017995 | 8.89E-284 | 1.98E-280 |
| CD48 | 1q23.3 | 0.853703561 | 2.38E-283 | 4.76E-280 |
| SLAMF6 | 1q23.2-q23.3 | 0.85325356 | 9.67E-283 | 1.76E-279 |
| ZNF831 | 20q13.32 | 0.851970933 | 5.15E-281 | 8.60E-278 |
| GZMA | 5q11.2 | 0.850977432 | 1.09E-279 | 1.68E-276 |
| CD247 | 1q24.2 | 0.848878215 | 6.42E-277 | 9.19E-274 |
| SH2D1A | Xq25 | 0.848224063 | 4.59E-276 | 6.13E-273 |
| CD5 | 11q12.2 | 0.845578906 | 1.19E-272 | 1.49E-269 |
| ITK | 5q33.3 | 0.844636154 | 1.90E-271 | 2.24E-268 |
| CD52 | 1p36.11 | 0.84091529 | 8.77E-267 | 9.57E-264 |
| CD2 | 1p13.1 | 0.840903538 | 9.07E-267 | 9.57E-264 |
| SIT1 | 9p13.3 | 0.840446788 | 3.33E-266 | 3.33E-263 |
| CD96 | 3q13.13-q13.2 | 0.839361963 | 7.16E-265 | 6.84E-262 |
| PRKCB | 16p12.2-p12.1 | 0.838997739 | 2.00E-264 | 1.82E-261 |
| BTLA | 3q13.2 | 0.83797607 | 3.50E-263 | 3.05E-260 |
| KLRK1 | 12p13.2 | 0.836744849 | 1.07E-261 | 8.97E-259 |
| UBASH3A | 21q22.3 | 0.835141158 | 8.90E-260 | 7.13E-257 |
| SLAMF1 | 1q23.3 | 0.83471494 | 2.86E-259 | 2.20E-256 |
| PYHIN1 | 1q23.1 | 0.833175325 | 1.87E-257 | 1.39E-254 |
| GPR171 | 3q25.1 | 0.832383249 | 1.59E-256 | 1.14E-253 |
| LCK | 1p35.2 | 0.829391633 | 4.58E-253 | 3.17E-250 |
| TESPA1 | 12q13.2 | 0.828357221 | 6.95E-252 | 4.64E-249 |
| SAMD3 | 6q23.1 | 0.828302784 | 8.01E-252 | 5.18E-249 |
| THEMIS | 6q22.33 | 0.826006355 | 3.13E-249 | 1.96E-246 |
| GIMAP7 | 7q36.1 | 0.825512897 | 1.12E-248 | 6.79E-246 |
| IL2RG | Xq13.1 | 0.825033004 | 3.83E-248 | 2.26E-245 |
| GIMAP5 | 7q36.1 | 0.824788133 | 7.18E-248 | 4.11E-245 |
| CD27 | 12p13.31 | 0.823507261 | 1.88E-246 | 1.05E-243 |
| CLEC10A | 17p13.1 | 0.818519209 | 4.90E-241 | 2.66E-238 |
| ACAP1 | 17p13.1 | 0.817914131 | 2.17E-240 | 1.14E-237 |
| CCR7 | 17q21.2 | 0.817606722 | 4.60E-240 | 2.37E-237 |
| CD8A | 2p11.2 | 0.812004282 | 3.30E-234 | 1.65E-231 |
| TBX21 | 17q21.32 | 0.811244761 | 1.98E-233 | 9.68E-231 |
| SLA2 | 20q11.23 | 0.810568913 | 9.70E-233 | 4.63E-230 |
| S1PR4 | 19p13.3 | 0.810538509 | 1.04E-232 | 4.85E-230 |
| CCL19 | 9p13.3 | 0.809549011 | 1.05E-231 | 4.80E-229 |
| TBC1D10C | 11q13.2 | 0.807018053 | 3.68E-229 | 1.64E-226 |
| CD6 | 11q12.2 | 0.805954229 | 4.21E-228 | 1.83E-225 |
| CXCR3 | Xq13.1 | 0.805297718 | 1.88E-227 | 8.01E-225 |
| CXCR6 | 3p21.31 | 0.804106748 | 2.79E-226 | 1.17E-223 |
| IKZF1 | 7p12.2 | 0.803335091 | 1.59E-225 | 6.50E-223 |
| SIRPG | 20p13 | 0.802979053 | 3.53E-225 | 1.42E-222 |
| P2RY8 | Xp22.33 and Yp11.3 | 0.802535655 | 9.54E-225 | 3.75E-222 |
| CLIC2 | Xq28 | 0.802360668 | 1.41E-224 | 5.44E-222 |
| CD3G | 11q23.3 | 0.797603627 | 5.08E-220 | 1.92E-217 |
| PTPRCAP | 11q13.2 | 0.794554242 | 3.66E-217 | 1.36E-214 |
| ZAP70 | 2q11.2 | 0.793605891 | 2.77E-216 | 1.01E-213 |
| PRF1 | 10q22.1 | 0.79201003 | 8.14E-215 | 2.91E-212 |
| GZMM | 19p13.3 | 0.791894339 | 1.04E-214 | 3.65E-212 |
| CCR2 | 3p21.31 | 0.79155736 | 2.11E-214 | 7.30E-212 |
| ARHGAP15 | 2q22.2-q22.3 | 0.791133217 | 5.15E-214 | 1.75E-211 |
| CD69 | 12p13.31 | 0.790966384 | 7.31E-214 | 2.44E-211 |
| SPOCK2 | 10q22.1 | 0.790708096 | 1.26E-213 | 4.13E-211 |
| PTPN7 | 1q32.1 | 0.790144989 | 4.08E-213 | 1.32E-210 |
| NKG7 | 19q13.41 | 0.788134747 | 2.65E-211 | 8.42E-209 |
| LINC00426 | 13q12.3 | 0.787857303 | 4.69E-211 | 1.47E-208 |
| TNFRSF1B | 1p36.22 | 0.786985961 | 2.82E-210 | 8.69E-208 |
| CRTAM | 11q24.1 | 0.786656311 | 5.54E-210 | 1.68E-207 |
| ARHGAP25 | 2p13.3 | 0.785888939 | 2.66E-209 | 7.95E-207 |
| SPN | 16p11.2 | 0.785409256 | 7.07E-209 | 2.08E-206 |
| GIMAP4 | 7q36.1 | 0.784888134 | 2.04E-208 | 5.92E-206 |
| TMC8 | 17q25.3 | 0.78369144 | 2.29E-207 | 6.56E-205 |
| CCL5 | 17q12 | 0.782725214 | 1.60E-206 | 4.52E-204 |
| CSF2RB | 22q12.3 | 0.782034784 | 6.38E-206 | 1.77E-203 |
| IRAG2 | 12p12.1 | 0.781754513 | 1.12E-205 | 3.06E-203 |
| JAK3 | 19p13.11 | 0.780787818 | 7.64E-205 | 2.07E-202 |
| STAT4 | 2q32.2-q32.3 | 0.78018621 | 2.52E-204 | 6.73E-202 |
| GMFG | 19q13.2 | 0.780034672 | 3.40E-204 | 8.96E-202 |
| MS4A1 | 11q12.2 | 0.779194649 | 1.78E-203 | 4.64E-201 |
| EOMES | 3p24.1 | 0.77857218 | 6.06E-203 | 1.56E-200 |
| MFNG | 22q13.1 | 0.777781018 | 2.85E-202 | 7.24E-200 |
| GFI1 | 1p22.1 | 0.777457471 | 5.37E-202 | 1.35E-199 |
| IL18RAP | 2q12.1 | 0.775306976 | 3.48E-200 | 8.62E-198 |
| CD37 | 19q13.33 | 0.774331356 | 2.28E-199 | 5.56E-197 |
| DCANP1 | 5q31.1 | 0.77296382 | 3.11E-198 | 7.52E-196 |
| IPCEF1 | 6q25.2 | 0.772403563 | 9.05E-198 | 2.16E-195 |
| RASAL3 | 19p13.12 | 0.772055185 | 1.75E-197 | 4.13E-195 |
| SASH3 | Xq26.1 | 0.769462916 | 2.32E-195 | 5.41E-193 |
| CST7 | 20p11.21 | 0.769400492 | 2.61E-195 | 6.01E-193 |
| LGALS2 | 22q13.1 | 0.767860956 | 4.61E-194 | 1.05E-191 |
| MAP4K1 | 19q13.2 | 0.764941777 | 1.00E-191 | 2.26E-189 |
| PRKCQ | 10p15.1 | 0.763328204 | 1.90E-190 | 4.23E-188 |
| GVINP1 | 11p15.4 | 0.762636617 | 6.65E-190 | 1.46E-187 |
| FCRL3 | 1q23.1 | 0.76126204 | 7.93E-189 | 1.73E-186 |
| IL16 | 15q25.1 | 0.760521377 | 3.00E-188 | 6.46E-186 |
| ARHGAP9 | 12q13.3 | 0.76045328 | 3.38E-188 | 7.22E-186 |
| NCR3 | 6p21.33 | 0.759797355 | 1.09E-187 | 2.31E-185 |
| RGL4 | 22q11.23 | 0.758447244 | 1.21E-186 | 2.52E-184 |
| CTSW | 11q13.1 | 0.757994062 | 2.69E-186 | 5.56E-184 |
| PLA2G2D | 1p36.12 | 0.757692711 | 4.58E-186 | 9.38E-184 |
| TNFRSF8 | 1p36.22 | 0.756940307 | 1.73E-185 | 3.50E-183 |
| XCL2 | 1q24.2 | 0.756402295 | 4.45E-185 | 8.91E-183 |
| PPP1R16B | 20q11.23 | 0.754984794 | 5.30E-184 | 1.05E-181 |
| CCDC69 | 5q33.1 | 0.75486981 | 6.48E-184 | 1.27E-181 |
| ITM2A | Xq21.1 | 0.753850689 | 3.81E-183 | 7.41E-181 |
| FLI1 | 11q24.3 | 0.75323839 | 1.10E-182 | 2.12E-180 |
| KLRG1 | 12p13.31 | 0.752043055 | 8.63E-182 | 1.65E-179 |
| CD244 | 1q23.3 | 0.750647966 | 9.41E-181 | 1.78E-178 |
| TRABD2A | 2p11.2 | 0.748284388 | 5.20E-179 | 9.74E-177 |
| HCST | 19q13.12 | 0.748180052 | 6.20E-179 | 1.15E-176 |
| CYTIP | 2q24.1 | 0.748078293 | 7.37E-179 | 1.35E-176 |
| GRAP2 | 22q13.1 | 0.747938515 | 9.32E-179 | 1.70E-176 |
| PLAC8 | 4q21.22 | 0.747328576 | 2.60E-178 | 4.70E-176 |
| GAB3 | Xq28 | 0.747157043 | 3.47E-178 | 6.21E-176 |
| CD79B | 17q23.3 | 0.747013318 | 4.42E-178 | 7.84E-176 |
| INPP5D | 2q37.1 | 0.745504314 | 5.51E-177 | 9.69E-175 |
| CD8B | 2p11.2 | 0.745100357 | 1.08E-176 | 1.88E-174 |
| SELL | 1q24.2 | 0.744778343 | 1.84E-176 | 3.18E-174 |
| IL10RA | 11q23.3 | 0.744165985 | 5.09E-176 | 8.71E-174 |
| GPR18 | 13q32.3 | 0.743520091 | 1.48E-175 | 2.51E-173 |
| PDCD1 | 2q37.3 | 0.743242146 | 2.34E-175 | 3.94E-173 |
| EVI2B | 17q11.2 | 0.742980574 | 3.60E-175 | 6.02E-173 |
| TIGIT | 3q13.31 | 0.742320233 | 1.07E-174 | 1.77E-172 |
| XCL1 | 1q24.2 | 0.741036243 | 8.72E-174 | 1.43E-171 |
| SLC9A9 | 3q24 | 0.740880256 | 1.12E-173 | 1.83E-171 |
| PTPRC | 1q31.3-q32.1 | 0.74042665 | 2.35E-173 | 3.81E-171 |
| HCLS1 | 3q13.33 | 0.739802585 | 6.49E-173 | 1.04E-170 |
| WAS | Xp11.23 | 0.739774834 | 6.79E-173 | 1.08E-170 |
| IRF4 | 6p25.3 | 0.738745445 | 3.59E-172 | 5.67E-170 |
| FCRLA | 1q23.3 | 0.738325952 | 7.07E-172 | 1.11E-169 |
| CCR5 | 3p21.31 | 0.737879841 | 1.45E-171 | 2.25E-169 |
| CD7 | 17q25.3 | 0.737867697 | 1.48E-171 | 2.28E-169 |
| DOK2 | 8p21.3 | 0.736353806 | 1.67E-170 | 2.56E-168 |
| TIFAB | 5q31.1 | 0.736135527 | 2.37E-170 | 3.60E-168 |
| IRF8 | 16q24.1 | 0.736047551 | 2.73E-170 | 4.11E-168 |
| FCRL6 | 1q23.2 | 0.7352515 | 9.68E-170 | 1.45E-167 |
| HLA-DPB1 | 6p21.32 | 0.734203798 | 5.10E-169 | 7.57E-167 |
| BANK1 | 4q24 | 0.734054345 | 6.45E-169 | 9.51E-167 |
| MEI1 | 22q13.2 | 0.733644862 | 1.23E-168 | 1.80E-166 |
| RASGRP2 | 11q13.1 | 0.733389702 | 1.84E-168 | 2.68E-166 |
| C11ORF21 | 11p15.5 | 0.73324406 | 2.32E-168 | 3.34E-166 |
| GPR174 | Xq21.1 | 0.73234425 | 9.53E-168 | 1.36E-165 |
| MAL | 2q11.1 | 0.732288407 | 1.04E-167 | 1.48E-165 |
| IL12RB1 | 19p13.11 | 0.732076783 | 1.45E-167 | 2.05E-165 |
| CD200R1 | 3q13.2 | 0.731944494 | 1.78E-167 | 2.50E-165 |
| CD1C | 1q23.1 | 0.731841144 | 2.10E-167 | 2.92E-165 |
| SLAMF7 | 1q23.3 | 0.731641626 | 2.86E-167 | 3.96E-165 |
| CXCR5 | 11q23.3 | 0.731377556 | 4.32E-167 | 5.94E-165 |
| LAT | 16q13 | 0.730918306 | 8.85E-167 | 1.21E-164 |
| SEPTIN1 | 16p11.2 | 0.730334104 | 2.20E-166 | 2.98E-164 |
| TNFRSF13B | 17p11.2 | 0.729856191 | 4.61E-166 | 6.21E-164 |
| TRAF3IP3 | 1q32.2 | 0.729688837 | 5.98E-166 | 7.99E-164 |
| RGS18 | 1q31.2 | 0.72941099 | 9.20E-166 | 1.22E-163 |
| AKNA | 9q32 | 0.729407349 | 9.25E-166 | 1.22E-163 |
| CD1D | 1q23.1 | 0.729074675 | 1.55E-165 | 2.03E-163 |
| CD79A | 19q13.2 | 0.728704001 | 2.74E-165 | 3.57E-163 |
| TOX | 8q12.1 | 0.728084218 | 7.12E-165 | 9.21E-163 |
| BLK | 8p23.1 | 0.727839791 | 1.04E-164 | 1.33E-162 |
| GPR183 | 13q32.3 | 0.7276514 | 1.39E-164 | 1.77E-162 |
| PSTPIP1 | 15q24.3 | 0.727278132 | 2.46E-164 | 3.12E-162 |
| GIMAP8 | 7q36.1 | 0.726398695 | 9.43E-164 | 1.19E-161 |
| CD53 | 1p13.3 | 0.72338569 | 9.11E-162 | 1.14E-159 |
| PIK3CD | 1p36.22 | 0.7232425 | 1.13E-161 | 1.41E-159 |
| GZMH | 14q12 | 0.722417558 | 3.90E-161 | 4.83E-159 |
| GIMAP6 | 7q36.1 | 0.722196996 | 5.43E-161 | 6.68E-159 |
| SNX20 | 16q12.1 | 0.72207395 | 6.53E-161 | 7.99E-159 |
| NUGGC | 8p21.1 | 0.721437872 | 1.69E-160 | 2.06E-158 |
| IL12B | 5q33.3 | 0.720949416 | 3.51E-160 | 4.23E-158 |
| EBI3 | 19p13.3 | 0.720317268 | 8.99E-160 | 1.08E-157 |
| WDFY4 | 10q11.23 | 0.718471282 | 1.38E-158 | 1.65E-156 |
| GIMAP1 | 7q36.1 | 0.718289368 | 1.81E-158 | 2.14E-156 |
| HSD11B1 | 1q32.2 | 0.718283554 | 1.82E-158 | 2.15E-156 |
| KLHL6 | 3q27.1 | 0.718014507 | 2.71E-158 | 3.18E-156 |
| ASB2 | 14q32.12 | 0.717459214 | 6.13E-158 | 7.14E-156 |
| RCSD1 | 1q24.2 | 0.716873401 | 1.45E-157 | 1.68E-155 |
| FAM78A | 9q34.13 | 0.715747293 | 7.49E-157 | 8.63E-155 |
| SPIB | 19q13.33 | 0.714808725 | 2.93E-156 | 3.36E-154 |
| BCL11B | 14q32.2 | 0.714580383 | 4.09E-156 | 4.65E-154 |
| TCL1A | 14q32.13 | 0.714308711 | 6.06E-156 | 6.86E-154 |
| BIN2 | 12q13.13 | 0.714220525 | 6.88E-156 | 7.75E-154 |
| IL7R | 5p13.2 | 0.713762845 | 1.33E-155 | 1.49E-153 |
| VSIR | 10q22.1 | 0.713571519 | 1.76E-155 | 1.96E-153 |
| LTB | 6p21.33 | 0.713520605 | 1.89E-155 | 2.10E-153 |
| NLRC3 | 16p13.3 | 0.713223524 | 2.91E-155 | 3.20E-153 |
| AOAH | 7p14.2 | 0.713085815 | 3.54E-155 | 3.88E-153 |
| FCER2 | 19p13.2 | 0.713071189 | 3.62E-155 | 3.94E-153 |
| PCED1B-AS1 | 12q13.11 | 0.712462139 | 8.69E-155 | 9.42E-153 |
| ABCD2 | 12q12 | 0.712336681 | 1.04E-154 | 1.12E-152 |
| CD28 | 2q33.2 | 0.711902131 | 1.94E-154 | 2.08E-152 |
| FASLG | 1q24.3 | 0.711451461 | 3.70E-154 | 3.95E-152 |
| CORO1A | 16p11.2 | 0.710560881 | 1.32E-153 | 1.40E-151 |
| IL2RB | 22q12.3 | 0.710256889 | 2.04E-153 | 2.15E-151 |
| TMIGD2 | 19p13.3 | 0.709297918 | 7.95E-153 | 8.34E-151 |
| HLA-DRA | 6p21.32 | 0.706674399 | 3.21E-151 | 3.35E-149 |
| GGTA1 | 9q33.2 | 0.7045769 | 5.99E-150 | 6.22E-148 |
| CNR2 | 1p36.11 | 0.703818934 | 1.71E-149 | 1.77E-147 |
| FCRL1 | 1q23.1 | 0.703050841 | 4.96E-149 | 5.10E-147 |
| PTGDS | 9q34.3 | 0.70250273 | 1.06E-148 | 1.08E-146 |
| DNASE1L3 | 3p14.3 | 0.702262412 | 1.47E-148 | 1.50E-146 |
| JCHAIN | 4q13.3 | 0.701847242 | 2.60E-148 | 2.63E-146 |
| KCNA3 | 1p13.3 | 0.700469601 | 1.72E-147 | 1.73E-145 |
| FCN1 | 9q34.3 | 0.700263821 | 2.27E-147 | 2.28E-145 |
| LAX1 | 1q32.1 | 0.699499175 | 6.44E-147 | 6.42E-145 |
| CD1B | 1q23.1 | 0.699415528 | 7.22E-147 | 7.16E-145 |
| POU2AF1 | 11q23.1 | 0.69910999 | 1.09E-146 | 1.08E-144 |
| ZNF80 | 3q13.31 | 0.698468139 | 2.61E-146 | 2.56E-144 |
| CD19 | 16p11.2 | 0.698412761 | 2.81E-146 | 2.75E-144 |
| CD1E | 1q23.1 | 0.698045356 | 4.63E-146 | 4.50E-144 |
| SELPLG | 12q24.11 | 0.697912472 | 5.54E-146 | 5.36E-144 |
| FGL2 | 7q11.23 | 0.697299004 | 1.27E-145 | 1.22E-143 |
| HLA-DPA1 | 6p21.32 | 0.697029821 | 1.82E-145 | 1.75E-143 |
| P2RY14 | 3q25.1 | 0.696900236 | 2.17E-145 | 2.07E-143 |
| SELP | 1q24.2 | 0.695512273 | 1.40E-144 | 1.33E-142 |
| LCP2 | 5q35.1 | 0.695391641 | 1.64E-144 | 1.55E-142 |
| SP140 | 2q37.1 | 0.693989657 | 1.06E-143 | 1.00E-141 |
| CXCL9 | 4q21.1 | 0.693696101 | 1.57E-143 | 1.47E-141 |
| CARD11 | 7p22.2 | 0.693263084 | 2.79E-143 | 2.60E-141 |
| HLA-DOA | 6p21.32 | 0.692760985 | 5.42E-143 | 5.03E-141 |
| ARHGAP30 | 1q23.3 | 0.689984485 | 2.09E-141 | 1.93E-139 |
| CD4 | 12p13.31 | 0.689753088 | 2.83E-141 | 2.60E-139 |
| PTGER4 | 5p13.1 | 0.689406608 | 4.45E-141 | 4.07E-139 |
| WIPF1 | 2q31.1 | 0.687971602 | 2.88E-140 | 2.62E-138 |
| SLA | 8q24.22 | 0.687240963 | 7.41E-140 | 6.72E-138 |
| FGR | 1p35.3 | 0.687232278 | 7.50E-140 | 6.77E-138 |
| PLEK | 2p14 | 0.686420165 | 2.14E-139 | 1.92E-137 |
| FCRL2 | 1q23.1 | 0.685880838 | 4.28E-139 | 3.83E-137 |
| SLFN12L | 17q12 | 0.685064492 | 1.22E-138 | 1.09E-136 |
| CLEC9A | 12p13.2 | 0.68476258 | 1.80E-138 | 1.60E-136 |
| CD226 | 18q22.2 | 0.684321698 | 3.16E-138 | 2.79E-136 |
| LTA | 6p21.33 | 0.683791185 | 6.23E-138 | 5.47E-136 |
| PPP2R2B | 5q32 | 0.683460097 | 9.49E-138 | 8.31E-136 |
| WNT1 | 12q13.12 | 0.682229776 | 4.53E-137 | 3.95E-135 |
| MPEG1 | 11q12.1 | 0.681751779 | 8.30E-137 | 7.20E-135 |
| TLR10 | 4p14 | 0.681656452 | 9.37E-137 | 8.09E-135 |
| RAC2 | 22q13.1 | 0.68148294 | 1.17E-136 | 1.00E-134 |
| NCKAP1L | 12q13.13-q13.2 | 0.681023799 | 2.08E-136 | 1.78E-134 |
| GZMB | 14q12 | 0.680582984 | 3.63E-136 | 3.10E-134 |
| P2RY10 | Xq21.1 | 0.68044382 | 4.33E-136 | 3.67E-134 |
| CIITA | 16p13.13 | 0.679530622 | 1.36E-135 | 1.15E-133 |
| FMNL1 | 17q21.31 | 0.679116917 | 2.29E-135 | 1.93E-133 |
| RIPOR2 | 6p22.3 | 0.678949949 | 2.82E-135 | 2.36E-133 |
| FYB1 | 5p13.1 | 0.67891377 | 2.95E-135 | 2.46E-133 |
| CD40 | 20q13.12 | 0.678726639 | 3.73E-135 | 3.10E-133 |
| IL18R1 | 2q12.1 | 0.678338448 | 6.05E-135 | 5.01E-133 |
| MS4A6A | 11q12.2 | 0.677491298 | 1.74E-134 | 1.43E-132 |
| TAGAP | 6q25.3 | 0.675753422 | 1.50E-133 | 1.23E-131 |
| HVCN1 | 12q24.11 | 0.675668572 | 1.66E-133 | 1.36E-131 |
| CCL17 | 16q21 | 0.67526287 | 2.75E-133 | 2.24E-131 |
| BTK | Xq22.1 | 0.675243391 | 2.81E-133 | 2.28E-131 |
| ABCB1 | 7q21.12 | 0.674858677 | 4.52E-133 | 3.65E-131 |
| HLA-DMB | 6p21.32 | 0.674449183 | 7.47E-133 | 6.02E-131 |
| ZNF683 | 1p36.11 | 0.674301736 | 8.96E-133 | 7.18E-131 |
| APOBEC3G | 22q13.1 | 0.674220847 | 9.89E-133 | 7.90E-131 |
| DOCK2 | 5q35.1 | 0.673479694 | 2.45E-132 | 1.95E-130 |
| AIF1 | 6p21.33 | 0.672715954 | 6.24E-132 | 4.95E-130 |
| APBB1IP | 10p12.1 | 0.672426012 | 8.89E-132 | 7.02E-130 |
| TNFRSF17 | 16p13.13 | 0.672304898 | 1.03E-131 | 8.10E-130 |
| C1ORF162 | 1p13.2 | 0.672038591 | 1.43E-131 | 1.12E-129 |
| EVI2A | 17q11.2 | 0.671115893 | 4.37E-131 | 3.41E-129 |
| LPXN | 11q12.1 | 0.670852735 | 6.02E-131 | 4.67E-129 |
| FGD2 | 6p21.2 | 0.670596588 | 8.20E-131 | 6.35E-129 |
| HLA-DMA | 6p21.32 | 0.670104025 | 1.49E-130 | 1.15E-128 |
| LST1 | 6p21.33 | 0.66994122 | 1.81E-130 | 1.39E-128 |
| KLRC1 | 12p13 | 0.669628355 | 2.64E-130 | 2.02E-128 |
| TMEM273 | 10q11.23 | 0.66962677 | 2.65E-130 | 2.02E-128 |
| ITPRIPL1 | 2q11.2 | 0.669278687 | 4.03E-130 | 3.06E-128 |
| KLRD1 | 12p13.2 | 0.66850293 | 1.02E-129 | 7.74E-128 |
| PECAM1 | 17q23.3 | 0.66805606 | 1.75E-129 | 1.32E-127 |
| BHLHE22 | 8q12.3 | 0.667984461 | 1.91E-129 | 1.43E-127 |
| ACKR1 | 1q23.2 | 0.667344916 | 4.10E-129 | 3.06E-127 |
| CFP | Xp11.23 | 0.667186021 | 4.95E-129 | 3.69E-127 |
| PRDM8 | 4q21.21 | 0.666673527 | 9.13E-129 | 6.78E-127 |
| TREML2 | 6p21.1 | 0.66629568 | 1.43E-128 | 1.06E-126 |
| TRAF1 | 9q33.2 | 0.666270106 | 1.48E-128 | 1.09E-126 |
| CD74 | 5q33.1 | 0.665495737 | 3.70E-128 | 2.72E-126 |
| ADGRG5 | 16q21 | 0.664908496 | 7.43E-128 | 5.43E-126 |
| MYO1F | 19p13.2 | 0.664761831 | 8.84E-128 | 6.44E-126 |
| NAPSB | 19q13.33 | 0.664726224 | 9.22E-128 | 6.69E-126 |
| GPSM3 | 6p21.32 | 0.66383584 | 2.64E-127 | 1.91E-125 |
| LRRC2 | 3p21.31 | 0.663645178 | 3.30E-127 | 2.38E-125 |
| FAM30A | 14q32.33 | 0.661940099 | 2.44E-126 | 1.76E-124 |
| CCR4 | 3p22.3 | 0.661857275 | 2.69E-126 | 1.93E-124 |
| FCRL5 | 1q23.1 | 0.66181261 | 2.84E-126 | 2.02E-124 |
| PARP15 | 3q21.1 | 0.66136443 | 4.79E-126 | 3.41E-124 |
| IL33 | 9p24.1 | 0.661180694 | 5.94E-126 | 4.21E-124 |
| POU2F2 | 19q13.2 | 0.661038713 | 7.01E-126 | 4.95E-124 |
| DTHD1 | 4p14 | 0.66049544 | 1.32E-125 | 9.28E-124 |
| ICOS | 2q33.2 | 0.660223527 | 1.81E-125 | 1.27E-123 |
| RAB33A | Xq26.1 | 0.660028756 | 2.27E-125 | 1.59E-123 |
| NCF4 | 22q12.3 | 0.65993937 | 2.52E-125 | 1.75E-123 |
| PARVG | 22q13.31 | 0.6598569 | 2.77E-125 | 1.92E-123 |
| DPEP2 | 16q22.1 | 0.658779414 | 9.66E-125 | 6.68E-123 |
| ABI3BP | 3q12.2 | 0.658071296 | 2.19E-124 | 1.51E-122 |
| RNASE6 | 14q11.2 | 0.657839036 | 2.86E-124 | 1.96E-122 |
| SAMSN1 | 21q11.2 | 0.657741305 | 3.20E-124 | 2.19E-122 |
| FUT7 | 9q34.3 | 0.65709719 | 6.71E-124 | 4.58E-122 |
| CELF2 | 10p14 | 0.656992458 | 7.57E-124 | 5.14E-122 |
| CR1 | 1q32.2 | 0.656420753 | 1.46E-123 | 9.87E-122 |
| CLEC1A | 12p13.2 | 0.656146895 | 2.00E-123 | 1.35E-121 |
| XPNPEP2 | Xq26.1 | 0.655474236 | 4.30E-123 | 2.90E-121 |
| LY96 | 8q21.11 | 0.655388332 | 4.75E-123 | 3.18E-121 |
| SLC12A3 | 16q13 | 0.654359358 | 1.53E-122 | 1.02E-120 |
| GPR55 | 2q37.1 | 0.65391559 | 2.54E-122 | 1.69E-120 |
| IL18BP | 11q13.4 | 0.651509037 | 3.85E-121 | 2.55E-119 |
| TNFAIP8L2 | 1q21.3 | 0.651019207 | 6.67E-121 | 4.41E-119 |
| ADAM6 | 14q32.33 | 0.650698401 | 9.56E-121 | 6.30E-119 |
| PNOC | 8p21.1 | 0.650590514 | 1.08E-120 | 7.09E-119 |
| GNGT2 | 17q21.32 | 0.649788487 | 2.65E-120 | 1.73E-118 |
| HLA-DOB | 6p21.32 | 0.649239819 | 4.88E-120 | 3.19E-118 |
| HLA-E | 6p22.1 | 0.648700976 | 8.89E-120 | 5.79E-118 |
| FERMT3 | 11q13.1 | 0.648624408 | 9.68E-120 | 6.28E-118 |
| LYZ | 12q15 | 0.648420385 | 1.22E-119 | 7.86E-118 |
| SNAI3 | 16q24.2 | 0.647331973 | 4.06E-119 | 2.62E-117 |
| SCIMP | 17p13.2 | 0.646454918 | 1.07E-118 | 6.88E-117 |
| TNFAIP3 | 6q23.3 | 0.646046791 | 1.68E-118 | 1.08E-116 |
| CLECL1 | 12p13.31 | 0.64588424 | 2.01E-118 | 1.28E-116 |
| PDE6G | 17q25.3 | 0.645428016 | 3.32E-118 | 2.11E-116 |
| PTGDR | 14q22.1 | 0.645266221 | 3.96E-118 | 2.51E-116 |
| CTLA4 | 2q33.2 | 0.644923724 | 5.77E-118 | 3.65E-116 |
| TXK | 4p12 | 0.644913617 | 5.84E-118 | 3.68E-116 |
| CD38 | 4p15.32 | 0.644323787 | 1.11E-117 | 7.00E-116 |
| APOBEC3H | 22q13.1 | 0.644214122 | 1.26E-117 | 7.86E-116 |
| GNG2 | 14q22.1 | 0.643850072 | 1.87E-117 | 1.17E-115 |
| IDO2 | 8p11.21 | 0.64231613 | 9.94E-117 | 6.19E-115 |
| C3 | 19p13.3 | 0.641941157 | 1.49E-116 | 9.27E-115 |
| CPVL | 7p14.3 | 0.641459866 | 2.52E-116 | 1.56E-114 |
| CCL21 | 9p13.3 | 0.639422818 | 2.26E-115 | 1.40E-113 |
| PVRIG | 7q22.1 | 0.639223757 | 2.80E-115 | 1.72E-113 |
| S1PR1 | 1p21.2 | 0.638691392 | 4.96E-115 | 3.04E-113 |
| LILRA4 | 19q13.42 | 0.638152006 | 8.84E-115 | 5.40E-113 |
| MEOX1 | 17q21.31 | 0.638082567 | 9.53E-115 | 5.80E-113 |
| CLEC4A | 12p13.31 | 0.637978777 | 1.06E-114 | 6.46E-113 |
| ICAM2 | 17q23.3 | 0.637059926 | 2.84E-114 | 1.72E-112 |
| P2RX1 | 17p13.2 | 0.636573266 | 4.76E-114 | 2.88E-112 |
| GNLY | 2p11.2 | 0.63653027 | 4.99E-114 | 3.00E-112 |
| ALDH1A1 | 9q21.13 | 0.635569334 | 1.38E-113 | 8.29E-112 |
| CLEC12A | 12p13.31 | 0.635522886 | 1.45E-113 | 8.69E-112 |
| ABI3 | 17q21.32 | 0.634435599 | 4.58E-113 | 2.73E-111 |
| TSPAN32 | 11p15.5 | 0.634352016 | 5.00E-113 | 2.98E-111 |
| TTC24 | 1q22 | 0.634236571 | 5.65E-113 | 3.35E-111 |
| STAP1 | 4q13.2 | 0.634134747 | 6.29E-113 | 3.72E-111 |
| TLR8 | Xp22.2 | 0.634129155 | 6.33E-113 | 3.73E-111 |
| CCL23 | 17q12 | 0.63405354 | 6.85E-113 | 4.03E-111 |
| IL3RA | Xp22.3 and Yp13.3 | 0.633738087 | 9.55E-113 | 5.60E-111 |
| AGAP2 | 12q14.1 | 0.633704608 | 9.90E-113 | 5.78E-111 |
| ENPP2 | 8q24.12 | 0.632912485 | 2.27E-112 | 1.33E-110 |
| FYN | 6q21 | 0.632892864 | 2.32E-112 | 1.35E-110 |
| PIK3R5 | 17p13.1 | 0.632313936 | 4.26E-112 | 2.47E-110 |
| TNFSF14 | 19p13.3 | 0.631579815 | 9.17E-112 | 5.30E-110 |
| BEND4 | 4p13 | 0.63092106 | 1.82E-111 | 1.05E-109 |
| LSP1 | 11p15.5 | 0.630293758 | 3.50E-111 | 2.01E-109 |
| BIRC3 | 11q22.2 | 0.629781742 | 5.96E-111 | 3.41E-109 |
| PTPN22 | 1p13.2 | 0.629677231 | 6.64E-111 | 3.79E-109 |
| PAX5 | 9p13.2 | 0.629405403 | 8.80E-111 | 5.01E-109 |
| ETS1 | 11q24.3 | 0.629207092 | 1.08E-110 | 6.13E-109 |
| NIBAN3 | 19p13.11 | 0.62907813 | 1.23E-110 | 6.99E-109 |
| GYPC | 2q14.3 | 0.628607818 | 2.01E-110 | 1.13E-108 |
| CASP10 | 2q33.1 | 0.627754575 | 4.84E-110 | 2.72E-108 |
| TNFAIP8 | 5q23.1 | 0.627364775 | 7.22E-110 | 4.06E-108 |
| ARRDC5 | 19p13.3 | 0.627360174 | 7.26E-110 | 4.06E-108 |
| CYTH4 | 22q13.1 | 0.627059346 | 9.89E-110 | 5.52E-108 |
| TOX2 | 20q13.12 | 0.625203637 | 6.60E-109 | 3.68E-107 |
| ADA2 | 22q11.1 | 0.624822385 | 9.74E-109 | 5.41E-107 |
| CDHR1 | 10q23.1 | 0.624336538 | 1.60E-108 | 8.84E-107 |
| RUNX3 | 1p36.11 | 0.624280159 | 1.69E-108 | 9.33E-107 |
| WNT10A | 2q35 | 0.623828584 | 2.67E-108 | 1.47E-106 |
| CCDC141 | 2q31.2 | 0.623801141 | 2.75E-108 | 1.51E-106 |
| CD300LF | 17q25.1 | 0.62354513 | 3.56E-108 | 1.95E-106 |
| BMS1P20 | 22q11.22 | 0.623467488 | 3.86E-108 | 2.11E-106 |
| FREM1 | 9p22.3 | 0.622377457 | 1.16E-107 | 6.32E-106 |
| RTP5 | 2q37.3 | 0.620865272 | 5.32E-107 | 2.89E-105 |
| CLEC17A | 19p13.12 | 0.620718299 | 6.17E-107 | 3.34E-105 |
| NLRP1 | 17p13 | 0.620161338 | 1.08E-106 | 5.82E-105 |
| PLCB2 | 15q15.1 | 0.619806164 | 1.54E-106 | 8.28E-105 |
| P2RY13 | 3q25.1 | 0.619624739 | 1.84E-106 | 9.90E-105 |
| TRPV2 | 17p11.2 | 0.619228132 | 2.74E-106 | 1.47E-104 |
| VNN2 | 6q23.2 | 0.618720709 | 4.54E-106 | 2.43E-104 |
| CTSS | 1q21.3 | 0.618598718 | 5.13E-106 | 2.73E-104 |
| ELMO1 | 7p14.2-p14.1 | 0.618246432 | 7.28E-106 | 3.87E-104 |
| CADM3 | 1q23.2 | 0.617820784 | 1.11E-105 | 5.89E-104 |
| IL21R | 16p12.1 | 0.617400074 | 1.69E-105 | 8.92E-104 |
| MIR155HG | 21q21.3 | 0.616982644 | 2.55E-105 | 1.35E-103 |
| PLEKHO2 | 15q22.31 | 0.615946564 | 7.10E-105 | 3.74E-103 |
| ASGR2 | 17p13.1 | 0.615866502 | 7.69E-105 | 4.03E-103 |
| CPNE5 | 6p21.2 | 0.615769881 | 8.45E-105 | 4.42E-103 |
| SH2D3C | 9q34.11 | 0.614833938 | 2.12E-104 | 1.11E-102 |
| CCL4 | 17q12 | 0.614479392 | 3.01E-104 | 1.57E-102 |
| SERPINB9 | 6p25.2 | 0.614350006 | 3.41E-104 | 1.77E-102 |
| C1ORF54 | 1q21.2 | 0.613574602 | 7.29E-104 | 3.78E-102 |
| NRROS | 3q29 | 0.613068443 | 1.20E-103 | 6.18E-102 |
| CAMK4 | 5q22.1 | 0.61199915 | 3.39E-103 | 1.75E-101 |
| CARD16 | 11q22.3 | 0.611460938 | 5.72E-103 | 2.94E-101 |
| ITGAL | 16p11.2 | 0.610644445 | 1.26E-102 | 6.46E-101 |
| GBP4 | 1p22.2 | 0.61056055 | 1.37E-102 | 6.99E-101 |
| IFNG | 12q15 | 0.609321485 | 4.52E-102 | 2.31E-100 |
| SLC25A53 | Xq22.2 | 0.608550785 | 9.49E-102 | 4.83E-100 |
| CCL13 | 17q12 | 0.607044872 | 4.01E-101 | 2.04E-99 |
| PAPLN | 14q24.2 | 0.606893075 | 4.64E-101 | 2.35E-99 |
| MRC1 | 10p12.33 | 0.606716664 | 5.49E-101 | 2.77E-99 |
| PDCD1LG2 | 9p24.1 | 0.605969241 | 1.12E-100 | 5.64E-99 |
| STX11 | 6q24.2 | 0.60559514 | 1.60E-100 | 8.03E-99 |
| CALHM6 | 6q22.1 | 0.604305954 | 5.42E-100 | 2.72E-98 |
| GBP5 | 1p22.2 | 0.603313035 | 1.39E-99 | 6.92E-98 |
| VAMP5 | 2p11.2 | 0.602697011 | 2.47E-99 | 1.23E-97 |
| HLA-DQA1 | 6p21.32 | 0.602071666 | 4.45E-99 | 2.21E-97 |
| PLCL2 | 3p24.3 | 0.601522613 | 7.45E-99 | 3.70E-97 |
| CMKLR1 | 12q23.3 | 0.601219442 | 9.90E-99 | 4.90E-97 |
| HLA-DRB1 | 6p21.32 | 0.600855583 | 1.39E-98 | 6.87E-97 |
| CLEC4C | 12p13.31 | 0.600773608 | 1.50E-98 | 7.39E-97 |
| MS4A4A | 11q12.2 | 0.600419551 | 2.09E-98 | 1.03E-96 |
| GRAP | 17p11.2 | 0.600330915 | 2.27E-98 | 1.11E-96 |
| SCARA5 | 8p21.1 | 0.600148005 | 2.69E-98 | 1.32E-96 |
| CLIC5 | 6p21.1 | 0.599580743 | 4.56E-98 | 2.23E-96 |
| LINC01140 | 1p22.3 | 0.599128775 | 6.95E-98 | 3.38E-96 |
| ACVRL1 | 12q13.13 | 0.598329541 | 1.46E-97 | 7.08E-96 |
| GIMAP2 | 7q36.1 | 0.597497617 | 3.15E-97 | 1.52E-95 |
| C1QA | 1p36.12 | 0.597393972 | 3.46E-97 | 1.67E-95 |
| NLRP3 | 1q44 | 0.597146805 | 4.35E-97 | 2.10E-95 |
| AMPD1 | 1p13.2 | 0.597033125 | 4.83E-97 | 2.32E-95 |
| LAMP3 | 3q27.1 | 0.596349961 | 9.06E-97 | 4.35E-95 |
| CCR6 | 6q27 | 0.59614504 | 1.09E-96 | 5.23E-95 |
| FOLR2 | 11q13.4 | 0.595991289 | 1.26E-96 | 6.01E-95 |
| LIMD2 | 17q23.3 | 0.595797537 | 1.51E-96 | 7.17E-95 |
| LAIR1 | 19q13.42 | 0.595780427 | 1.53E-96 | 7.26E-95 |
| ICAM4 | 19p13.2 | 0.595114617 | 2.82E-96 | 1.33E-94 |
| ITGB7 | 12q13.13 | 0.593406241 | 1.34E-95 | 6.33E-94 |
| VAV1 | 19p13.3 | 0.592984481 | 1.97E-95 | 9.27E-94 |
| BMP6 | 6p24.3 | 0.592860166 | 2.20E-95 | 1.04E-93 |
| FOXP3 | Xp11.23 | 0.592587298 | 2.82E-95 | 1.32E-93 |
| CXORF65 | Xq13.1 | 0.591933752 | 5.10E-95 | 2.39E-93 |
| XCR1 | 3p21.31 | 0.591902676 | 5.25E-95 | 2.45E-93 |
| IL15RA | 10p15.1 | 0.591727448 | 6.15E-95 | 2.87E-93 |
| PLCG2 | 16q24.1 | 0.591686538 | 6.38E-95 | 2.97E-93 |
| NCF1 | 7q11.23 | 0.591621791 | 6.77E-95 | 3.14E-93 |
| CH25H | 10q23.31 | 0.591478174 | 7.70E-95 | 3.57E-93 |
| IDO1 | 8p11.21 | 0.591241639 | 9.54E-95 | 4.41E-93 |
| SPNS3 | 17p13.2 | 0.590160987 | 2.53E-94 | 1.16E-92 |
| SERPING1 | 11q12.1 | 0.589544031 | 4.40E-94 | 2.02E-92 |
| ABCA6 | 17q24.2-q24.3 | 0.589407327 | 4.98E-94 | 2.28E-92 |
| CCL22 | 16q21 | 0.588899063 | 7.85E-94 | 3.59E-92 |
| ZBED2 | 3q13.13 | 0.588220534 | 1.44E-93 | 6.58E-92 |
| ICAM3 | 19p13.2 | 0.58759313 | 2.52E-93 | 1.15E-91 |
| MYO1G | 7p13 | 0.587483457 | 2.78E-93 | 1.26E-91 |
| GYPE | 4q31.21 | 0.586575014 | 6.25E-93 | 2.83E-91 |
| LCN10 | 9q34.3 | 0.586379261 | 7.43E-93 | 3.36E-91 |
| IL2 | 4q27 | 0.586112306 | 9.42E-93 | 4.25E-91 |
| LAPTM5 | 1p35.2 | 0.585855327 | 1.18E-92 | 5.33E-91 |
| RASSF5 | 1q32.1 | 0.585725982 | 1.33E-92 | 5.96E-91 |
| CCND2 | 12p13.32 | 0.58548978 | 1.64E-92 | 7.34E-91 |
| CLEC2B | 12p13.31 | 0.585353873 | 1.85E-92 | 8.25E-91 |
| CSF2RA | Xp22.32 and Yp11.3 | 0.584808628 | 2.99E-92 | 1.33E-90 |
| C7 | 5p13.1 | 0.584411912 | 4.24E-92 | 1.89E-90 |
| TVP23A | 16p13.13 | 0.583801169 | 7.26E-92 | 3.23E-90 |
| APOL3 | 22q12.3 | 0.583095353 | 1.35E-91 | 5.99E-90 |
| TNIP3 | 4q27 | 0.582709 | 1.90E-91 | 8.39E-90 |
| NCF1C | 7q11.23 | 0.58196396 | 3.64E-91 | 1.61E-89 |
| TASL | Xp21.2 | 0.581515175 | 5.39E-91 | 2.37E-89 |
| APOBEC3C | 22q13.1 | 0.58120591 | 7.06E-91 | 3.10E-89 |
| UBD | 6p22.1 | 0.580290094 | 1.57E-90 | 6.88E-89 |
| CR2 | 1q32.2 | 0.58012196 | 1.81E-90 | 7.94E-89 |
| CLNK | 4p16.1 | 0.580085252 | 1.87E-90 | 8.18E-89 |
| KCTD12 | 13q22.3 | 0.579777369 | 2.45E-90 | 1.07E-88 |
| RASSF2 | 20p13 | 0.578968298 | 4.94E-90 | 2.15E-88 |
| IL2RA | 10p15.1 | 0.578110501 | 1.04E-89 | 4.50E-88 |
| FLT3LG | 19q13.33 | 0.578045272 | 1.10E-89 | 4.75E-88 |
| HLA-DQB1 | 6p21.32 | 0.577778959 | 1.38E-89 | 5.96E-88 |
| CEACAM21 | 19q13.2 | 0.577383307 | 1.94E-89 | 8.36E-88 |
| CXCL13 | 4q21.1 | 0.576441977 | 4.36E-89 | 1.87E-87 |
| SLAMF8 | 1q23.2 | 0.575307149 | 1.15E-88 | 4.94E-87 |
| CASS4 | 20q13.31 | 0.575282737 | 1.18E-88 | 5.03E-87 |
| INSL3 | 19p13.11 | 0.575149857 | 1.32E-88 | 5.63E-87 |
| IFI16 | 1q23.1 | 0.574669178 | 1.98E-88 | 8.46E-87 |
| AIM2 | 1q23.1-q23.2 | 0.574460637 | 2.37E-88 | 1.01E-86 |
| TCF7 | 5q31.1 | 0.574172824 | 3.03E-88 | 1.29E-86 |
| KIF21B | 1q32.1 | 0.573952462 | 3.65E-88 | 1.55E-86 |
| LCP1 | 13q14.13 | 0.573623131 | 4.84E-88 | 2.04E-86 |
| TMEM156 | 4p14 | 0.573430673 | 5.69E-88 | 2.40E-86 |
| CLEC4E | 12p13.31 | 0.573235063 | 6.72E-88 | 2.83E-86 |
| C1S | 12p13.31 | 0.573084361 | 7.64E-88 | 3.21E-86 |
| PRDM1 | 6q21 | 0.572984337 | 8.32E-88 | 3.49E-86 |
| ACSL5 | 10q25.2 | 0.572915952 | 8.81E-88 | 3.69E-86 |
| SLC24A4 | 14q32.12 | 0.57276533 | 1.00E-87 | 4.18E-86 |
| TACR1 | 2p12 | 0.572702844 | 1.06E-87 | 4.40E-86 |
| IGSF6 | 16p12.2 | 0.572583612 | 1.17E-87 | 4.86E-86 |
| ITGB2 | 21q22.3 | 0.571964277 | 1.97E-87 | 8.18E-86 |
| KLRF1 | 12p13.31 | 0.571907636 | 2.07E-87 | 8.57E-86 |
| SOCS1 | 16p13.13 | 0.570992501 | 4.48E-87 | 1.85E-85 |
| APOBEC3D | 22q13.1 | 0.570744354 | 5.52E-87 | 2.28E-85 |
| RSPO3 | 6q22.33 | 0.570471241 | 6.94E-87 | 2.86E-85 |
| GPR65 | 14q31.3 | 0.569269422 | 1.90E-86 | 7.81E-85 |
| HSF5 | 17q22 | 0.567734515 | 6.85E-86 | 2.81E-84 |
| LILRB2 | 19q13.4 | 0.567432854 | 8.81E-86 | 3.60E-84 |
| CD72 | 9p13.3 | 0.567247767 | 1.03E-85 | 4.20E-84 |
| IL32 | 16p13.3 | 0.567114717 | 1.15E-85 | 4.68E-84 |
| SLC22A3 | 6q25.3 | 0.566667936 | 1.66E-85 | 6.76E-84 |
| COL4A4 | 2q36.3 | 0.565669512 | 3.80E-85 | 1.54E-83 |
| MNDA | 1q23.1 | 0.565571428 | 4.13E-85 | 1.67E-83 |
| MEOX2 | 7p21.2 | 0.56517583 | 5.72E-85 | 2.31E-83 |
| TNFRSF9 | 1p36.23 | 0.565014294 | 6.54E-85 | 2.64E-83 |
| MCTP1 | 5q15 | 0.564810273 | 7.73E-85 | 3.11E-83 |
| VPREB3 | 22q11.23 | 0.564525384 | 9.78E-85 | 3.93E-83 |
| MILR1 | 17q23.3 | 0.563024105 | 3.36E-84 | 1.35E-82 |
| GP1BA | 17p13.2 | 0.562955058 | 3.55E-84 | 1.42E-82 |
| SH2D1B | 1q23.3 | 0.562925842 | 3.64E-84 | 1.45E-82 |
| ZC3H12D | 6q25.1 | 0.562804469 | 4.02E-84 | 1.60E-82 |
| SELE | 1q24.2 | 0.562543369 | 4.98E-84 | 1.98E-82 |
| CHRDL1 | Xq23 | 0.562191843 | 6.63E-84 | 2.63E-82 |
| KCNAB2 | 1p36.31 | 0.562160046 | 6.81E-84 | 2.70E-82 |
| IRF1 | 5q31.1 | 0.561506038 | 1.16E-83 | 4.59E-82 |
| SPI1 | 11p11.2 | 0.561000141 | 1.75E-83 | 6.92E-82 |
| GIPC2 | 1p31.1 | 0.560280075 | 3.15E-83 | 1.24E-81 |
| ANXA2R | 5p12 | 0.559680698 | 5.12E-83 | 2.01E-81 |
| FXYD2 | 11q23.3 | 0.559553123 | 5.68E-83 | 2.23E-81 |
| IGFLR1 | 19q13.12 | 0.559528917 | 5.79E-83 | 2.27E-81 |
| DOCK11 | Xq24 | 0.558396299 | 1.45E-82 | 5.65E-81 |
| CCDC178 | 18q12.1 | 0.558296603 | 1.57E-82 | 6.11E-81 |
| DOCK8 | 9p24.3 | 0.558181224 | 1.72E-82 | 6.69E-81 |
| FAM20A | 17q24.2 | 0.558119465 | 1.81E-82 | 7.02E-81 |
| TMEM71 | 8q24.22 | 0.556967671 | 4.56E-82 | 1.77E-80 |
| TGFBR2 | 3p24.1 | 0.556927175 | 4.72E-82 | 1.82E-80 |
| GPR132 | 14q32.33 | 0.556758747 | 5.40E-82 | 2.08E-80 |
| CEACAM4 | 19q13.2 | 0.556596442 | 6.15E-82 | 2.37E-80 |
| ST8SIA4 | 5q21.1 | 0.556051035 | 9.52E-82 | 3.66E-80 |
| INMT | 7p14.3 | 0.555580992 | 1.39E-81 | 5.32E-80 |
| CCL14 | 17q12 | 0.554898482 | 2.39E-81 | 9.16E-80 |
| CASP1 | 11q22.3 | 0.554858645 | 2.47E-81 | 9.43E-80 |
| IGF1 | 12q23.2 | 0.55445429 | 3.40E-81 | 1.30E-79 |
| RELN | 7q22.1 | 0.554198238 | 4.17E-81 | 1.59E-79 |
| TSHR | 14q24-q31 | 0.554015379 | 4.83E-81 | 1.84E-79 |
| ABCA9 | 17q24.2 | 0.553928385 | 5.17E-81 | 1.96E-79 |
| THEMIS2 | 1p35.3 | 0.552868168 | 1.20E-80 | 4.54E-79 |
| PLCXD2 | 3q13.2 | 0.552847187 | 1.22E-80 | 4.61E-79 |
| TMEM119 | 12q23.3 | 0.55244261 | 1.68E-80 | 6.34E-79 |
| PI16 | 6p21.2 | 0.551862783 | 2.65E-80 | 1.00E-78 |
| SHISAL2A | 1p32.3 | 0.55171159 | 2.99E-80 | 1.12E-78 |
| C1QB | 1p36.12 | 0.551314244 | 4.09E-80 | 1.53E-78 |
| LRRC8C | 1p22.2 | 0.550487513 | 7.83E-80 | 2.94E-78 |
| CD300LG | 17q21.31 | 0.549955067 | 1.19E-79 | 4.45E-78 |
| SLFN14 | 17q12 | 0.549888195 | 1.25E-79 | 4.68E-78 |
| PIK3R6 | 17p13.1 | 0.54973103 | 1.42E-79 | 5.28E-78 |
| CD209 | 19p13.2 | 0.54932813 | 1.94E-79 | 7.23E-78 |
| PDE3B | 11p15.2 | 0.549274907 | 2.03E-79 | 7.52E-78 |
| CFH | 1q31.3 | 0.549087215 | 2.35E-79 | 8.69E-78 |
| HAAO | 2p21 | 0.5489239 | 2.67E-79 | 9.86E-78 |
| CD33 | 19q13.41 | 0.548626756 | 3.36E-79 | 1.24E-77 |
| C1R | 12p13.31 | 0.548492056 | 3.73E-79 | 1.38E-77 |
| ANKRD55 | 5q11.2 | 0.548486679 | 3.75E-79 | 1.38E-77 |
| LILRB1 | 19q13.42 | 0.548326403 | 4.25E-79 | 1.56E-77 |
| ITGAD | 16p11.2 | 0.547893011 | 5.96E-79 | 2.18E-77 |
| SIGLEC10 | 19q13.41 | 0.547041086 | 1.16E-78 | 4.22E-77 |
| SH2D2A | 1q23.1 | 0.546914921 | 1.27E-78 | 4.65E-77 |
| PIK3CG | 7q22.3 | 0.546280634 | 2.08E-78 | 7.59E-77 |
| DHRS9 | 2q31.1 | 0.54596615 | 2.66E-78 | 9.66E-77 |
| LY86 | 6p25.1 | 0.545872016 | 2.86E-78 | 1.04E-76 |
| NECAP2 | 1p36.13 | 0.545842884 | 2.92E-78 | 1.06E-76 |
| KBTBD8 | 3p14.1 | 0.545803659 | 3.01E-78 | 1.09E-76 |
| NCF1B | 7q11.23 | 0.545694351 | 3.28E-78 | 1.18E-76 |
| HLA-DPB2 | 6p21.32 | 0.545480042 | 3.87E-78 | 1.39E-76 |
| TLR9 | 3p21.2 | 0.545450117 | 3.96E-78 | 1.42E-76 |
| HLA-DQB2 | 6p21.32 | 0.545098412 | 5.19E-78 | 1.87E-76 |
| KIR3DL2 | 19q13.42 | 0.544922839 | 5.95E-78 | 2.13E-76 |
| GBP1P1 | 1p22.2 | 0.54487368 | 6.17E-78 | 2.21E-76 |
| JAM2 | 21q21.3 | 0.544717135 | 6.97E-78 | 2.49E-76 |
| CHST2 | 3q24 | 0.544174522 | 1.06E-77 | 3.77E-76 |
| TSPAN7 | Xp11.4 | 0.543745628 | 1.47E-77 | 5.23E-76 |
| BTN3A3 | 6p22.2 | 0.54322788 | 2.19E-77 | 7.77E-76 |
| PTCRA | 6p21.1 | 0.543088433 | 2.43E-77 | 8.63E-76 |
| IL15 | 4q31.21 | 0.542637513 | 3.44E-77 | 1.22E-75 |
| LYL1 | 19p13.13 | 0.541404235 | 8.81E-77 | 3.11E-75 |
| BCL2A1 | 15q25.1 | 0.540785646 | 1.41E-76 | 4.98E-75 |
| FNBP1 | 9q34.11 | 0.540553617 | 1.68E-76 | 5.93E-75 |
| CD86 | 3q13.33 | 0.540516894 | 1.73E-76 | 6.08E-75 |
| ADGRE5 | 19p13.12 | 0.540444709 | 1.83E-76 | 6.41E-75 |
| DPPA4 | 3q13.13 | 0.540198948 | 2.20E-76 | 7.72E-75 |
| EGR2 | 10q21.3 | 0.540153598 | 2.28E-76 | 7.97E-75 |
| CASP4 | 11q22.3 | 0.539754091 | 3.08E-76 | 1.08E-74 |
| TPK1 | 7q35 | 0.53949517 | 3.75E-76 | 1.31E-74 |
| BATF3 | 1q32.3 | 0.539482809 | 3.79E-76 | 1.32E-74 |
| PATL2 | 15q21.1 | 0.53895078 | 5.66E-76 | 1.97E-74 |
| CARD6 | 5p13.1 | 0.538819295 | 6.25E-76 | 2.17E-74 |
| RRN3P2 | 16p11.2 | 0.538638284 | 7.17E-76 | 2.48E-74 |
| MATK | 19p13.3 | 0.538534771 | 7.75E-76 | 2.68E-74 |
| HCK | 20q11.21 | 0.537933491 | 1.22E-75 | 4.21E-74 |
| TLR4 | 9q33.1 | 0.537724922 | 1.43E-75 | 4.91E-74 |
| NAALADL1 | 11q13.1 | 0.537340561 | 1.90E-75 | 6.55E-74 |
| PLA1A | 3q13.33 | 0.536796269 | 2.87E-75 | 9.84E-74 |
| B3GALT2 | 1q31.2 | 0.536468427 | 3.66E-75 | 1.26E-73 |
| VNN1 | 6q23.2 | 0.536346659 | 4.01E-75 | 1.37E-73 |
| LIX1L | 1q21.1 | 0.53632496 | 4.08E-75 | 1.39E-73 |
| IL7 | 8q21.13 | 0.535259323 | 9.05E-75 | 3.08E-73 |
| CYBB | Xp21.1-p11.4 | 0.53525313 | 9.09E-75 | 3.09E-73 |
| FAM107A | 3p14.3-p14.2 | 0.535081574 | 1.03E-74 | 3.51E-73 |
| HAPLN3 | 15q26.1 | 0.534384648 | 1.74E-74 | 5.89E-73 |
| TFEC | 7q31.2 | 0.534344942 | 1.79E-74 | 6.05E-73 |
| CD180 | 5q12.3 | 0.533948483 | 2.40E-74 | 8.11E-73 |
| MAP1LC3C | 1q43 | 0.533434454 | 3.52E-74 | 1.19E-72 |
| HLA-DRB6 | 6p21.32 | 0.533419232 | 3.56E-74 | 1.20E-72 |
| RFTN1 | 3p24.3 | 0.532724786 | 5.95E-74 | 2.00E-72 |
| PIM2 | Xp11.23 | 0.531641942 | 1.32E-73 | 4.44E-72 |
| VCAM1 | 1p21.2 | 0.531237416 | 1.78E-73 | 5.97E-72 |
| NLRC5 | 16q13 | 0.530928386 | 2.24E-73 | 7.49E-72 |
| SYT15 | 10q11.22 | 0.530502635 | 3.06E-73 | 1.02E-71 |
| GBP2 | 1p22.2 | 0.530476247 | 3.12E-73 | 1.04E-71 |
| RASSF4 | 10q11.21 | 0.530470156 | 3.13E-73 | 1.04E-71 |
| LAIR2 | 19q13.42 | 0.530238194 | 3.71E-73 | 1.23E-71 |
| TRIM22 | 11p15.4 | 0.530030527 | 4.32E-73 | 1.44E-71 |
| COTL1 | 16q24.1 | 0.529892164 | 4.79E-73 | 1.59E-71 |
| HSD17B11 | 4q22.1 | 0.529801844 | 5.11E-73 | 1.69E-71 |
| TLDC2 | 20q11.23 | 0.529308904 | 7.34E-73 | 2.42E-71 |
| FAM110D | 1p36.11 | 0.529272854 | 7.53E-73 | 2.48E-71 |
| NLRC4 | 2p22.3 | 0.528414057 | 1.41E-72 | 4.64E-71 |
| TYROBP | 19q13.12 | 0.528228932 | 1.61E-72 | 5.30E-71 |
| STK17B | 2q32.3 | 0.527810674 | 2.19E-72 | 7.18E-71 |
| IL18 | 11q23.1 | 0.527659633 | 2.44E-72 | 8.00E-71 |
| NCR1 | 19q13.42 | 0.527177926 | 3.47E-72 | 1.13E-70 |
| BACH2 | 6q15 | 0.526818671 | 4.50E-72 | 1.47E-70 |
| S100B | 21q22.3 | 0.526578904 | 5.35E-72 | 1.74E-70 |
| KIF19 | 17q25.1 | 0.526541424 | 5.50E-72 | 1.79E-70 |
| TBXAS1 | 7q34 | 0.52636064 | 6.27E-72 | 2.04E-70 |
| ZBP1 | 20q13.31 | 0.52571043 | 1.00E-71 | 3.25E-70 |
| STK10 | 5q35.1 | 0.525449237 | 1.21E-71 | 3.92E-70 |
| TSPAN11 | 12p11.21 | 0.525191808 | 1.46E-71 | 4.72E-70 |
| GPA33 | 1q24.1 | 0.525034237 | 1.63E-71 | 5.28E-70 |
| FMNL3 | 12q13.12 | 0.524723444 | 2.04E-71 | 6.59E-70 |
| GBP1 | 1p22.2 | 0.524171506 | 3.04E-71 | 9.79E-70 |
| SCARF1 | 17p13.3 | 0.524023264 | 3.38E-71 | 1.09E-69 |
| PLEKHO1 | 1q21.2 | 0.523817391 | 3.92E-71 | 1.26E-69 |
| CFI | 4q25 | 0.523451607 | 5.10E-71 | 1.63E-69 |
| RHOF | 12q24.31 | 0.523203061 | 6.10E-71 | 1.95E-69 |
| ECSCR | 5q31.2 | 0.523180157 | 6.20E-71 | 1.98E-69 |
| IKZF3 | 17q12-q21.1 | 0.523153987 | 6.31E-71 | 2.01E-69 |
| SGK1 | 6q23.2 | 0.522811498 | 8.07E-71 | 2.57E-69 |
| GLIPR2 | 9p13.3 | 0.522711127 | 8.67E-71 | 2.75E-69 |
| A2M | 12p13.31 | 0.521280992 | 2.41E-70 | 7.63E-69 |
| ADRB2 | 5q32 | 0.52108504 | 2.77E-70 | 8.76E-69 |
| ADGRD1 | 12q24.33 | 0.521014767 | 2.91E-70 | 9.19E-69 |
| GLI1 | 12q13.3 | 0.52081582 | 3.35E-70 | 1.06E-68 |
| ATOH8 | 2p11.2 | 0.519849773 | 6.65E-70 | 2.10E-68 |
| LAT2 | 7q11.23 | 0.519776638 | 7.00E-70 | 2.20E-68 |
| CAVIN2 | 2q32.3 | 0.519772506 | 7.02E-70 | 2.21E-68 |
| DAZL | 3p24.3 | 0.518623152 | 1.58E-69 | 4.96E-68 |
| CD200 | 3q13.2 | 0.518566944 | 1.65E-69 | 5.16E-68 |
| STAMBPL1 | 10q23.31 | 0.518534577 | 1.68E-69 | 5.27E-68 |
| DENND1C | 19p13.3 | 0.518418301 | 1.83E-69 | 5.71E-68 |
| ITGAX | 16p11.2 | 0.518024886 | 2.41E-69 | 7.52E-68 |
| CD300LB | 17q25.1 | 0.517944189 | 2.55E-69 | 7.95E-68 |
| C10ORF105 | 10q22.1 | 0.51793665 | 2.57E-69 | 7.98E-68 |
| PSMB9 | 6p21.32 | 0.517912041 | 2.61E-69 | 8.11E-68 |
| PRAM1 | 19p13.2 | 0.517751042 | 2.93E-69 | 9.06E-68 |
| C2ORF88 | 2q32.2 | 0.516857461 | 5.48E-69 | 1.70E-67 |
| HLA-DQA2 | 6p21.32 | 0.516780465 | 5.79E-69 | 1.79E-67 |
| GTSF1L | 20q13.12 | 0.516430905 | 7.39E-69 | 2.28E-67 |
| BTN3A2 | 6p22.2 | 0.516413076 | 7.49E-69 | 2.30E-67 |
| ZEB2 | 2q22.3 | 0.516156564 | 8.96E-69 | 2.75E-67 |
| GDF10 | 10q11.22 | 0.516043363 | 9.70E-69 | 2.98E-67 |
| FMO2 | 1q24.3 | 0.515801317 | 1.15E-68 | 3.52E-67 |
| BMX | Xp22.2 | 0.515502518 | 1.42E-68 | 4.33E-67 |
| CTSC | 11q14.2 | 0.515280045 | 1.65E-68 | 5.05E-67 |
| COL4A3 | 2q36.3 | 0.515138033 | 1.83E-68 | 5.57E-67 |
| CCDC88B | 11q13.1 | 0.514911607 | 2.14E-68 | 6.51E-67 |
| SLC7A7 | 14q11.2 | 0.514658853 | 2.55E-68 | 7.75E-67 |
| SV2B | 15q26.1 | 0.513480943 | 5.78E-68 | 1.76E-66 |
| TFPI | 2q32.1 | 0.513388212 | 6.16E-68 | 1.87E-66 |
| TOGARAM2 | 2p23.2 | 0.513036098 | 7.87E-68 | 2.38E-66 |
| ANK2 | 4q25-q26 | 0.512565105 | 1.09E-67 | 3.30E-66 |
| DPT | 1q24.2 | 0.512496324 | 1.14E-67 | 3.45E-66 |
| IL6 | 7p15.3 | 0.512246436 | 1.36E-67 | 4.09E-66 |
| TNXB | 6p21.33-p21.32 | 0.511939556 | 1.68E-67 | 5.05E-66 |
| ARHGAP45 | 19p13.3 | 0.511927534 | 1.69E-67 | 5.09E-66 |
| MZB1 | 5q31.2 | 0.511753997 | 1.91E-67 | 5.73E-66 |
| APOL1 | 22q12.3 | 0.511713685 | 1.96E-67 | 5.88E-66 |
| ENG | 9q34.11 | 0.51158103 | 2.15E-67 | 6.43E-66 |
| TKTL1 | Xq28 | 0.511343666 | 2.53E-67 | 7.56E-66 |
| SEL1L3 | 4p15.2 | 0.511253365 | 2.70E-67 | 8.04E-66 |
| DPYS | 8q22.3 | 0.510968783 | 3.28E-67 | 9.76E-66 |
| DUSP2 | 2q11.2 | 0.510962552 | 3.29E-67 | 9.79E-66 |
| LGALS9 | 17q11.2 | 0.510608914 | 4.20E-67 | 1.25E-65 |
| RRN3P1 | 16p12.2 | 0.510122795 | 5.86E-67 | 1.74E-65 |
| SYNE3 | 14q32.13 | 0.509505913 | 8.95E-67 | 2.65E-65 |
| DEPP1 | 10q11.21 | 0.509125489 | 1.16E-66 | 3.43E-65 |
| LILRB3 | 19q13.42 | 0.508583945 | 1.68E-66 | 4.96E-65 |
| C1QC | 1p36.12 | 0.507895384 | 2.69E-66 | 7.92E-65 |
| APOL4 | 22q12.3 | 0.507890593 | 2.70E-66 | 7.93E-65 |
| LBH | 2p23.1 | 0.507880178 | 2.71E-66 | 7.98E-65 |
| ABCA8 | 17q24.2 | 0.507603627 | 3.28E-66 | 9.61E-65 |
| SLFN11 | 17q12 | 0.507549382 | 3.40E-66 | 9.96E-65 |
| AKR1B1 | 7q33 | 0.507425937 | 3.70E-66 | 1.08E-64 |
| EBF1 | 5q33.3 | 0.507207211 | 4.29E-66 | 1.25E-64 |
| LIME1 | 20q13.33 | 0.506938924 | 5.15E-66 | 1.50E-64 |
| CSF1R | 5q32 | 0.506826215 | 5.56E-66 | 1.62E-64 |
| IFFO1 | 12p13.31 | 0.506724828 | 5.95E-66 | 1.73E-64 |
| COL6A6 | 3q22.1 | 0.50623411 | 8.30E-66 | 2.41E-64 |
| CNRIP1 | 2p14 | 0.506089419 | 9.15E-66 | 2.66E-64 |
| LYN | 8q12.1 | 0.50608426 | 9.19E-66 | 2.66E-64 |
| MMRN1 | 4q22.1 | 0.506068034 | 9.29E-66 | 2.69E-64 |
| TNFRSF13C | 22q13.2 | 0.505660229 | 1.22E-65 | 3.53E-64 |
| CARMIL2 | 16q22.1 | 0.50549113 | 1.37E-65 | 3.96E-64 |
| TARP | 7p14.1 | 0.505438983 | 1.42E-65 | 4.09E-64 |
| BHLHE22-AS1 | 8q12.3 | 0.505420506 | 1.44E-65 | 4.14E-64 |
| SSTR3 | 22q13.1 | 0.505412409 | 1.45E-65 | 4.15E-64 |
| TNFSF13B | 13q33.3 | 0.505352969 | 1.51E-65 | 4.32E-64 |
| BTN3A1 | 6p22.2 | 0.505291886 | 1.57E-65 | 4.49E-64 |
| LMO2 | 11p13 | 0.504309399 | 3.04E-65 | 8.70E-64 |
| KIF17 | 1p36.12 | 0.504287781 | 3.09E-65 | 8.82E-64 |
| CCL2 | 17q12 | 0.504234542 | 3.20E-65 | 9.12E-64 |
| NGFR | 17q21.33 | 0.503604343 | 4.89E-65 | 1.39E-63 |
| GLIPR1 | 12q21.2 | 0.503512534 | 5.20E-65 | 1.48E-63 |
| LTK | 15q15.1 | 0.503413146 | 5.56E-65 | 1.58E-63 |
| SLCO5A1 | 8q13.3 | 0.50337673 | 5.69E-65 | 1.61E-63 |
| GPX3 | 5q33.1 | 0.503350574 | 5.80E-65 | 1.64E-63 |
| SLCO2B1 | 11q13.4 | 0.503262015 | 6.15E-65 | 1.74E-63 |
| EBF3 | 10q26.3 | 0.503005583 | 7.30E-65 | 2.06E-63 |
| NR4A3 | 9q22 | 0.502871517 | 7.99E-65 | 2.25E-63 |
| ADCY4 | 14q12 | 0.502212518 | 1.24E-64 | 3.50E-63 |
| C5ORF58 | 5q35.1 | 0.501947462 | 1.48E-64 | 4.17E-63 |
| CYRIA | 2p24.2 | 0.501698856 | 1.75E-64 | 4.91E-63 |
| TNFSF8 | 9q32-q33.1 | 0.501616068 | 1.85E-64 | 5.18E-63 |
| ALDH1A2 | 15q21.3 | 0.501379583 | 2.16E-64 | 6.06E-63 |
| B2M | 15q21.1 | 0.501344827 | 2.22E-64 | 6.19E-63 |
| GHRL | 3p25.3 | 0.501238849 | 2.38E-64 | 6.64E-63 |
| LILRB4 | 19q13.42 | 0.501026184 | 2.74E-64 | 7.63E-63 |
| SYT11 | 1q22 | 0.500960605 | 2.86E-64 | 7.96E-63 |
| STK17A | 7p13 | 0.500813989 | 3.15E-64 | 8.77E-63 |
| MEF2C | 5q14.3 | 0.500532239 | 3.80E-64 | 1.06E-62 |
| MFAP4 | 17p11.2 | 0.500526276 | 3.82E-64 | 1.06E-62 |
| CRHBP | 5q13.3 | 0.500426024 | 4.08E-64 | 1.13E-62 |
| IL9R | Xq28 and Yq12 | 0.500265757 | 4.54E-64 | 1.26E-62 |
| TMEM140 | 7q33 | 0.500145989 | 4.92E-64 | 1.36E-62 |
| LGI2 | 4p15.2 | 0.500133092 | 4.96E-64 | 1.37E-62 |
| AVPR2 | Xq28 | 0.500104434 | 5.05E-64 | 1.39E-62 |

| **Table S5. Correlation analysis between *CD161* and related gene markers of immune cells by GEPIA database** | | | |
| --- | --- | --- | --- |
| Immune cells | Gene markers | Correlation | P values |
| T cell | CD3D | 0.79 | 0 |
|  | CD3E | 0.82 | 0 |
|  | CD2 | 0.81 | 0 |
| CD4^+^ T cell | CD4 | 0.63 | 0 |
| CD8^+^ T cell | CD8A | 0.72 | 0 |
|  | CD8B | 0.56 | 0 |
| Th1 | IFNG | 0.37 | 0 |
|  | TBX21 | 0.73 | 0 |
|  | TNF | 0.09 | 0.0016 |
|  | STAT4 | 0.77 | 0 |
|  | STAT1 | 0.24 | 4.40E-16 |
| Th2 | STAT6 | 0.11 | 0.00031 |
|  | STAT5A | 0.24 | 4.40E-15 |
|  | IL13 | -0.22 | 1.20E-13 |
| Tfh | CXCR5 | 0.42 | 0 |
|  | CXCL13 | 0.09 | 0.0026 |
|  | BCL6 | 0.01 | 0.77 |
|  | IL21 | 0.6 | 0 |
| Th17 | IL17A | 0.13 | 1.10E-05 |
|  | RORC | -0.11 | 0.00042 |
|  | IL23A | 0.10 | 0.00057 |
|  | STAT3 | -0.02 | 0.48 |
| Treg | FOXP3 | 0.56 | 0 |
|  | IKZF2 | 0.12 | 6.30E-05 |
|  | IL10 | 0.36 | 0 |
|  | TGFB1 | 0.24 | 3.60E-15 |
|  | CCR8 | 0.36 | 0 |
|  | STAT5B | 0.14 | 2.90E-06 |
| APC/DC | HLA-DPA1 | 0.56 | 0 |
|  | HLA-DPB1 | 0.60 | 0 |
|  | HLA-DQA1 | 0.47 | 0 |
| B cell | BLK | 0.58 | 0 |
|  | CD19 | 0.58 | 0 |
|  | MS4A1 | 0.55 | 0 |
|  | CD79A | 0.63 | 0 |
| Monocyte | CD86 | 0.49 | 0 |
|  | CD115/CSF1R | 0.32 | 0 |
| TAM | CCL2 | 0.22 | 6.90E-13 |
| M1 | INOS/NOS2 | -0.04 | 0.16 |
|  | IRF5 | 0.22 | 7.30E-14 |
| M2 | CD163 | 0.28 | 0 |
| Neutrophils | CD66B/CEACAM8 | 0.01 | 0.73 |
|  | CD11B/ITGAM | 0.15 | 4.60E-07 |
|  | CCR7 | 0.65 | 0 |
| Natural killer cell | KIR2DL1 | 0.02 | 0.38 |
|  | KIR2DL3 | 0.27 | 0 |
|  | KIR3DL1 | 0.35 | 0 |
|  | KIR3DL2 | 0.35 | 0 |
| T cell exhaustion | PD-1(PDCD1) | 0.59 | 0 |
|  | CTLA-4 | 0.62 | 0 |
|  | LAG3 | 0.26 | 0 |
|  | TIM-3(HAVCR2) | 0.36 | 0 |
|  | GZMB | 0.46 | 0 |
